# Reduction of Intracellular Tension and Cell Adhesion Promotes Open Chromatin Structure and Enhances Cell Reprogramming

**DOI:** 10.1002/advs.202300152

**Published:** 2023-06-26

**Authors:** Jennifer Soto, Yang Song, Yifan Wu, Binru Chen, Hyungju Park, Navied Akhtar, Peng‐Yuan Wang, Tyler Hoffman, Chau Ly, Junren Sia, SzeYue Wong, Douglas O. Kelkhoff, Julia Chu, Mu‐Ming Poo, Timothy L. Downing, Amy C. Rowat, Song Li

**Affiliations:** ^1^ Department of Bioengineering University of California Los Angeles CA 90095 USA; ^2^ Department of Molecular and Cell Biology University of California Berkeley CA 94720 USA; ^3^ Department of Biomedical Engineering University of California Irvine CA 92617 USA; ^4^ Oujiang Laboratory Key Laboratory of Alzheimer's Disease of Zhejiang Province Institute of Aging Wenzhou Medical University Wenzhou Zhejiang 325024 China; ^5^ Department of Integrative Biology and Physiology University of California Los Angeles CA 90095 USA; ^6^ Department of Bioengineering University of California Berkeley CA 94720 USA; ^7^ Department of Medicine University of California Los Angeles CA 90095 USA; ^8^ Eli and Edythe Broad Center of Regenerative Medicine and Stem Cell Research University of California, Los Angeles Los Angeles CA 90095 USA; ^9^ Jonsson Comprehensive Cancer Center David Geffen School of Medicine University of California, Los Angeles Los Angeles CA 90095 USA

**Keywords:** cell adhesion, cytoskeleton, direct reprogramming, epigenetic state

## Abstract

The role of transcription factors and biomolecules in cell type conversion has been widely studied. Yet, it remains unclear whether and how intracellular mechanotransduction through focal adhesions (FAs) and the cytoskeleton regulates the epigenetic state and cell reprogramming. Here, it is shown that cytoskeletal structures and the mechanical properties of cells are modulated during the early phase of induced neuronal (iN) reprogramming, with an increase in actin cytoskeleton assembly induced by Ascl1 transgene. The reduction of actin cytoskeletal tension or cell adhesion at the early phase of reprogramming suppresses the expression of mesenchymal genes, promotes a more open chromatin structure, and significantly enhances the efficiency of iN conversion. Specifically, reduction of intracellular tension or cell adhesion not only modulates global epigenetic marks, but also decreases DNA methylation and heterochromatin marks and increases euchromatin marks at the promoter of neuronal genes, thus enhancing the accessibility for gene activation. Finally, micro‐ and nano‐topographic surfaces that reduce cell adhesions enhance iN reprogramming. These novel findings suggest that the actin cytoskeleton and FAs play an important role in epigenetic regulation for cell fate determination, which may lead to novel engineering approaches for cell reprogramming.

## Graphical abstract

Here, it is shown that cytoskeletal structures and the mechanical properties of cells are modulated during the early phase of induced neuronal (iN) reprogramming. The reduction of actin cytoskeletal tension or cell adhesion at the early phase of reprogramming suppresses the expression of mesenchymal genes, promotes a more open chromatin structure, and significantly enhances the efficiency of iN conversion.

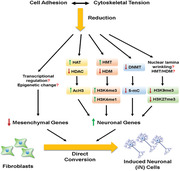

## 1 Introduction

Cell reprogramming enables the derivation of distinct cell types that are highly valuable for regenerative cell therapy, disease modeling and therapeutic discovery.^[^ ^1^, ^2^ ^]^ Direct cell conversion provides a faster and more direct method of generating desired cell types from somatic cells.^[^ ^3^, ^4^ ^]^ Previous studies have demonstrated that fibroblasts can be directly converted into other cell types such as neurons,^[^ ^5^, ^6^ ^]^ and cardiomyocytes^[^ ^7^ ^]^ by using transcription factors, microRNAs and biophysical factors.^[^ ^8^, ^9^, ^10^, ^11^ ^]^ However, low conversion efficiency has limited the translation of direct reprogramming strategies for therapeutic purposes. In addition, the role of mechanotransduction through intracellular structures such as actin cytoskeleton and focal adhesions (FAs) during direct reprogramming is poorly understood.

Increasing evidence indicates that FAs and the cytoskeleton play important roles in sensing and transducing extracellular biophysical signals to modulate intracellular signaling and cell functions.^[^ ^12^, ^13^, ^14^ ^]^ FAs are large, multiprotein complexes that provide a physical link between the extracellular matrix (ECM) and the cytoskeleton. In response to force or biophysical cues, integrins undergo conformational changes that cause the recruitment of distinct FA proteins to the mechanosensing site.^[^ ^15^ ^]^ As a result, cells reorganize their cytoskeleton and generate contractile forces through motor proteins, such as myosin, to fine‐tune their internal tension and reach a state of mechanical stability.^[^ ^16^ ^]^ FAs can be modulated by biochemical and biophysical cues in the cellular microenvironment, and activate signaling pathways that regulate cytoskeletal organization in response to mechanical cues.^[^ ^12^, ^17^ ^]^ In eukaryotic cells, the cytoskeleton, primarily composed of actin microfilaments, intermediate filaments and microtubules, spans the cytoplasm to provide a structural link between the cell nucleus and the ECM. It serves to spatially organize contents of the cell and facilitates cell movement and shape changes through the generation of forces.^[^ ^18^ ^]^ These intracellular structures have been implicated in regulating the mechanical phenotype of cells during many physiological and disease processes.^[^ ^19^, ^20^, ^21^ ^]^ Additionally, there is evidence that the physical coupling of the cell nucleus with the cytoskeleton can affect chromatin structure and regulate the epigenetic state, gene expression and cell function.^[^ ^22^, ^23^ ^]^ Yet, how intracellular structures, such as the actin cytoskeleton and FAs, regulate direct cell reprogramming is still unclear. Furthermore, whether these intracellular structures modulate the epigenetic state to influence direct cell conversion remains unknown.

Here we investigated the role of intracellular tension transmitted through the cytoskeleton and cell adhesion in cell reprogramming using the conversion of fibroblasts into induced neuronal (iN) cells^[^ ^5^ ^]^ as a model. Our results demonstrate that the reduction of intracellular tension in the early phase of the reprogramming can enhance the efficiency of iN conversion by promoting a more open chromatin structure to facilitate the activation of neuronal genes.

## 2 Results

### 2.1 Intracellular Structures and Mechanical Properties of Cells Are Modulated during the Early Phase of iN Reprogramming

**Figure 1:**
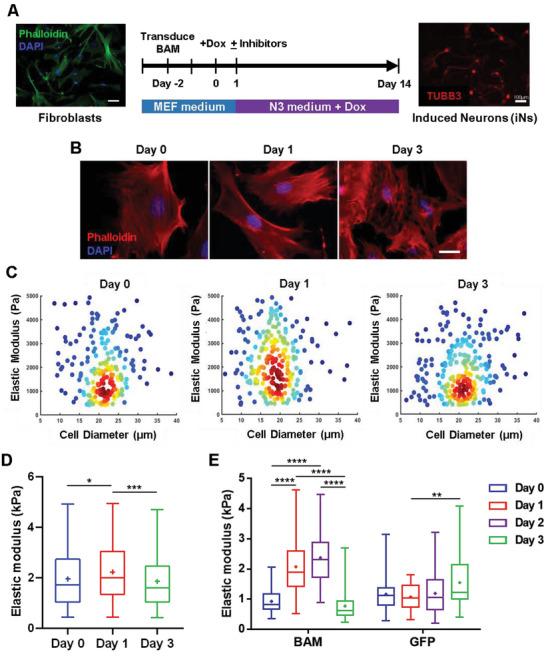
Actin cytoskeleton and cell mechanical phenotype are altered during iN reprogramming. A) Reprogramming protocol. Adult fibroblasts transduced with the reprogramming factors (BAM) were cultured in N3 medium with doxycycline (Dox) for 14 days before immunostaining and quantification. B) Representative images show fluorescence micrograph of the actin network (phalloidin, red) and nucleus (DAPI, blue) in BAM‐transduced fibroblasts at the indicated time points. Scale bars, 20 µm. C) Density scatter plots show the elastic modulus as a function of cell diameter for BAM‐transduced fibroblasts deforming through 9 × 10 µm constrictions at the indicated time points (day 0, *n* = 211; day 1, *n* = 257; day 3, *n* = 253). Dots represent single‐cell data. D) Elastic modulus of BAM‐transduced fibroblasts at the indicated time points (day 0, *n* = 211; day 1, *n* = 257; day 3, *n* = 253) as derived by q‐DC. Significance was determined by a one‐way ANOVA and Tukey's multiple comparison test. E) Box plots illustrate the variation in elastic modulus of BAM‐ or GFP‐transduced fibroblasts at the indicated time points as acquired using AFM, where GFP serves as a control. The number of biological replicates, *n*, was equal to 55 per condition. Significance determined by two‐way ANOVA using Tukey's correction for multiple comparisons. Box plots show the ends at the quartiles, the median as a horizontal line in the box, the mean as a (+) symbol, and the whiskers extend from the minimum to maximum data point (*p < 0.05,**p < 0.01, ***p < 0.001, ****p < 0.0001).

To elucidate the role of the various intracellular structures during iN conversion, primary fibroblasts isolated from adult mice were transduced with doxycycline (Dox)‐inducible lentiviral vectors encoding three key reprogramming factors, *Brn2*, *Ascl1*, and *Myt1l* (BAM), and seeded onto tissue culture polystyrene dishes coated with laminin the following day. As illustrated in **Figure** **1**A, Dox was added one day later (marked as day 0) to initiate the expression of the transgenes and cells were cultured in neuronal medium (i.e., N3 medium) from day 1 to the conclusion of the experiment. To gain insights into the morphological changes that fibroblasts undergo as they reprogram into neurons, we first examined how the actin cytoskeleton was altered during the early phase of iN reprogramming. Interestingly, immunofluorescence analysis revealed that by day 1 of the reprogramming process, actin assembled into a network with a cage‐like structure around the nucleus, but this structure along with the majority of the cytoskeleton gradually disappeared by day 3 (Figure 1B). To determine whether these structural changes resulted in differences in the mechanical phenotype of cells, the mechanical properties of BAM‐transduced fibroblasts was measured at similar time points using high‐throughput quantitative deformability cytometry (q‐DC), in which the timescale of a cell to transit through a narrow constriction provides a metric for cell deformability. We found that cell transit time and stiffness increased by day 1 and was followed by a decrease on day 3, which coincided with the observed cytoskeletal changes (Figure 1C,D and Figures S1 and S2, Supporting Information). Utilizing atomic force microscopy (AFM) to measure cell stiffness yielded a similar trend, consistent with our q‐DC findings, and demonstrated more profound differences in cell stiffness across the various time points (Figure 1E). These changes in cellular mechanical properties were transgene‐specific as transduction with green fluorescent protein (GFP) did not produce a similar effect (Figure 1E). These results suggest that the actin cytoskeleton and mechanical properties of cells are modulated during the early phase of reprogramming, possibly playing a role in iN conversion.

### 2.2 Ascl1 Plays a Dominant Role in Regulating the Cell Mechanical Phenotype Changes

**Figure 2:**
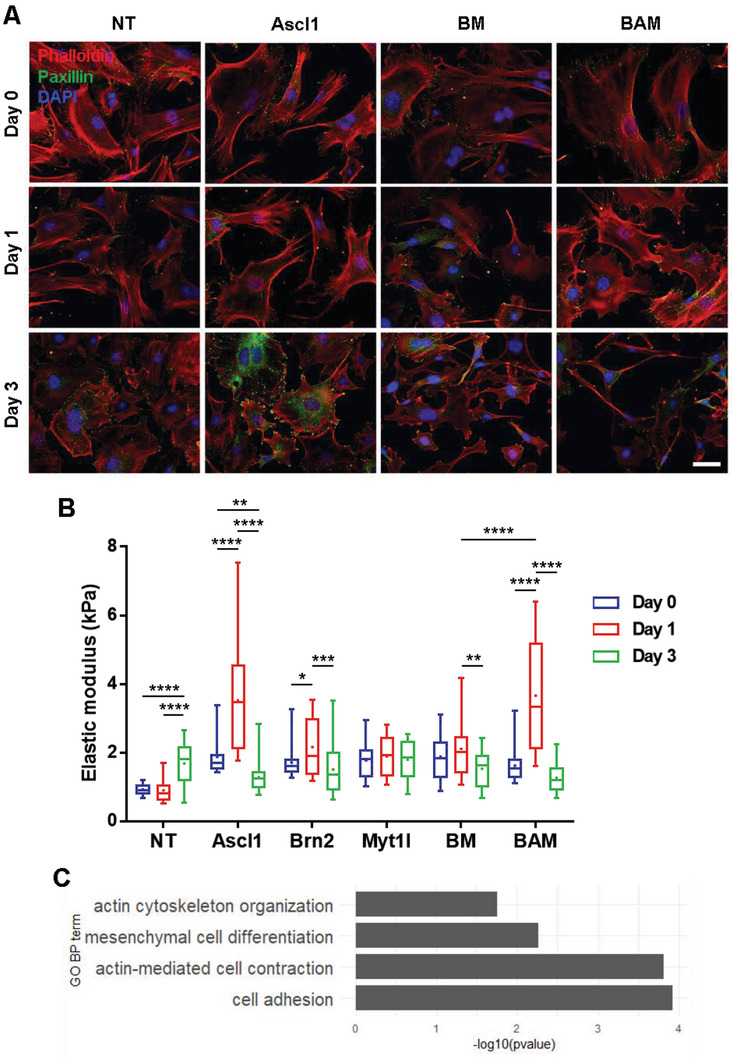
Ascl1 plays a dominant role in regulating cell stiffness and intracellular structures during iN reprogramming. For experiments in (A) and (B), fibroblasts were transduced with individual or various combinations of the transgenes and collected at the indicated time points for immunofluorescence analysis or AFM measurements, where non‐transduced (NT) fibroblasts served as a control. A) Representative immunofluorescent images show the actin network (phalloidin, red), focal adhesions (paxillin, green) and nucleus (DAPI, blue) in fibroblasts transduced with individual or various combinations of the transgenes at the indicated time points. Scale bar, 50 µm. B) Box plots display the distribution of elastic modulus at the indicated time points as acquired using AFM (*n* = 33 per condition). Significance determined by two‐way ANOVA using Tukey's correction for multiple comparisons. C) Genes within the listed gene ontology terms are up‐regulated by Ascl1 overexpression compared to non‐transduced fibroblasts. Box plots show the ends at the quartiles, the median as a horizontal line in the box, the mean as a (+) symbol, and the whiskers extend from the minimum to maximum data point (*p < 0.05, **p < 0.01, ***p < 0.001, ****p < 0.0001).

Next, we sought to determine the transgene that was responsible for the observed changes in the cytoskeleton and mechanical phenotype by reprogramming fibroblasts with individual or various combinations of the transgenes. Immunofluorescence and western blot analysis of cytoskeletal structures and FA proteins demonstrated that Ascl1 promoted the actin cage‐like structure and paxillin expression at day 1 (**Figure** **2**A and Figure S3, Supporting Information). Although less intact stress fibers were observed at day 3, paxillin expression remained high (Figure 2A and Figure S3, Supporting Information). On the other hand, the presence of paxillin‐positive punctate and cell spreading appeared to decrease in BM‐ and BAM‐transduced fibroblasts (Figure 2A). Interestingly, AFM analysis revealed that Ascl1 was the critical transgene in modulating the mechanical phenotype of cells in the early phase of reprogramming as it induced changes in cell stiffness in a manner comparable to BAM (Figure 2B). To elucidate whether these observations could extend to the transcript level, we performed RNA sequencing analysis of non‐transduced and Ascl1‐transduced fibroblasts on day 3. We found that the overexpression of Ascl1 led to the over‐representation of genes in Gene Ontology (GO) categories related to the cytoskeleton and cell adhesion, such as actin cytoskeleton organization and actin‐mediated cell contraction (Figure 2C and Figures S4 and S5, Supporting Information). Altogether, these results suggested that the increase in cytoskeleton assembly at the early phase of reprogramming could be attributed to Ascl1‐induced expression of cytoskeletal proteins.

### 2.3 Reduction of Cytoskeletal Tension Enhances iN Reprogramming

**Figure 3:**
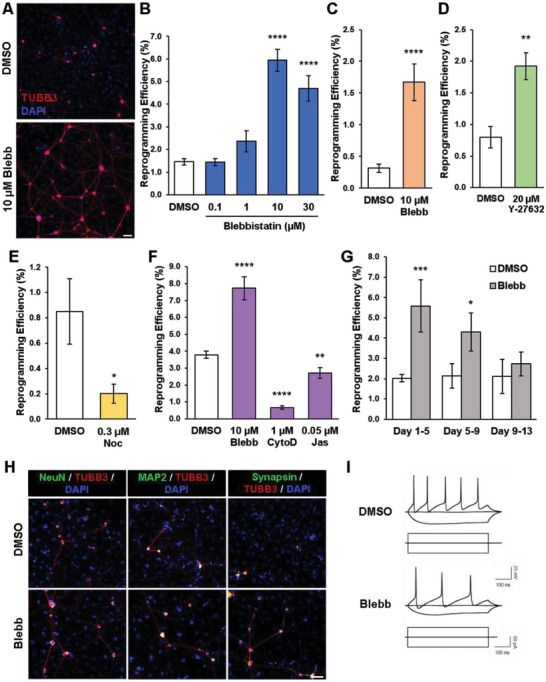
Inhibition of cytoskeletal tension enhances iN conversion. A) Representative fluorescent micrographs of iN cells generated from BAM‐transduced fibroblasts in the absence and presence of 10 µm blebbistatin (denoted as Blebb in this and all subsequent figures). iN cells expressed TUBB3 and formed neural networks. Scale bar, 100 µm. B) Reprogramming efficiency at day 14 of fibroblasts transduced with BAM and cultured in absence and presence of varying concentrations of blebbistatin for 7 days, where DMSO served as a control (*n* = 3). Significance was determined by a one‐way ANOVA and Dunnett's multiple comparison test. C) Reprogramming efficiency of fibroblasts transduced with Ascl1 and cultured in the absence and presence of 10 µm blebbistatin at day 21 (*n* = 6). Significance determined by two‐tailed, unpaired *t*‐test. D) Reprogramming efficiency at day 14 of BAM‐transduced fibroblasts treated with and without the ROCK inhibitor, Y‐27632 (20 µm), for 7 days (*n* = 3). Significance determined by two‐tailed, unpaired *t*‐test. E) Reprogramming efficiency at day 14 of fibroblasts transduced with BAM and cultured in the presence of 0.3 µm Nocodazole (Noc) for 4 days (*n* = 3). Significance determined by two‐tailed, unpaired *t*‐test. F) Reprogramming efficiency at day 14 of BAM‐transduced fibroblasts cultured in the absence and presence of 10 µm blebbistatin, 1 µm cytochalasin D (CytoD), and 0.05 µm jasplakinolide (Jas) (*n* = 3). Significance was determined by a one‐way ANOVA and Dunnett's multiple comparison test. G) Reprogramming efficiency at day 14 of BAM‐transduced fibroblasts treated with 10 µm blebbistatin during the early (i.e., days 1–5), mid (i.e., days 5–9), and late (i.e., days 9–13) phases of reprogramming (*n* = 3). Significance determined by two‐way ANOVA using Sidak's multiple comparison test. H) Representative fluorescent images of TUBB3^+^ iN cells co‐expressing mature neuronal markers, NeuN, MAP2 and synapsin at day 21. Scale bar, 100 µm. I) Representative traces of spontaneous changes in membrane potential in response to current injection from iN cells obtained in the presence and absence of 10 µm blebbistatin. Bar graphs show mean ± standard deviation (*p < 0.05, **p < 0.01, ***p < 0.001, ****p < 0.0001).

To examine whether these changes in cell stiffness, actin structure, and expression of contractility‐mediating genes were involved in iN reprogramming, we evaluated the effects of disrupting the cytoskeleton using small molecule inhibitors. For these experiments, BAM‐transduced fibroblasts were reprogrammed as in Figure 1A and chemical compounds known to perturb the cytoskeleton were added to the culture medium on day 1 to evaluate their effects on the reprogramming process. After 2 weeks, cultures were fixed and immunostained for neuronal *β*‐III tubulin (TUBB3). iN cells were identified based on displays of a typical neuronal morphology (defined as cells with a circular soma extending processes that are at least three times the length of the cell body) and positive TUBB3 expression. The reprogramming efficiency was determined as the percentage of TUBB3^+^‐iN cells normalized to the number of cells plated at 24 h post‐seeding (i.e., day 0). First, we tested whether disruption of cytoskeletal contractility could influence the direct reprogramming of fibroblasts into neurons using blebbistatin, a non‐muscle myosin II inhibitor.^[^ ^24^, ^25^, ^26^ ^]^ We observed that iN cells could be derived in the absence and presence of blebbistatin (**Figure** **3**A). Interestingly, when fibroblasts were induced to reprogram in the presence of varying concentrations of blebbistatin, we observed a biphasic dose response, suggesting that a reduction in intracellular tension may promote iN conversion (Figure 3B). In particular, treatment with 10 µm blebbistatin generated the highest number of iN cells and increased the reprogramming efficiency by ≈4.5‐fold compared to the control, therefore, this concentration was used in all subsequent experiments. This enhanced reprogramming efficiency by blebbistatin was also confirmed by using mouse fibroblasts isolated from Tau‐EGFP mice, whereby cells expressing the neuronal marker, Tau, are concomitantly labeled with GFP (Figure S6, Supporting Information). Furthermore, small interfering RNA (siRNA) knockdown of non‐muscle myosin heavy chain IIA (*MYH9*) and IIB (*MYH10*) in BAM‐transduced fibroblasts promoted the reprogramming efficiency, recapitulating the small molecule inhibitor results, although to a lesser degree, and suggesting that a reduction in non‐muscle myosin II may be responsible for the observed increase in the reprogramming efficiency (Figures S7 and S8, Supporting Information). As Ascl1 has been identified as a pioneer factor for iN reprogramming and transduction of Ascl1 alone is sufficient to generate iN cells,^[^ ^27^, ^28^ ^]^ we further investigated whether blebbistatin could influence iN conversion by Ascl1 induction. Remarkably, we found that blebbistatin treatment could generate significantly more iN cells from Ascl1‐transduced fibroblasts relative to control (Figure 3C), suggesting that inhibition of cell contractility could promote single‐factor reprogramming.

To determine whether cytoskeletal disruption modulated intracellular tension, we used AFM to measure cell stiffness as an indicator of intracellular tension.^[^ ^29^, ^30^ ^]^ We found a decrease in cytoskeletal and nuclear stiffness after blebbistatin treatment, suggesting that inhibition of cytoskeletal contractility indeed decreased intracellular tension (Figure S9, Supporting Information). To determine whether interference of other cytoskeletal structures affected iN reprogramming, we examined the effects of other chemical inhibitors on actin cytoskeleton and reprogramming, including Y‐27632 (a Rho‐kinase inhibitor to prevent stress fiber formation and contraction),^[^ ^31^ ^]^ nocodazole (disrupting the assembly and disassembly dynamics of microtubules),^[^ ^32^ ^]^ jasplakinolide (stabilizing actin filaments),^[^ ^12^ ^]^ and cytochalasin D (inhibiting F‐actin polymerization). Consistently, inhibition Rho‐kinase decreased intracellular tension (Figure S9, Supporting Information), decreased cell spreading (Figure S10, Supporting Information), and increased the yield of iN cells similar to blebbistatin, although to a lesser degree (Figure 3D); in contrast, treatment with nocodazole, which compromised cell division and other functions and increased stress fibers (Figure S10, Supporting Information), resulted in a reduction in the reprogramming efficiency (Figure 3E). Moreover, treatment with jasplakinolide or cytochalasin D reduced the reprogramming efficiency relative to control (Figure 3F and Figure S10, Supporting Information), suggesting that maintaining a certain level of actin cytoskeleton might be required for iN reprogramming.

Next, we determined the minimum length of time necessary for the inhibitor to elicit an effect by reprogramming fibroblasts in the absence and presence of blebbistatin over timescales of 1 to 14 days. Surprisingly, we found that administering blebbistatin for the first 3 days was sufficient to enhance the reprogramming efficiency, suggesting that relaxation of intracellular tension might facilitate initiation of the reprogramming process (Figure S11, Supporting Information). Indeed, treatment with blebbistatin during distinct phases, that is, early (days 1–5), mid (day 5–9), and late (day 9–13) stages of reprogramming, demonstrated that the effects of cytoskeletal disruption were most crucial during the early phase of reprogramming (Figure 3G). Additionally, time course studies of the reprogramming process revealed that as early as day 5, blebbistatin treatment led to more efficient generation of TUBB3^+^ iN cells than the control, and this trend was evident throughout a longer time period (Figures S12 and S13, Supporting Information).

To determine whether the reduction of cytoskeletal tension at the early stage of reprogramming adversely affected neuronal properties, we assessed the maturation and functionality of the iN cells derived in the absence and presence of blebbistatin. Immunostaining analysis for mature neuronal markers revealed that iN cells expressed NeuN, microtubule‐associated protein 2 (MAP2), and synapsin (Figure 3H). Similar observations were found in Tau‐EGFP‐derived iN cells (Figure S14, Supporting Information). Electrophysiological analysis indicated that the iN cells were functional, exhibiting spontaneous changes in membrane potential in response to current injection (Figure 3I). Further analysis of several action potential properties showed no apparent difference for iN cells generated in the absence and presence of blebbistatin (Figure S15A–D, Supporting Information). In addition, there was no significant difference in the frequency of spontaneous excitatory postsynaptic currents (EPSCs), but the EPSC amplitude was 12% higher in iN cells derived with blebbistatin (Figure S15E–G, Supporting Information). Similarly, we observed no significant difference in spontaneous inhibitory post synaptic currents (IPSCs) frequency, although the amplitude of IPSC was 4% lower in blebbistatin‐derived iN cells (Figure S15H–J, Supporting Information). Furthermore, iN cells were found to be of the GABAergic and glutamatergic subtypes, in agreement with a previous report^[^ ^5^ ^]^ (Figure S16, Supporting Information). Collectively, these results suggest that blebbistatin treatment did not affect the maturation and general functions of iN cells except for a slight (4–12%) effect on the amplitude of EPSCs and IPSCs.

### 2.4 Cytoskeletal Disruption Modulates the Expression of Fibroblast and Neuronal Markers

**Figure 4:**
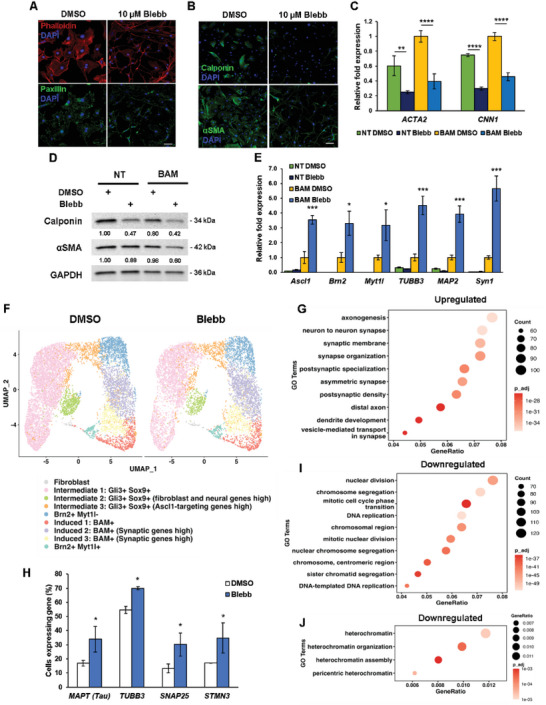
Cytoskeletal tension inhibition regulates the expression of mesenchymal and neuronal markers in the early phase of reprogramming. A) Images show fluorescence micrograph of the actin network (phalloidin, red), focal adhesions (paxillin, green) and nucleus (DAPI, blue) of fibroblasts treated with 10 µm blebbistatin for 24 h. Scale bar, 50 µm. B) Immunofluorescent staining of calponin and *α*SMA in non‐transduced fibroblasts after blebbistatin treatment for 24 h. Scale bar, 100 µm. C) qRT‐PCR analysis of *ACTA2* and *CNN1* expression at day 3 from non‐transduced (NT) and BAM‐transduced fibroblasts cultured in the absence and presence of blebbistatin for 2 days (*n* = 3). Expression level normalized to BAM‐transduced fibroblasts treated with DMSO. Significance determined by one‐way ANOVA and Sidak's multiple comparison test, compared to the corresponding DMSO condition for the same gene. D) NT or BAM‐transduced fibroblasts were treated with blebbistatin for 2 days, followed by western blot analysis of mesenchymal markers, *α*SMA and calponin. GAPDH is shown as a loading control. E) qRT‐PCR analysis of neuronal gene expression at day 5 from NT and BAM transduced fibroblasts cultured in the absence and presence of blebbistatin for 4 days (*n* = 3). Expression level normalized to BAM‐transduced fibroblasts treated with DMSO. Significance determined by two‐tailed, unpaired *t*‐test, compared to the DMSO condition for transduced cells for the same gene. F) BAM‐transduced fibroblasts reprogrammed in the absence or presence of blebbistatin were collected on day 3 for 10x single cell RNA sequencing (scRNA‐seq) analysis. Uniform manifold approximation and projection (UMAP) plot analysis showing 9 cell clusters based on gene expression profile (each point represents a single cell). G) Genes within the listed gene ontology (GO) terms are upregulated in BAM‐transduced fibroblasts treated with blebbistatin on day 3 compared to the DMSO condition based on scRNA‐seq. H) Quantification of the percentage of cells expressing selected neuronal genes based on scRNA‐seq analysis (*n* = 2). Significance determined by two‐way ANOVA and Sidak's multiple comparison test, compared to the corresponding DMSO condition for the same gene. I,J) Genes within the listed gene ontology terms are downregulated in BAM‐transduced fibroblasts treated with blebbistatin compared to the DMSO condition, as determined by scRNA‐seq. Bar graphs show mean ± standard deviation (*p < 0.05, **p < 0.01, ***p < 0.001, ****p < 0.0001).

To gain insights into the mechanism by which disruption of cell contractility enhanced the reprogramming efficiency, we first examined the effects of blebbistatin on cell morphology. We observed that blebbistatin induced dramatic changes in cell morphology and reduced cell spreading in fibroblasts treated with blebbistatin for 24 h (**Figure** **4**A). In addition, there appeared to be fewer intact actin fibers in fibroblasts treated with blebbistatin. Upon observing that cell spreading was affected, immunostaining analysis of FA proteins showed there were fewer positive paxillin punctate in blebbistatin‐treated cells, suggesting that cytoskeletal disruption may modulate FA assembly and, as a result, cell adhesion (Figure 4A).

It has been proposed that cell reprogramming involves the suppression of the original cell phenotype and the activation of the target cell fate regulatory program.^[^ ^33^ ^]^ Thus, we investigated whether blebbistatin's mechanism of action involved the repression of the mesenchymal phenotype in fibroblasts. To test this, non‐transduced fibroblasts were cultured with and without blebbistatin for 24 or 48 h, respectively, followed by analysis of mesenchymal marker expression. Interestingly, we observed less calponin and *α*‐smooth muscle actin (SMA)‐positive cells after blebbistatin treatment and that the expression for both markers had decreased at the gene and protein level by day 3 (Figure 4B–D). Similarly, this trend was evident in BAM‐transduced fibroblasts (Figure 4C,D). Quantitative real‐time polymerase chain reaction (qRT‐PCR) analysis also showed that blebbistatin downregulated the expression of other mesenchymal and fibroblast‐associated genes, *ELN* and *DCN*, in non‐ and BAM‐transduced fibroblasts (Figure S17, Supporting Information).

Subsequently, we explored the effect of cytoskeletal disruption on the induction of the neuronal phenotype by performing qRT‐PCR analysis to evaluate neuronal gene expression at day 5. We found that the expression of various neuronal genes, including the key reprogramming factors, were significantly increased in BAM‐transduced fibroblasts treated with blebbistatin, as compared to the non‐treated transduced cells (Figure 4E). It is important to note that, in the absence of BAM transgenes, blebbistatin was not sufficient to induce endogenous neuronal genes in the fibroblasts (Figure 4E), implicating that blebbistatin either directly enhanced transgenes or modulated the epigenetic state during reprogramming. To determine whether blebbistatin influenced the expression of the transgenes at an earlier time point, we analyzed the expression of BAM at day 3. qRT‐PCR analysis revealed that *Ascl1* expression was upregulated by blebbistatin at day 3 (Figure S18, Supporting Information). On the contrary, *Brn2* and *Myt1l* did not show such a drastic increase in the expression level, suggesting that the reduction of cytoskeletal tension may enhance iN conversion efficiency by modulating *Ascl1* expression at this early time point. Considering two additional transcription factors along with Ascl1 are included in the reprogramming cocktail to generate iN cells, we questioned if perhaps blebbistatin could induce the expression of *Brn2* and *Myt1l* to promote single factor reprogramming. Utilizing qRT‐PCR to analyze the changes in neuronal gene expression in Ascl1‐transduced fibroblasts, we found that blebbistatin upregulated the expression of endogenous *Brn2* and *Myt1l* at day 7 (Figure S19, Supporting Information). Moreover, the expression of several neuronal genes, including the master neuronal gene, *NeuroD1*, was also higher in Ascl1‐transduced fibroblasts treated with blebbistatin (Figure S19, Supporting Information).

To determine the effect of reducing cytoskeletal tension on gene expression at the single‐cell level, we performed 10x single cell RNA sequencing (scRNA‐seq) on samples of BAM‐transduced fibroblasts reprogrammed in the absence or presence of blebbistatin on day 3. Uniform manifold approximation and projection (UMAP) plot analysis identified 9 cell clusters based on gene expression profile, which were comparable between DMSO‐ and blebbistatin‐treated cells (Figure 4F and Figures S20 and S21, Supporting Information). Further analysis revealed that blebbistatin treatment differentially regulated gene expression, in particular, promoting the expression of genes related to axonogenesis and synapse organization/regulation (Figure 4G and Figures S22 and S23, Supporting Information). Additionally, we found an increase in the percentage of cells expressing neuronal genes, including *TUBB3* and *MAPT*, after blebbistatin treatment, which is in agreement with our reprogramming efficiency findings and further suggests that a reduction in cytoskeletal tension can promote iN reprogramming (Figure 4H and Figure S24, Supporting Information). Further GO term analysis showed that blebbistatin induced the downregulation of mitosis‐related and heterochromatin‐related genes when compared to the DMSO condition (Figure 4I,J). Utilizing Monocle 3 to construct a single cell trajectory based on gene expression, we found that blebbistatin‐derived iN cells follow a similar reprogramming trajectory as those derived in the absence of blebbistatin (Figure S25, Supporting Information). In short, the reduction of intracellular tension may promote BAM‐induced iN reprogramming by globally upregulating numerous neuronal genes at the single cell level.

### 2.5 Reduction of Cytoskeletal Tension Promotes a More Opened Chromatin Structure Globally and Locally

**Figure 5:**
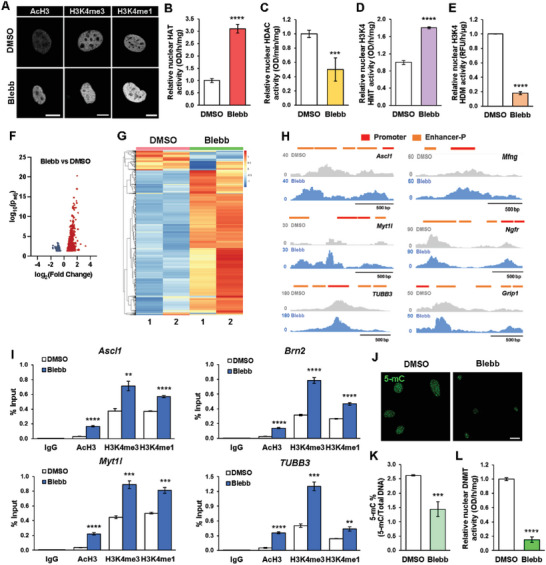
Cytoskeleton disruption modulates the epigenetic state to promote iN reprogramming. A) Representative images show the fluorescence intensity of histone modifications in non‐transduced fibroblasts treated with 10 µm blebbistatin for 2 h. Scale bar, 10 µm. B) Quantification of histone acetyltransferase (HAT) activity in fibroblasts treated with DMSO or 10 µm blebbistatin for 2 h (*n* = 5). Significance determined by two‐tailed, unpaired *t*‐test, compared to DMSO condition. C) Quantification of histone deacetylase (HDAC) activity in fibroblasts treated with DMSO or 10 µm blebbistatin for 2 h (*n* = 5). Significance determined by two‐tailed, unpaired *t*‐test, compared to DMSO condition. D) Quantification of H3K4‐specific histone methyltransferase (HMT) activity in fibroblasts treated with DMSO or 10 µm blebbistatin for 2 h (*n* = 3). Significance determined by two‐tailed, unpaired *t*‐test, compared to DMSO condition. E) Quantification of H3K4‐specific histone demethylase (HDM) activity in fibroblasts treated with DMSO or 10 µm blebbistatin for 2 h (*n* = 3). Significance determined by two‐tailed, unpaired *t*‐test, compared to DMSO condition. F) Volcano plot showing differential accessible regions in blebbistatin‐treated fibroblasts relative to DMSO‐treated fibroblasts, as determined by ATAC‐seq. Red dots indicate regions with increased chromatin accessibility while blue dots indicate regions with decreased accessibility. G) Heatmap representation of differentially accessible regions in fibroblasts treated with DMSO or blebbistatin for 2 h, as determined by ATAC‐seq. Each row represents a differential region; each column is one biological replicate of the indicated condition. H) ATAC‐seq tracks for *Ascl1*, *Myt1l*, *TUBB3, Mfng, Ngfr*, and *Grip1* genomic loci from fibroblasts treated with DMSO or blebbistatin for 2 h, highlighting promoter and proximal enhancer (Enhancer‐P) regions. I) ChIP‐qPCR analysis shows the percent input increase of histone modifications at the promoter regions of *Ascl1*, *Brn2*, *Myt1l*, and *TUBB3* in BAM‐transduced fibroblasts cultured with DMSO or 10 µm blebbistatin at day 3 (*n* = 3). Significance determined by two‐tailed, unpaired *t*‐test, compared to DMSO condition. J) Representative images of 5‐mC expression in non‐transduced fibroblasts treated with blebbistatin for 24 h. Scale bar, 20 µm. K) Quantification of percentage of methylated DNA (5‐mC) in total DNA from DNA samples of fibroblasts cultured in the absence and presence of blebbistatin for 24 h (*n* = 4). Significance determined by two‐tailed, unpaired *t*‐test, compared to DMSO condition. L) Quantification of DNA methyltransferase (DNMT) activity in fibroblasts treated with DMSO or 10 µm blebbistatin for 2 h (*n* = 5). Significance determined by two‐tailed, unpaired *t*‐test, compared to DMSO condition. Bar graphs show mean ± standard deviation (**p < 0.01, ***p < 0.001, ****p < 0.0001).

A critical step of cell reprogramming is to overcome the epigenetic barrier. We postulated that the reduction of cytoskeletal tension might alter the epigenetic state to promote iN conversion. Consistently, scRNA‐seq showed that blebbistatin downregulated the expression of heterochromatin genes (Figure 4J). Therefore, to determine the effect of cytoskeletal modulation on global chromatin organization, we performed immunofluorescence analysis of histone marks associated with open chromatin structure (i.e., histone H3 acetylation [AcH3], tri‐methylated histone H3 on lysine 4 [H3K4me3], and mono‐methylated histone H3 on lysine 4 [H3K4me1]) and indicative of heterochromatin (i.e., tri‐methylated histone H3 on lysine 27 [H3K27me3] and tri‐methylated histone H3 on lysine 9 [H3K9me3]) in non‐transduced fibroblasts cultured with and without blebbistatin. As shown in **Figure** **5**A and Figure S26, Supporting Information, interestingly, blebbistatin‐treated cells exhibited an increase in AcH3, H3K4me3, and H3K4me1 marks while heterochromatin marks H3K27me3 and H3K9me3 decreased, compared to control, suggesting that inhibition of cytoskeletal contractility can induce global epigenetic changes and furthermore, may promote a more open chromatin structure. Similar histone trends were observed in fibroblasts treated with the Rho‐kinase inhibitor (Y‐27632) but no other cytoskeletal disrupting compounds (i.e., nocodazole, cytochalasin D, and jasplakinolide), suggesting that these histone changes might be attributed to changes in cytoskeletal tension (Figures S27 and S28, Supporting Information).

Analysis of chromatin‐modifying enzyme activity showed that blebbistatin treatment increased histone acetyltransferase (HAT) activity while reducing histone deacetylase (HDAC) activity (Figure 5B,C), which could potentially lead to an increase in histone H3 acetylation and thus, gene activation. In addition, blebbistatin treatment increased the activity of H3K4‐specific histone methyltransferase (HMT) and reduced histone demethylase (HDM) activity, thereby possibly promoting H3K4 methylation (Figure 5D,E).

To directly determine whether intracellular tension reduction could alter chromatin accessibility at specific sites of chromatin, we performed the assay of transposase accessible chromatin sequencing (ATAC‐seq) and found that blebbistatin treatment not only differentially regulated global chromatin accessibility, but also specifically increased the accessibility at the promoter or enhancer regions of neuronal genes, including *Ascl1*, *Myt1l*, and *Tubb3*, and Ascl1‐target genes (*Mfng*, *Ngfr*, *Grip1*) (Figure 5F–H and Figures S29 and S30, Supporting Information). Interestingly, the reduction of cytoskeletal tension slightly decreased the accessibility at the promoters or enhancer regions of some mesenchymal genes (Figure S31, Supporting Information). To elucidate whether the observed epigenetic changes in active chromatin marks were involved in regulating neuronal gene expression during iN reprogramming, we performed a chromatin immunoprecipitation‐quantitative polymerase chain reaction (ChIP‐qPCR) assay at the promoter regions of *Ascl1*, *Brn2*, *Myt1l*, and *TUBB3*. ChIP‐qPCR analysis revealed significant increases in AcH3, H3K4me3 and H3K4me1 at all four promoter regions in blebbistatin‐treated cells relative to DMSO (Figure 5I). Further examination of other epigenetic mechanisms that may be affected by inhibition of intracellular tension revealed a decrease in the abundance of DNA methylation marker, 5‐methylcytosine (5‐mC), in blebbistatin‐treated cells relative to the control (Figure 5J,K). These findings were consistent with a decrease in DNA methyltransferase (DNMT) activity after blebbistatin treatment (Figure 5L). Altogether, these results suggest that cytoskeletal disruption can reduce DNA methylation and promote global and site‐specific changes in histone H3 acetylation and methylation that are conducive for neuronal gene activation.

### 2.6 Inhibition of Focal Adhesion Kinase Improves iN Cell Generation

**Figure 6:**
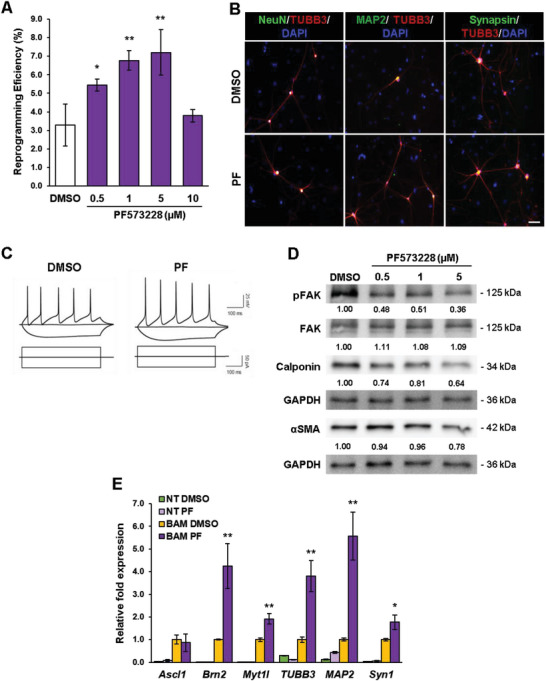
Optimal level of focal adhesion kinase inhibition improves iN cell generation. A) Reprogramming efficiency of BAM‐transduced fibroblasts cultured in the absence and presence of various concentrations of the FAK inhibitor, PF573228 (denoted as PF in this figure) at day 14 (*n* = 3). Significance was determined by a one‐way ANOVA and Dunnett's multiple comparison test. B) Immunofluorescent images of TUBB3^+^ iN cells derived in the presence of 1 µm PF573228 (PF) displaying a typical neuronal morphology and co‐expressing mature neuronal markers, NeuN, MAP2 and synapsin at day 21. Scale bar, 50 µm. C) Representative traces of spontaneous changes in membrane potential in response to current injection from iN cells obtained in the absence and presence of 1 µm PF573228. The inhibitor was administered during the first 7 days of reprogramming. D) Western blot analysis of FAK and mesenchymal marker expression in BAM‐transduced fibroblasts that were treated with various concentrations of PF573228 for 2 days. E) qRT‐PCR analysis of neuronal gene expression at day 5 from non‐transduced (NT) and BAM‐transduced fibroblasts cultured in the absence and presence of 5 µm PF573228 for 4 days (*n* = 3). Expression level normalized to BAM‐transduced fibroblasts treated with DMSO. Significance determined by two‐tailed, unpaired *t*‐test, compared to the DMSO condition for transduced cells for the same gene. Bar graphs show mean ± standard deviation (*p < 0.05, **p < 0.01).

We then explored the modulation of intracellular tension via cell‐ECM adhesions. Specifically, we examined the role of FA signaling by blocking the activity with the focal adhesion kinase (FAK) inhibitor, PF573228, which we found could alter intracellular tension by decreasing cell and nuclear stiffness (Figure S9, Supporting Information). Western blot analysis showed that PF573228 inhibited the phosphorylation of FAK (pFAK) at Tyrosine 397 (Tyr‐397) in a dose‐dependent manner and in addition, modulated downstream ERK signaling (Figure S32A, Supporting Information), demonstrating the specificity of the inhibitor. Immunostaining analysis also confirmed that PF573228 reduced pFAK expression in fibroblasts (Figure S32B, Supporting Information). Thereafter, BAM‐transduced fibroblasts were induced to reprogram in the absence and presence of varying concentrations of PF573228 to test the effects of FAK inhibition on iN conversion. Interestingly, FAK inhibition significantly increased the reprogramming efficiency in a biphasic manner, similar to blebbistatin treatment, suggesting that a reduction of FAs to an optimal level may facilitate iN conversion (**Figure** **6**A). Although FAK inhibition via PF573228 may influence ERK signaling, we found that the conversion efficiency was not significantly altered when BAM‐transduced fibroblasts were treated with various doses of the ERK inhibitor, U0126, suggesting that the inhibition of ERK might not account for the major effect of FAK inhibition on iN reprogramming (Figure S32C, Supporting Information). Further characterization of the iN cells derived in absence and presence of the FAK inhibitor showed that these cells expressed neuronal markers, including NeuN, MAP2, and synapsin, and exhibited functional neuronal properties as assessed by electrophysiological analysis that were comparable to blebbistatin‐induced iN cells (Figure 6B,C and Figure S33, Supporting Information).

**Figure 7:**
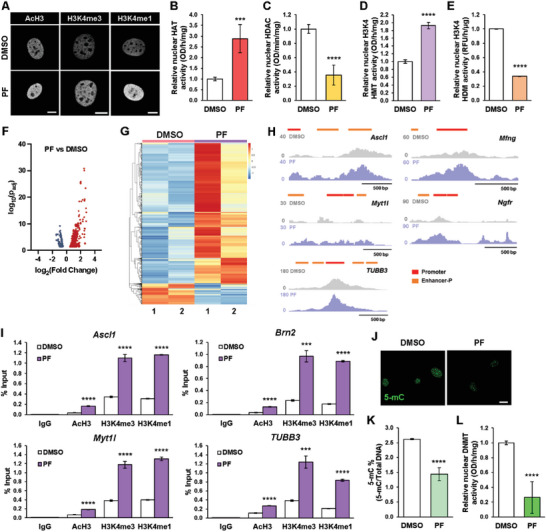
Focal adhesion kinase inhibition modulates the epigenetic state to enhance iN reprogramming. A) Representative images of histone modifications in non‐transduced fibroblasts after treatment with 5 µm PF573228 for 2 h. Scale bar, 10 µm. B) Quantification of histone acetyltransferase (HAT) activity in fibroblasts treated with DMSO or 5 µm PF573228 for 2 h (*n* = 5). Significance determined by two‐tailed, unpaired *t*‐test, compared to DMSO condition. C) Quantification of histone deacetylase (HDAC) activity in fibroblasts treated with DMSO or 5 µm PF573228 for 2 h (*n* = 5). Significance determined by two‐tailed, unpaired *t*‐test, compared to DMSO condition. D) Quantification of H3K4‐specific histone methyltransferase (HMT) activity in fibroblasts treated with DMSO or 5 µm PF573228 for 2 h (*n* = 3). Significance determined by two‐tailed, unpaired *t*‐test, compared to DMSO condition. E) Quantification of H3K4‐specific histone demethylase (HDM) activity in fibroblasts treated with DMSO or 5 µm PF573228 for 2 h (*n* = 3). Significance determined by two‐tailed, unpaired *t*‐test, compared to DMSO condition. F) Volcano plot showing differential accessible regions in PF‐treated fibroblasts relative to DMSO‐treated fibroblasts, as determined by ATAC‐seq. Red dots indicate regions with increased chromatin accessibility while blue dots represent regions with decreased accessibility. G) Heatmap representation of differentially accessible regions in fibroblasts treated with DMSO or PF573228 for 2 h, as determined by ATAC‐seq. Each row represents a differential region; each column is one biological replicate of the indicated condition. H) ATAC‐seq tracks for *Ascl1*, *Myt1l*, *TUBB3, Mfng*, and *Ngfr* genomic loci from fibroblasts treated with DMSO or 5 µm PF for 2 h, highlighting promoter and proximal enhancer (Enhancer‐P) regions. I) ChIP‐qPCR analysis shows the percent input increase of histone modifications at the promoter regions of *Ascl1*, *Brn2*, *Myt1l*, and *TUBB3* in BAM‐transduced fibroblasts cultured with DMSO or 5 µm PF573228 at day 3 (*n* = 3). Significance determined by two‐tailed, unpaired *t*‐test, compared to DMSO condition. J) Representative images of 5‐mC expression in non‐transduced fibroblasts treated with 5 µm PF573228 for 24 h. Scale bar, 20 µm. K) Quantification of percentage of methylated DNA (5‐mC) in total DNA from DNA samples of fibroblasts cultured in the absence and presence of PF573228 for 24 h (*n* = 4). Significance determined by two‐tailed, unpaired *t*‐test, compared to DMSO condition. L) Quantification of DNA methyltransferase (DNMT) activity in fibroblasts treated with DMSO or 5 µm PF573228 for 2 h (*n* = 5). Significance determined by two‐tailed, unpaired *t*‐test, compared to DMSO condition. Bar graphs show mean ± standard deviation (***p < 0.001, ****p < 0.0001).

We further examined whether FAK inhibition could modulate mesenchymal and neuronal marker expression during iN conversion. Indeed, we found that expression of calponin, *α*SMA, and other mesenchymal markers decreased in BAM‐transduced fibroblasts that were treated with PF573228 for 2 days (Figure 6D and Figures S34 and S35, Supporting Information). Conversely, neuronal gene expression was greater in fibroblasts transduced with BAM and cultured in the presence of 5 µm PF573228 at day 5, relative to the control (Figure 6E). As with the reduction of intracellular tension, perturbations of cell adhesions also modulated the epigenetic state. Immunofluorescence analysis revealed that fibroblasts treated with the FAK inhibitor exhibited global increases in AcH3, H3K4me3 and H3K4me1 marks and a concurrent decrease in H3K9me3 and H3K27me3 compared to the control cells (**Figure** **7**A and Figure S36, Supporting Information), which coincided with differences in HAT, HDAC, H3K4‐specific HMT, and H3K4‐specific HDM activity (Figure 7B–E). ATAC‐seq analysis further revealed that FAK inhibition could increase chromatin accessibility, in particular at the promoter or enhancer regions of neuronal genes (*Ascl1*, *Myt1l*, and *Tubb3*) and Ascl1‐target sites (*Mfng*, *Ngfr*) (Figure 7F–H and Figures S37 and S38, Supporting Information) and slightly decrease the accessibility of mesenchymal genes (Figure S39, Supporting Information). These changes correlated well with localized site‐specific epigenetic changes as ChIP‐qPCR analysis revealed significant increases in AcH3, H3K4me3 and H3K4me1 at the promoter regions of *Ascl1*, *Brn2*, *Myt1*, and *TUBB3* in PF573228‐treated cells relative to DMSO (Figure 7I). In addition, non‐transduced fibroblasts treated with the FAK inhibitor for 24 h displayed lower levels of DNA methylation, similar to blebbistatin treatment, as shown by a reduction in 5‐mC marks and DNMT activity (Figure 7J–L). These results demonstrate that a lower level of FAK enhances global and local epigenetic changes to promote iN reprogramming.

### 2.7 Biomaterial‐Mediated Reduction in Cell Adhesions Promotes iN Reprogramming

**Figure 8:**
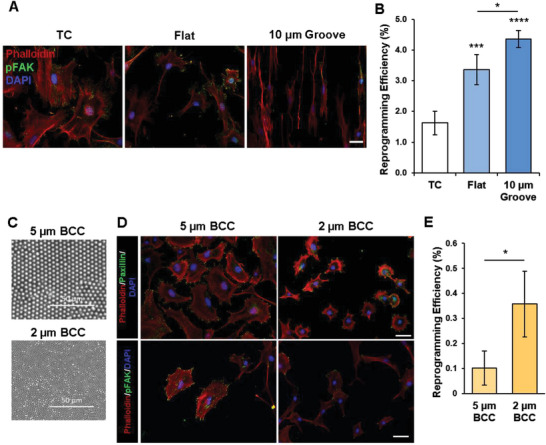
Reduction in cell adhesions using biomaterials promotes iN reprogramming. A) Immunofluorescent staining of actin network (phalloidin), phospho‐FAK, and nuclei (DAPI) in non‐transduced fibroblasts cultured in tissue culture‐treated wells (TC), flat PDMS membranes (Flat), and PDMS membranes with 10‐µm microgrooves. Scale bar, 50 µm. B) Reprogramming efficiency of BAM‐transduced fibroblasts cultured in tissue culture‐treated wells (TC), flat PDMS membranes (Flat), and PDMS membranes with 10 µm grooves at day 14 (*n* = 4). Significance was determined by a one‐way ANOVA and Tukey's multiple comparison test. C) Scanning electron microscopy images of 5 µm and 2 µm binary colloidal crystals (BCC). D) Immunofluorescent staining of actin network (phalloidin), paxillin, and nuclei (DAPI) (top 2 panels) or actin network (phalloidin), phospho‐FAK, and nuclei (DAPI) (bottom 2 panels) in non‐transduced fibroblasts cultured on 5 µm and 2 µm BCCs for 24 h. Scale bar, 50 µm. E) Reprogramming efficiency of BAM‐transduced fibroblasts cultured on 5 µm and 2 µm BCCs at day 14 (*n* = 3). Significance determined by two‐tailed, unpaired *t*‐test. Bar graphs show mean ± standard deviation (*p < 0.05, ***p < 0.001, ****p < 0.0001).

Given we had observed that reduction of intracellular tension and cell adhesion improved iN reprogramming, we postulated that modulating cell adhesion using biomaterials would produce a similar effect. When fibroblasts were grown on tissue‐culture (TC) polystyrene wells or polydimethylsiloxane (PDMS) membranes with a flat surface or 10‐µm microgrooves, we observed a decrease in stress fibers and phosphorylated FAK on PDMS membranes relative to TC wells, with the lowest levels on 10‐µm microgrooves (**Figure** **8**A). Consistently, the iN reprogramming efficiency correlated inversely with stress fibers and FAK phosphorylation (Figure 8B and Figure S40, Supporting Information). In another example, when fibroblasts were cultured on binary colloidal crystals (BCCs) composed of spherical particle materials with distinct silica microparticle sizes (i.e., 5 vs 2 µm), we observed less cell spreading, paxillin‐positive punctate and phosphorylated FAK expression in fibroblasts cultured on 2 µm BCCs, which coincided with an increase in the reprogramming efficiency (Figure 8C–E). Moreover, cells cultured on these BCCs had decreased cytoskeletal and nuclear stiffness, suggesting that these biomaterials could alter cell stiffness and intracellular tension (Figure S41, Supporting Information). These findings demonstrate the potential of engineering biomaterials to modulate cell adhesion and thus, intracellular tension to enhance iN reprogramming.

## 3 Discussion

Our findings demonstrate, for the first time, that a reduction of cytoskeletal tension to an optimal level by using small molecule compounds, FAK inhibition, and material engineering promotes a more open chromatin structure and enhances cell reprogramming. In particular, the reduction of cytoskeletal tension can suppress heterochromatin marks, and increase AcH3 and H3K4 methylation globally and at the promoter of neuronal genes (Figures 5 and 7), which in turn facilitates the reprogramming process (as summarized in Figure S42, Supporting Information). It is important to note that this reduction of tension is only required in the early phase of the reprogramming process (3–5 days, Figure 3 and Figure S11, Supporting Information), suggesting that modulation of the epigenetic state is critical for initiating the reprogramming process but not necessarily for the maturation of the neuronal phenotype. Interestingly, in comparison to blebbistatin, other actin and microtubule modulators such as cytochalasin D, jasplakinolide, and nocodazole have opposite effects, that is, decreasing euchromatin marks (e.g., AcH3; Figures S27 and S28, Supporting Information) and inhibiting iN conversion (Figure 3) while jasplakinolide enhances actin polymerization and nocodazole induces stress fibers, cytochalasin D inhibits actin polymerization (Figure S10, Supporting Information). Therefore, these findings are consistent with the notion that a reduction of intracellular tension to an optimal level without compromising essential cell functions can promote epigenetic changes and cell reprogramming.

Our results suggest that intracellular tension regulates the epigenetic state through nuclear enzyme activities such as the increase of nuclear HAT and H3K4 HMT activity and the decrease in nuclear activity of HDAC and H3K4 HDM (Figures 5 and 7). One potential mechanism is the translocation of these enzymes between the cytoplasm and nucleus. Indeed, cytoskeleton tension can affect nuclear transport.^[^ ^34^ ^]^ In addition, we show that the reduction of cytoskeletal tension causes the wrinkling and partial disassembly of nuclear lamina but does not induce a detachment of lamina‐associated domain (LAD) of chromatin from the nuclear lamina (Figure S43, Supporting Information). How these nuclear lamina changes regulate the transport of epigenetic enzyme activities remains to be determined. Furthermore, reducing intracellular tension suppresses the expression of mesenchymal genes in euchromatin (Figures 4 and 6), which may be attributed to both the decrease of accessibility at promoter and enhancer regions and the translocation of transcriptional factors.^[^ ^35^, ^36^, ^37^ ^]^ It is worth noting that mechanotransduction mechanisms in adherent cells may differ from cells being deformed in suspension. Cells in suspension have low‐to‐none intracellular tension, and transient squeezing has a direct physical effect on the nucleus such as the partial disruption of the nuclear lamina and LAD association, which causes a decrease in heterochromatin marks including H3K9me3 and DNA methylation.^[^ ^11^ ^]^

When cells experience biomaterials with different shapes or sizes at micro or nano levels, the cell shape, FA formation, and actin cytoskeleton assembly may be affected.^[^ ^12^, ^38^, ^39^, ^40^ ^]^ As a result, cytoskeletal tension, which is coupled to cell adhesions and ECM, may be reduced or enhanced. Since FAs can transmit forces outside‐in or inside‐out between the ECM and intracellular actin cytoskeleton, this represents an exciting opportunity to boost cell reprogramming by engineering the properties of cell adhesive substrates such as ligand density, stiffness, and micro/nano‐topography.^[^ ^38^, ^41^, ^42^, ^43^, ^44^, ^45^, ^46^ ^]^ Indeed, our results indicate that a reduction in cell spreading and FA signaling using micro/nano materials can facilitate the reprogramming process (Figure 8). Taken together, our findings provide a potential explanation for the mechanotransduction mechanism by which cytoskeletal tension and cell adhesion can modulate reprogramming, and a rational basis for the design of novel biomaterials with biophysical properties that can be altered to provide an optimal level of cell adhesion and cytoskeletal tension that is conducive to direct reprogramming.

## 4 Experimental Section

All experiments were performed in accordance with relevant guidelines and ethical regulations approved by the UCLA Institutional Biosafety Committee (BUA‐2016‐222).

### Fibroblast Isolation, Culture, and Reprogramming

Mice utilized in these studies were housed under specific pathogen‐free conditions and 12‐h light/12‐h dark cycles with a control of temperature (20–26 °C) and humidity (30–70%). All experiments, including breeding, maintenance, and euthanasia of animals, were performed in accordance with relevant guidelines and ethical regulations approved by the UCLA Institutional Animal Care and Use Committee (Protocol # ARC‐2016‐036 and ARC‐2016‐101).

Ear tissues from adult B57BL/6 mice were isolated, minced, and partially digested in Liberase (0.025 mg mL^−1^, Roche) for 45 min under constant agitation at 37 °C. Partially digested tissues were plated and fibroblasts were allowed to migrate out (passage 0). Isolated fibroblasts were expanded in MEF medium (DMEM + 10% FBS [Corning] and 1% penicillin/streptomycin [GIBCO]) and used at passage 2 for all experiments. Fibroblasts from Tau‐EGFP reporter mice (004779; The Jackson Laboratory) were isolated as described above.

After transduction, mouse fibroblasts were seeded onto multi‐well tissue culture‐treated polystyrene dishes (Falcon) coated with laminin (0.1 mg mL^−1^, Corning) at 4000 cells per cm^2^. 24 h after seeding, the medium was replaced to MEF medium containing Dox (2 µg mL^−1^, Sigma). The following day (i.e., day 1) the medium was changed to N3 medium (DMEM/F12 [GIBCO] + N2 supplement [Invitrogen] + B27 supplement [Invitrogen] + 1% penicillin/streptomycin [Gibco] + Dox [2 µg mL^−1^, Sigma]) and the cultures were maintained in this medium for the duration of the experiments. For Ascl1‐only reprogramming, N3 medium was further supplemented with BDNF (5 ng mL^−1^, R&D systems) and GDNF (5 ng mL^−1^, R&D systems) after day 7. For cytoskeletal and cell adhesion disruptions, blebbistatin (Millipore), Y‐27632 (20 µm; Cayman Chemical), nocodazole (0.3 µm; Sigma), cytochalasin D (1 µm; Sigma), jasplakinolide (0.05 µm; Cayman Chemical), and PF573228 (Sigma) were administered in N3 medium on day 1 and for the first 7 days of reprogramming (unless stated otherwise) and used at the indicated concentrations. Culture medium was replenished every 2 days during reprogramming to maintain the activity of the small molecules. After culturing for the desired length (14 days for BAM and 21 days for Ascl1 only), the iN cells were analyzed and the reprogramming efficiency was determined.

### Lentiviral Production and Cell Transduction

Dox‐inducible lentiviral vectors for Tet‐O‐FUW‐Ascl1, Tet‐O‐FUW‐Brn2, Tet‐O‐FUW‐Myt1l, Tet‐O‐FUW‐GFP, and FUW‐rtTA plasmids were used to transduce fibroblasts for ectopic expression of Ascl1, Brn2, Myt1l, GFP, and rtTA. Lentivirus was made using established calcium phosphate transfection methods. Viral particles were collected and concentrated using Lenti‐X Concentrator (Clontech) according to the manufacturer's protocol. Stable virus was aliquoted and stored at −80 °C. For viral transduction, fibroblasts were seeded and allowed to attach overnight before incubation with the virus and polybrene (8 µg mL^−1^, Sigma) for 24 h. After incubation, transduced cells were reseeded onto laminin‐coated tissue culture dishes.

### Immunofluorescent Staining and Quantification

For immunostaining, cells were fixed with 4% paraformaldehyde (Electron Microscopy Sciences), permeabilized with 0.5% Triton‐X‐100 (Sigma), and blocked with 5% donkey serum (Jackson Immunoresearch) in phosphate buffered saline (PBS). For actin‐cytoskeleton staining, samples were incubated with fluorescein isothiocyanate‐conjugated phalloidin (Invitrogen) for 1 h. Primary antibodies (refer to Table S1, Supporting Information) were incubated for 1 h at room temperature or overnight at 4 °C, followed by 1‐h incubation with Alexa 488 and/or Alexa 546‐labeled secondary antibodies (Molecular Probes). Nuclei were stained with 4,6‐diamino‐2‐phenylindole (DAPI) (Invitrogen).

2 to 3 weeks after the addition of Dox, cultures were fixed and immunostained for neuronal beta–tubulin III (TUBB3). iN cells were quantified using a Zeiss Axio Observer.D1 and identified based on displays of a typical neuronal morphology (defined as cells with a circular cell body containing a neurite that was at least three times the length of the cell body) and positive TUBB3 expression, as previously described.^[^ ^5^ ^]^ The reprogramming efficiency was determined by as the percentage of TUBB3^+^ iN cells in each condition normalized to the number of cells plated at 24 h post‐seeding. Epifluorescence images were collected using a Zeiss Axio Observer.D1, Zeiss Axio Observer.Z1, and ImageXpress Micro XLS System (Molecular Devices), whereas confocal images were acquired using a Zeiss LSM710 microscope and Leica SP8 Confocal Laser Scanning microscope.

Quantification of histone intensity per nuclei was performed using an ImageJ macro. DAPI‐stained nuclei were segmented using Gaussian blur, thresholding, watershed, and analyze particle functions to identify individual nuclei. This mask was applied to the corresponding stained fluorescence channel to quantify the average fluorescence intensity within each nucleus.

### Quantitative Deformability Cytometry

To perform q‐DC, standard soft lithography methods were used to fabricate microfluidic channels in PDMS. A mixture of 10:1 ratio of base to cross‐linker (Sylgard 184, Dow Corning) was poured onto a master wafer containing bifurcating channels.^[^ ^47^ ^]^ After curing, the PDMS device layer was bonded to a No. 1.5 glass coverslip (Thermo Fisher) using plasma treatment (Plasma Etch, Carson City, NV). Within 48 h of device fabrication, cell suspensions of 1 × 10^6^ cells mL^−1^ were driven through constrictions of 9 µm (width) × 10 µm (height) by applying 69 kPa of air pressure. The images of cells were captured during deformation through the constrictions using a CMOS camera with a capture rate of 1600 frames s^−1^ (Vision Research, Wayne, NJ) mounted on an inverted Axiovert microscope (Zeiss, Oberkochen, Germany) equipped with a 20×/0.4NA objective. To analyze the time‐dependent shape changes of individual cells during deformation, a custom MATLAB (MathWorks, Natick, MA) code (https://github.com/rowatlab) was used.^[^ ^47^ ^]^ To determine the mechanical stresses applied to individual cells, devices that had been calibrated with agarose particles of defined elastic modulus as previously described were used.^[^ ^48^ ^]^ Stress–strain curves were obtained for single cells and a power‐law rheology model was subsequently fitted to the data to yield measurements of elastic modulus, fluidity, and transit time.

### Atomic Force Microscopy

To analyze the mechanical property of cells during the direct reprogramming of fibroblasts into neurons, mechanical measurements of single cells were performed using AFM (Bruker BioscopeResolve, Bruker Corp., USA) with silicon tipless cantilevers (NPO‐10, Bruker Corp., USA), a high sensitive cantilever *k* = 0.06 N m^−1^, and sample Poisson's ratio of 0.499 at the UCLA Nano and Pico Characterization Facility. Fibroblasts were transduced with individual or different combinations of the transgenes and then the cell stiffness at various time points was measured during the reprogramming process (e.g., days 0, 1, and 3), wherein for each condition at least 30 cells were analyzed. During the measurements, cells were cultured on a glass bottom dish with pre‐warmed PBS and set on a temperature‐controlled stage at 37 °C. The force–distance curves were recorded and the elastic modulus of cells was calculated by NanoScope Analysis (Bruker Corp., USA) using the Hertz model as the Fit Model. Similar AFM measurements were also conducted on control samples of non‐transduced and GFP‐transduced fibroblasts as well as fibroblasts treated with small molecule inhibitors or cultured on different biomaterials

### Electrophysiology

For functional assessment of the iN cells, patch‐clamp electrophysiology analysis was performed. All experiments were conducted at room temperature (22–24 °C). All reagents were purchased from Sigma‐Aldrich unless otherwise specified. Whole‐cell recording was made from neurons using a patch clamp amplifier (MultiClamp 700B, Axon Instr.) under infrared differential interference contrast optics. Microelectrodes were made from borosilicate glass capillaries, with a resistance of 4–5 MW. For recording action potentials, cells were held at −70 mV in a voltage‐clamp mode. The intracellular solution for whole‐cell recording of EPSPs and action potentials contained (in mm) 140 potassium gluconate, 5 KCl, 10 HEPES, 0.2 EGTA, 2 MgCl_2_, 4 MgATP, 0.3 Na_2_GTP, and 10 Na_2_‐phosphocreatine, pH 7.2 (adjusted with KOH).

For recording spontaneous EPSCs (sEPSCs), cells were pre‐treated with the extracellular bath solution containing 50 µm picrotoxin (Tocris) to exclude an inhibitory synaptic activity and held at −70 mV in a voltage‐clamp mode with the intracellular solution containing (in mm) 130 CsMeSO4, 7 CsCl, 10 HEPES, 1 EGTA, 4 MgATP, 0.3 Na2GTP, and 10 Na2‐phosphocreatine, pH 7.3 (adjusted with CsOH). After recording basal sEPSC responses for 5 min, 10 µm CNQX (Tocris) and 100 µm D,L‐APV (Tocris) were co‐treated to test whether sEPSCs were mediated by activation of both AMPA‐ and NMDA‐type of glutamate receptors. For measuring spontaneous IPSC (sIPSCs), cells were pre‐treated with the bath solution containing 10 µm CNQX and 100 µm D,L‐APV and held at −70 mV with the intracellular solution containing (in mm) 137 CsCl, 10 HEPES, 1 EGTA, 4 MgATP, 0.3 Na_2_GTP, and 10 Na_2_‐phosphocreatine, pH 7.3 (adjusted with CsOH). 50 µm picrotoxin was then treated to test a dependency of sIPSCs on GABA receptors after acquiring basal sIPSC responses for 5 min. Series resistance (10–25 MΩ) and input resistance (≈200 MΩ using potassium‐based internal solution; 1–2 GΩ using Cs‐based internal solution) were monitored throughout the whole‐cell recording or compared before and after sEPSC/IPSC recordings.

Off‐line analyses of action potential properties (number, amplitude, half‐width) and the amplitude and frequency of sEPSC and sIPSC were performed by using a threshold event detection function of the Clampfit software (Molecular Devices). Visualization of analysis results and their statistical tests were performed by using GraphPad Prism 6.0 software.

### Small Interfering RNA Knockdown

RNA interference was performed using ON‐TARGETplus Non‐targeting Pool (Horizon Discovery, D‐001810‐10‐05), ON‐TARGETplus MYH9 siRNA (Horizon Discovery, L‐040013‐00‐0005), and ON‐TARGETplus MYH10 siRNA (Horizon Discovery, L‐062322‐00‐0005) and transfections were carried out using Lipofectamine 3000 (Invitrogen, L3000001) according to the manufacturer's protocol. Briefly, 7.5 µL of Lipofectamine was mixed with 250 µL of Opti‐MEM (GIBCO, 31985062) and incubated at room temperature for 5 min. Concurrently, 10 µL of siRNA (20 µm) was mixed with 250 µL of Opti‐MEM (Thermo Fisher, 31985062). These two solutions were mixed and incubated at room temperature for 20 min and the siRNA‐lipid complexes were added to 1.5 mL DMEM. Cells were incubated at 37 °C for 7 h and then the medium was replaced with culture medium. Samples were collected 24 h after transfection for qPCR analysis and 48 h after transfection for protein expression analysis to confirm knockdown.

### Quantitative Reverse Transcriptase‐Polymerase Chain Reaction

RNA was isolated from samples using Trizol (Ambion) according to the manufacturer's instructions. For cDNA synthesis, 500 ng of RNA was reverse transcribed using Maxima First Strand cDNA Synthesis Kit (Thermo Fisher Scientific). Template DNA was amplified using Maxima SYBR Green/Fluorescein qPCR Master Mix (Thermo Fisher Scientific) on a CFX qPCR machine (Bio‐Rad). qRT‐PCR data were analyzed using CFX Manager 3.1 (Bio‐Rad) and gene expression levels were normalized to 18S. Primers used for qRT‐PCR are included in Table S2, Supporting Information.

### Chromatin Immunoprecipitation‐qPCR

Halt Protease and Phosphatase Inhibitor Cocktail (Thermo Fisher Scientific, 78442) was added to cell lysis (10 mm Tris‐HCl pH 8.0, 85 mm KCl, 0.5% NP‐40), nuclei lysis (10 mm Tris‐HCl pH 7.5, 1% NP‐40, 0.5% sodium deoxycholate, 0.1% SDS), and ChIP dilution (0.01% SDS, 1.1% Triton X‐100, 1.2 mm EDTA, 16.7 mm Tris‐HCl pH 8.1, 167 mm NaCl) buffers.

3 days post‐Dox addition, 4 × 10^6^ BAM‐transduced fibroblasts cultured in the absence and presence of 10 µm blebbistatin and 5 µm PF573228, respectively, for 2 days were fixed using 1% formaldehyde in PBS (Fisher Scientific, BP531) for 10 min. 125 mm glycine was added for 5 min to quench excess formaldehyde, followed by 2 washes with cold 1× PBS. Cells were scraped and collected into microcentrifuge tubes and centrifuged at 800 × *g* at 4 °C for 5 min. Upon removing the supernatant, cell pellets were snap‐frozen in liquid nitrogen and stored at −80 °C. The cells were then resuspended and lysed in cell lysis buffer and resuspended in nuclei lysis buffer prior to sonication using a Branson SFX250 Sonifier at 40% amplitude, 0.7 s on, and 1.3 s off, for a total of 8 min. Samples were spun down at maximum speed in a 4 °C centrifuge and the supernatant was collected. 50 µL was removed from each sample and stored at 4 °C as a downstream internal control.

1.5 µg of normal rabbit IgG (Millipore, CS200581), anti‐rabbit H3K4me3 antibody (Millipore, 04‐473), anti‐rabbit Histone 3 Acetylation (Millipore, 06‐599), or anti‐rabbit H3K4me1 antibody (Abcam, ab8895) were added to samples and incubated in a rotator overnight at 25 rpm in a 4 °C refrigerator. 20 µL of Pierce protein A/G magnetic agarose beads (Thermo Fisher Scientific, 78610) were washed with Chip dilution buffer using a magnetic separation rack and added to each sample and incubated in a rotator for 2 h at 25 rpm in a 4 °C refrigerator.

The supernatant was removed from the beads using a magnetic separation rack and the beads were subjected to a series of wash buffers: Low salt immune complex wash buffer (0.1% SDS, 1% Triton X‐100, 2 mm EDTA, 20 mm Tris‐HCl pH 8.1, 150 mm NaCl), high salt wash buffer (0.1% SDS, 1% Triton X‐100, 2 mm EDTA, 20 mm Tris‐HCl pH 8.1, 150 mm NaCl), LiCl immune complex wash buffer (0.25 m LiCl, 1% NP40, 1% sodium deoxycholate, 1 mm EDTA, 10 mm Tris‐HCl pH 8.1), and Tris‐EDTA (10 mm Tris‐HCl pH 8.0, 1 mm EDTA). The beads were resuspended in 50 µL of freshly prepared ChIP Elution Buffer (1% SDS, 0.1 m NaHCO_3_) and placed in a 65 °C bath for 10 min. The supernatant was collected and this elution step was performed once more and the corresponding eluates were combined.

50 µL ChIP elution buffer was added to the stored internal controls from the post‐sonication step. 20 µL of reverse cross‐linking salt mixture (250 mm Tris‐HCl pH 6.5, 62.5 mm EDTA pH 8.0, 1.25 m NaCl) with 5 mg mL^−1^ proteinase K (Life Technologies, AM2548) and 62.5 ng µL^−1^ RNase A (AG Scientific, R‐2000) was added to each sample and internal control and incubated at 65 °C overnight. Samples were purified using AMPure XP beads (Beckman Coulter Life Sciences, A63881) at 2× volume according to the manufacturer's instructions. qRT‐PCR was performed on input samples, ChIP DNA samples, and control samples using the primers listed in Table S3, Supporting Information, and a CFX qPCR machine (Bio‐Rad). Substantial fold enrichment was observed for each experimental condition (Figure S44, Supporting Information). ChIP‐qPCR data were analyzed by normalizing the DNA concentration to percent input using the relative standard curve method.

### RNA Sequencing

RNA was isolated from non‐transduced and Ascl1‐transduced fibroblasts at day 3 using Trizol (Ambion) according to the manufacturer's protocol. A total of 500 ng total RNA was subjected to poly A selection using the Dynabeads mRNA DIRECT kit (Invitrogen) followed by library preparation using the PrepX RNA‐Seq for Illumina Library Kit (Wafergen) before sequencing on the HiSeq4000 (Illumina) at 50 single‐read runs. Fastqc files were trimmed with trim galore v0.6.4 using default settings. Trimmed fastQ files were aligned to GRCm38 reference genome using STAR v2.7.1a^[^ ^49^ ^]^ with default parameters and with “quantMode GeneCounts” enabled to obtain the number of reads per gene. Gene counts were imported into R and differentially expressed genes were identified with DESEq2 v1.20.0^[^ ^50^ ^]^ after fitting a linear model to account for the experimental variables. GO analysis was performed on the differentially expressed genes using the GOseq v1.32.0^[^ ^51^ ^]^ package.

### 10x Single‐Cell RNA Sequencing: Single Cell Preparation and Transcriptome Profiling

For single‐cell RNA sequencing analysis, 1 × 10^5^ BAM‐transduced fibroblasts were cultured in the absence or presence of 10 µm blebbistatin for 48 h and collected on day 3. Cells were trypsinized and single cells were resuspended in the appropriate buffer and introduced into 10x Chromium for single cell 3’ transcriptome profiling. Briefly, single cells with specific 10x Barcode and unique molecular identifier were generated by partitioning the cells into Gel Bead‐In‐Emulsions. Subsequent cDNA sequences with the same 10x Barcode were considered as sequences from 1 cell. The library generated by 10x Chromium machine was then sequenced on a Novaseq S1.

### 10x Single‐Cell RNA Sequencing: scRNA‐Seq Data Preprocessing

FASTQ files were processed with 10x Genomics’ Cell Ranger analysis pipelines. The read count matrix generated by CellRanger was then analyzed using Seurat V4.^[^ ^52^ ^]^ 33 285 genes were detected across DMSO‐ (10 079 and 11 528 cells tested for replicates) and blebbistatin‐treated samples (7503 and 9557 cells tested for replicates). Cells that had unique feature counts with at least 200 genes but no more than 7000 genes and cells that had <5% mitochondrial counts were filtered and normalized based on the feature expression and total expression of each cell. The normalized expression data were then used for subsequent analysis.

### 10x Single‐Cell RNA Sequencing: Dimensional Reduction, Clustering, Differential Gene Expression Analysis, and Single Cell Trajectory Analysis

Principal component analysis was performed using highly variable genes in each sample after linear transformation and the first 10 PC scores were used for UMAP analysis to cluster the cells into 12 groups (FindNeighbors and FindClusters functions implemented in the Seurat package, dims = 10, resolution = 0.6). The marker genes of each cluster were identified using FindAllMarkers function with default parameters. Clusters were annotated using canonical genes in the neural reprogramming process and published datasets as reference,^[^ ^53^ ^]^ where nine clusters were finally identified to represent different phases of reprogramming. The percentage of cells expressing neuronal genes including *MAPT*, *TUBB3*, *STMN3*, and *SNAP25* were examined to show evidence for neuronal reprogramming. Single‐cell trajectory analysis was performed using Monocle3.^[^ ^54^, ^55^ ^]^ Clusters identified by Seurat were used for cell ordering, and trajectory visualization using default parameters in Monocle3 analysis.

### 10x Single‐Cell RNA Sequencing: Pseudobulk Differential Expression Analysis

A pseudobulk method was applied to investigate differential gene expression among DMSO‐ and blebbistatin‐treated samples at the population level. Specifically, the raw counts of each sample were extracted after QC filtering. The counts were then aggregated to the sample level to be used for the differential expression analysis using DESeq2.^[^ ^50^ ^]^ Genes that were up‐ or down‐regulated in blebbistatin‐treated conditions compared to DMSO‐treated conditions were defined with *p*_adj_ = 0.05 as the threshold. GO enrichment analysis was then performed toward up‐ and down‐regulated genes using the enrichGO function in clusterProfiler package.^[^ ^56^ ^]^

### Assay of Transposase Accessible Chromatin Sequencing: Cell Preparation, Transposition Reaction, ATAC‐Seq Library Construction, and Sequencing

A total of 1 000 000 fibroblasts treated with vehicle control (DMSO), 10 µm blebbistatin, or 5 µm PF573228 were collected after 2 h and stored at −80 °C prior to sample processing. ATAC‐seq was performed as described previously.^[^ ^57^ ^]^ In brief, frozen cells were thawed and washed once with PBS and then resuspended in 500 µL of cold PBS. The cell number was assessed by Cellometer Auto 2000 (Nexcelom Bioscience, Massachusetts, USA). 100 000 cells were then added to ATAC lysis buffer and centrifuged at 500 × *g* in a pre‐chilled centrifuge for 5 min. Supernatant was removed and the nuclei were resuspended in 50 µL of tagmentation reaction mix by pipetting up and down. The reactions were incubated at 37 °C for 30 min in a thermomixer with shaking at 1000 rpm, and then cleaned up using the MiniElute reaction clean up kit (Qiagen). Tagmented DNA was amplified with barcoded primers. Library quality and quantity were assessed with Qubit 2.0 DNA HS Assay (Thermo Fisher), Tapestation High Sensitivity D1000 Assay (Agilent Technologies), and QuantStudio 5 System (Applied Biosystems). Equimolar pooling of libraries was performed based on QC values and sequenced on an Illumina NovaSeq (Illumina, California, USA) with a read length configuration of 150 PE for [100]M PE reads (50 m in each direction) per sample.

### Assay of Transposase Accessible Chromatin Sequencing: Mapping, Peak Calling, and Differential Peak Analysis

FASTQ files were trimmed with Trim Galore and Cutadapt.^[^ ^58^ ^]^ Pair‐ended reads were then aligned to the mouse reference genome (mm10) with Bowtie2.^[^ ^59^ ^]^ Mitochondrial reads and PCR duplicates were removed using SAMtools^[^ ^60^ ^]^ and Picard (http://broadinstitute.github.io/picard/), respectively. Peaks were called over input using MACS3,^[^ ^61^ ^]^ and only peaks outside the ENCODE blacklist region were kept. All peaks from all samples were combined and merged using bedtools and featureCount^[^ ^62^ ^]^ was used to count the mapped reads for each sample. Peaks that were up‐ or down‐regulated under different conditions were defined by using DESeq2^[^ ^50^ ^]^ with *p*_adj_ = 0.001 as the threshold. Peaks located at *cis*‐regulatory elements related to genes of interest (±5 kb region) were visualized using Integrative Genomics Viewer (IGV)^[^ ^63^ ^]^ to demonstrate differentially up‐ or down‐regulated differential peaks. To identify differentially accessible regions around Ascl1‐targeting genes, an Ascl1 ChIP‐seq dataset that was previously published^[^ ^27^ ^]^ (GSE43916, Ascl1 MEF) was utilized. Genome coordinates of peaks were converted from mm9 to mm10 reference genome by using UCSC liftOver tools. ATAC peaks around Ascl1‐target genes were visualized using IGV.

### Western Blotting

Fibroblasts were lysed and collected in Laemmli buffer (0.0625 mm Tris‐HCl, 10% glycerol, 2% SDS, 5% 2‐mercaptoethanol, 0.002% bromophenol blue) containing RIPA buffer (50 mm Tris‐HCl, 150 mm NaCl, 1% Triton‐X‐100, 0.1% SDS, 10 mm NaF, 0.5% sodium deoxycholate) along with protease inhibitors (PMSF, Na_3_VO_4_, and Leupeptin) on ice. Protein lysates were centrifuged to pellet cell debris, and the supernatant was collected and used in further analysis. Protein samples were run using SDS‐PAGE and transferred to polyvinylidene fluoride membranes. Membranes were blocked in 3% nonfat milk and incubated with primary antibodies overnight. Primary antibodies include pFAK, FAK, *α*SMA, Calponin, pERK, ERK, and GAPDH. Refer to Table S1, Supporting Information, for all antibody information. Membranes were washed with Tris‐Buffered Saline + 0.05% Tween‐20 and incubated with HRP‐conjugated IgG secondary antibodies (Santa Cruz Biotechnologies) for 1 h. Protein bands were visualized using Western Lightning Plus—Enhanced Chemiluminscence Substrate (Perkin Elmer Life & Analytical Sciences) and imaged on a ChemiDoc XRS system (Bio‐Rad).

### Histone Acetyltransferase, Histone Deacetylase, H3K4 Histone Methyltransferase, H3K4 Histone Demethylase, and DNA Methyltransferase Activity Assays

Nuclear protein extractions were isolated from 5 × 10^5^ fibroblasts treated with vehicle control (DMSO), 10 µm blebbistatin, or 5 µm PF573228 for 2 h using a nuclear extraction kit (EpiGentek, OP‐0002), in accordance with the manufacturer's instructions. HAT, HDAC, H3K4 HMT, H3K4 HDM, and DNMT activity were measured using the HAT activity/inhibition assay (EpiGentek, P‐4003‐048), HDAC activity/inhibition assay (EpiGentek, P‐4034‐096), HMT (H3K4 specific) activity/inhibition assay (EpiGentek, P‐3002‐1), HDM (H3K4 specific) activity/inhibition assay (EpiGentek, P‐3074‐48), and DNMT activity/inhibition assay (EpiGentek, P‐3009‐048), respectively. Per the manufacturer's instructions, 20 µg of nuclear extract was added into the assay wells and incubated at 37 °C for 90 min. After adding the color developer solution, the absorbance was measured using a plate reader (Infinite 200Pro, 30050303) at 450 nm for all the assays with the exception of the HDM (H3K4 specific) activity/inhibition assay where the fluorescence was measured using a fluorescence microplate reader at 530 EX/590 EM nm.

### DNA Methylation Assay

DNA was isolated from non‐transduced fibroblasts treated with vehicle control (DMSO), 10 µm blebbistatin, and 5 µm PF573228, respectively, for 24 h using the PureLink Genomic DNA mini kit (Invitrogen) according to the manufacturer's instructions. To detect global DNA methylation (5‐mC) levels in the samples, the MethylFlash Global DNA Methylation (5‐mC) ELISA Easy kit (Epigentek) was utilized according to the manufacturer's protocol. 100 ng of DNA sample was utilized per reaction and after adding the color developer solution, the absorbance was measured using a plate reader (Infinite 200Pro, 30050303) at 450 nm.

### Microgroove Substrate Fabrication

Bioengineered substrates were fabricated as previously described.^[^ ^38^ ^]^ Briefly, PDMS membranes were fabricated using well established soft lithography procedures and sterilized using 70% ethanol for 10 min. PDMS membranes were plasma treated for 1 min and coated with laminin (0.1 mg mL^−1^, Corning) overnight to promote cell attachment. Fibroblasts were seeded onto PDMS membranes at 4000 cells per cm^2^ for subsequent experiments.

### Binary Colloidal Crystal Fabrication

BCC monolayers were fabricated as previously described.^[^ ^64^, ^65^ ^]^ Briefly, BCCs were fabricated by mixing two colloids and depositing them into 24 well plates. The number of particles was determined based on the equation in which the surface could be fully covered. BCCs were heated to stabilize the particle layers. Prior to cell culture, BCCs were sterilized with UV light in biosafety hood and plasma treated for 2 min. BCCs were coated with 0.1 mg mL^−1^ laminin for 1 h prior to cell seeding. BCCs were characterized using scanning electron microscopy (ZEISS SUPRA 40 VP, Carl Zeiss, Germany), where BCCs were coated with 5 nm gold before imaging.

### Statistical Analysis

The data were presented as mean plus or minus one standard deviation, where *n* ≥ 3. The data corresponding to the q‐DC, AFM, and histone quantification experiments were displayed as box‐and‐whisker plots. The boxes were drawn with the ends at the quartiles, the median as a horizontal line in the box, the mean as a (+) symbol, and the whiskers extend from the minimum to maximum data point. Comparisons among values for groups greater than two were performed using a one‐way or two‐way analysis of variance (ANOVA) and differences between groups were determined using the following multiple comparison tests: Dunnett's, Tukey's, and Sidak's post‐hoc test. For comparison between two groups, a two‐tailed, unpaired *t*‐test was used. For all cases, *p*‐values less than 0.05 were considered statistically significant. GraphPad Prism 6.0 and GraphPad Prism 8.0 software were used for all statistical analysis.

## Conflict of Interest

The authors declare no conflict of interest.

## Author Contributions

Je.S. and S.L. designed the experiments. Je.S., Y.S., B.C., H.P., N.A., P.‐Y.W., C.L., S.W., D.O.K., and J.C. performed the experiments. Je.S., Y.S., Y.W., B.C., H.P., P.‐Y.W., T.H., C.L., Ju.S., S.W., and D.O.K. analyzed the data. Je.S., M.‐M.P., T.‐L.D., A.C.R., and S.L. contributed to data interpretation and discussion. Je.S. and S.L. wrote the manuscript.

## Supporting information

Supporting Information

## Data Availability

The authors declare that all data supporting the findings of this study are available within the paper and supplementary information files.
