# Supplementary material for: Reduction of Intracellular Tension and Cell Adhesion Promotes Open Chromatin Structure and Enhances Cell Reprogramming
Source: Adv Sci (Weinh). 2023 Jun 26;10(24):2300152. doi: 10.1002/advs.202300152 (PMC10460843; doi:10.1002/advs.202300152)
Supplement: Supplementary file 1 — Supporting Information [file ADVS-10-2300152-s001.pdf]

ADVANCED SCIENCE
Open Access

Supporting Information

for *Adv. Sci.*, DOI 10.1002/advs.202300152

Reduction of Intracellular Tension and Cell Adhesion Promotes Open Chromatin Structure and Enhances Cell Reprogramming

*Jennifer Soto, Yang Song, Yifan Wu, Binru Chen, Hyungju Park, Navied Akhtar, Peng-Yuan Wang, Tyler Hoffman, Chau Ly, Junren Sia, SzeYue Wong, Douglas O. Kelkhoff, Julia Chu, Mu-Ming Poo, Timothy L. Downing, Amy C. Rowat and Song Li**

Supporting Information

Reduction of Intracellular Tension and Cell Adhesion Promotes Open Chromatin Structure and Enhances Cell Reprogramming

*Jennifer Soto, Yang Song, Yifan Wu, Binru Chen, Hyungju Park, Navied Akhtar, Tyler Hoffman, Chau Ly, Junren Sia, SzeYue Wong, Douglas O. Kelkhoff, Julia Chu, Mu-Ming Poo, Timothy L. Downing, Amy Rowat, Song Li**

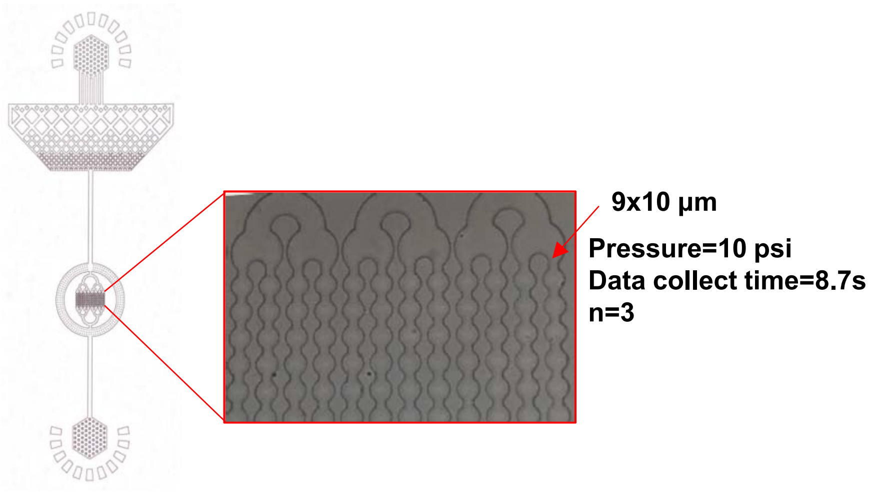

Figure S1. Stiffness was determined using quantitative deformability cytometry (q-DC). Image of the microfluidic device that enables single cells to transit through micro-constrictions (9x10 µm, 10 psi).

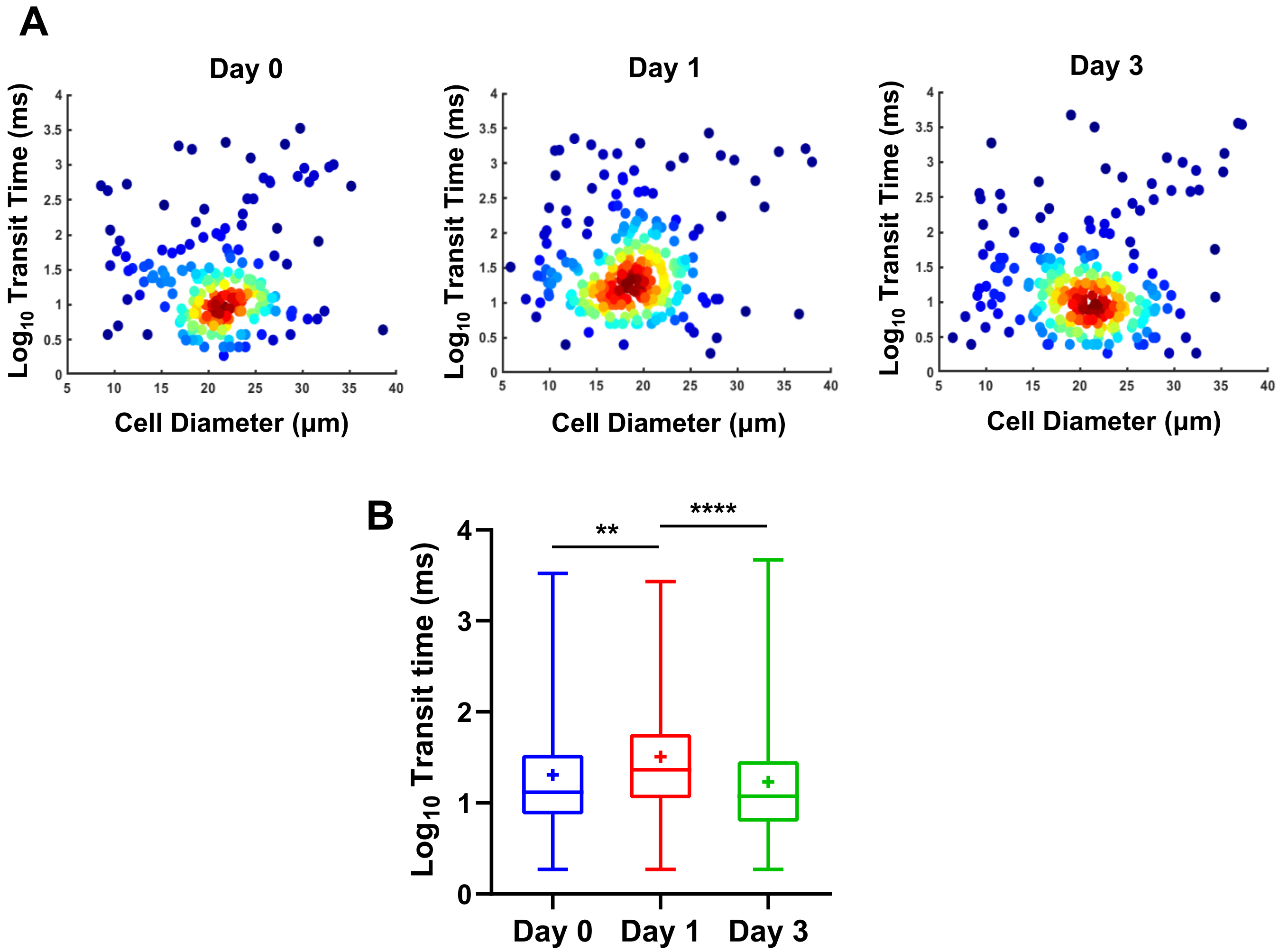

Figure S2. Cell mechanical phenotype was modulated during iN reprogramming as determined by quantitative deformability cytometry (q-DC). A, Transit time of BAM-transduced fibroblasts at the indicated time points (day 0, n= 211; day 1 n= 257; day 3, n= 253) as derived by q-DC. **B,** Density scatter plots show the log of transit time as a function of cell diameter for BAM-transduced fibroblasts deforming through 9 x 10 µm constrictions at the indicated time points (day 0, n= 211; day 1= 257; day 3, n= 253). Dots represent single-cell data. Box plots show the ends at the quartiles, the median as a horizontal line in the box, the mean as a (+) symbol, and the whiskers extend from the minimum to maximum data point. Significance determined by one-way ANOVA using Tukey's correction for multiple comparisons (**$p < 0.01$, ****$p < 0.0001$).

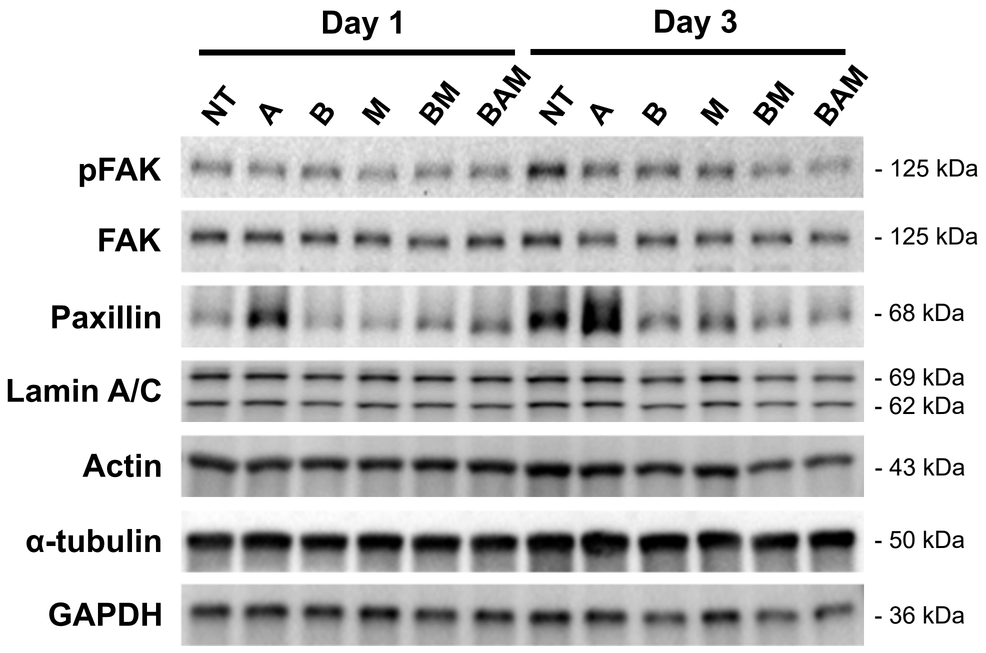

Figure S3. Effect of transgenes on cell adhesion and cytoskeletal proteins. Fibroblasts were either non-transduced or transduced with individual or a combination of transgenes, followed by Western blot analysis of focal adhesion, nuclear membrane and cytoskeletal proteins at the indicated time points. GAPDH serves as a control. NT, non-transduced; A, Ascl1; B, Brn2; M, Myt1l.

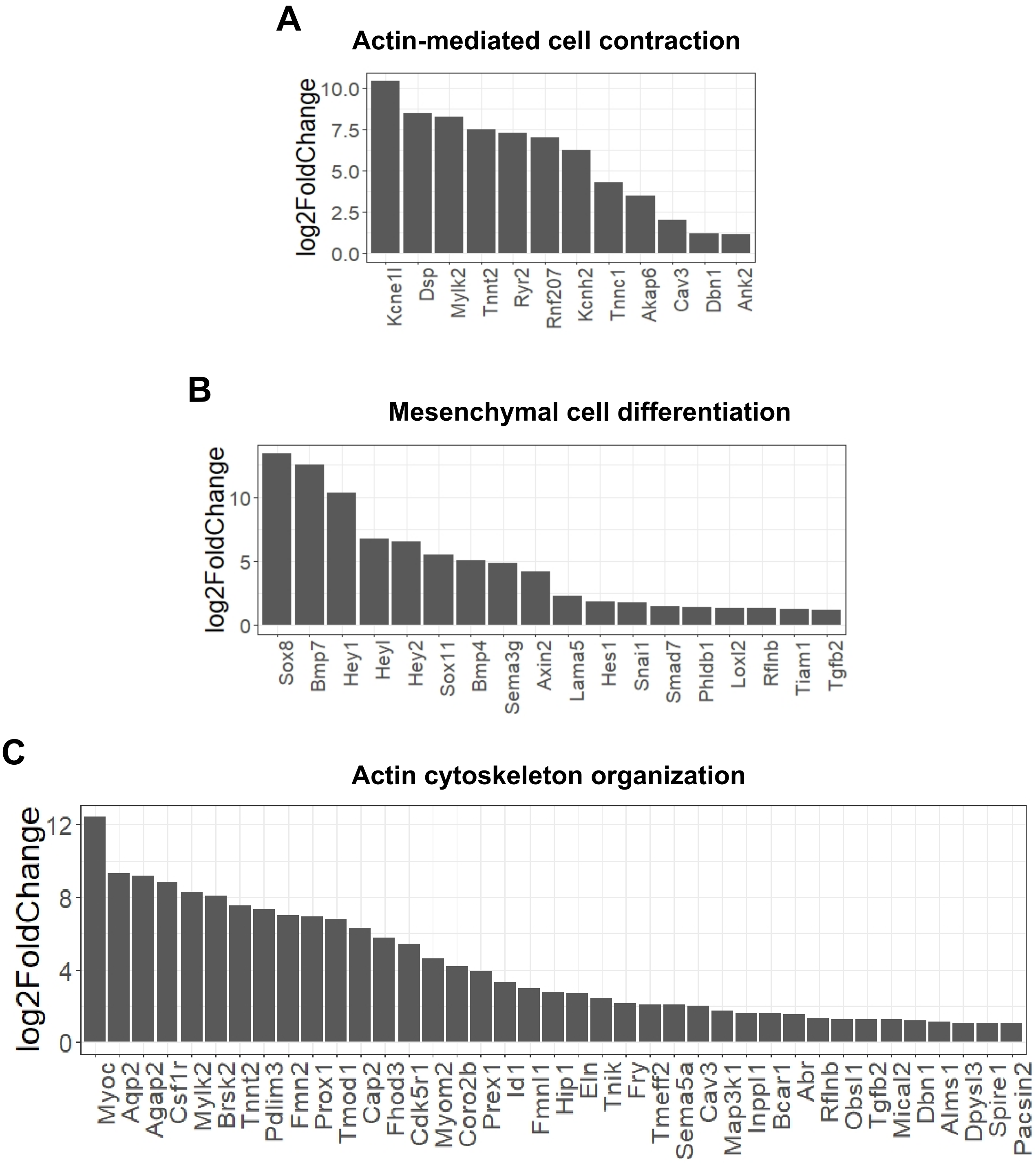

Figure S4. Ascl1 overexpression induces the upregulation of cytoskeletal- and mesenchymal-related genes. A-C, Bar plots show upregulated genes in each gene ontology category in Ascl1-transduced fibroblasts compared to non-transduced cells, as determined by RNAseq analysis.

Cell adhesion

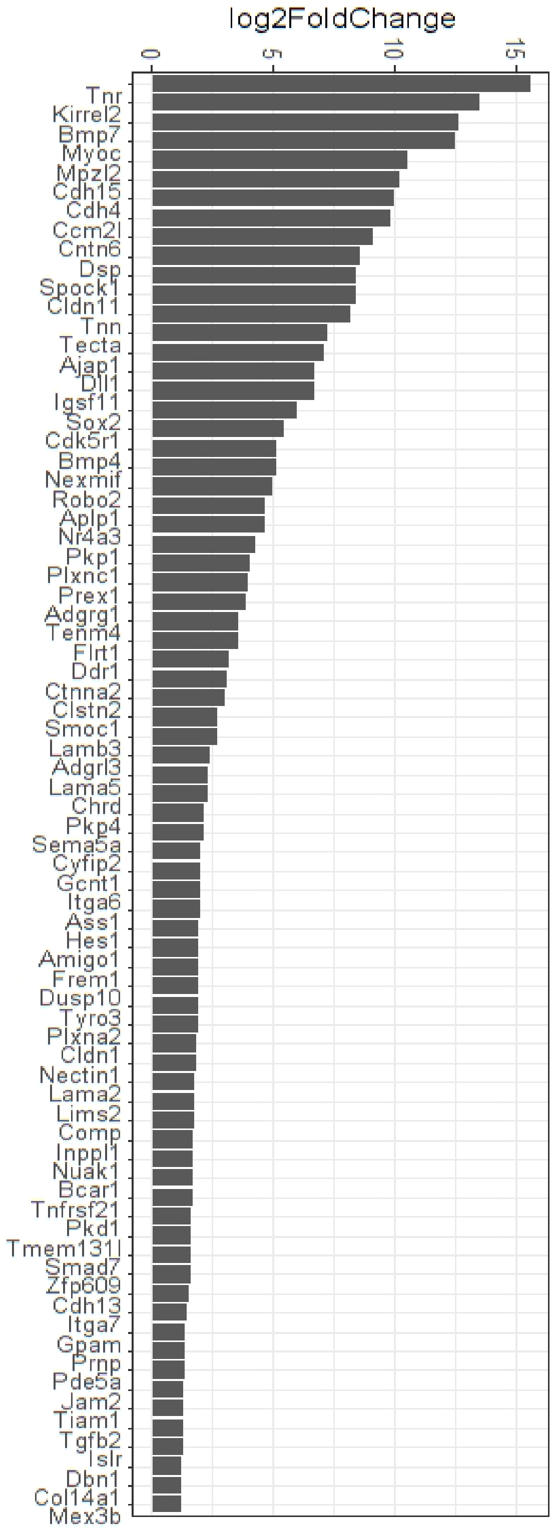

Figure S5. Ascl1 overexpression induces the upregulation of cell adhesion-related genes. Bar plot shows upregulated genes in each gene ontology category in Ascl1-transduced fibroblasts compared to non-transduced cells, as determined by RNAseq analysis.

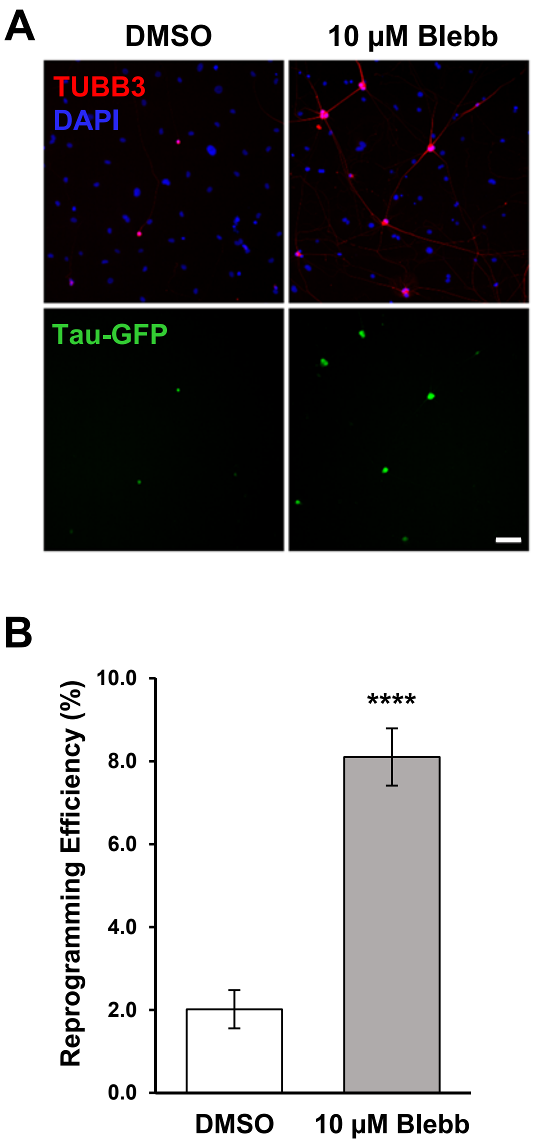

Figure S6. Cytoskeletal disruption promotes the iN conversion of Tau-eGFP fibroblasts. A, Immunofluorescent images show that iN cells derived from Tau-EGFP fibroblasts cultured in the presence of blebbistatation express β-tubulin III (TUBB3). Scale bar, 100 μm. **B,** Reprogramming efficiency of Tau-EGFP fibroblasts transduced with BAM and cultured in the presence of 10 μM blebbistatin at day 14 (n=4). Significance determined by two-tailed, unpaired student's *t* test. Bar graphs show mean ± standard deviation (****p < 0.0001).

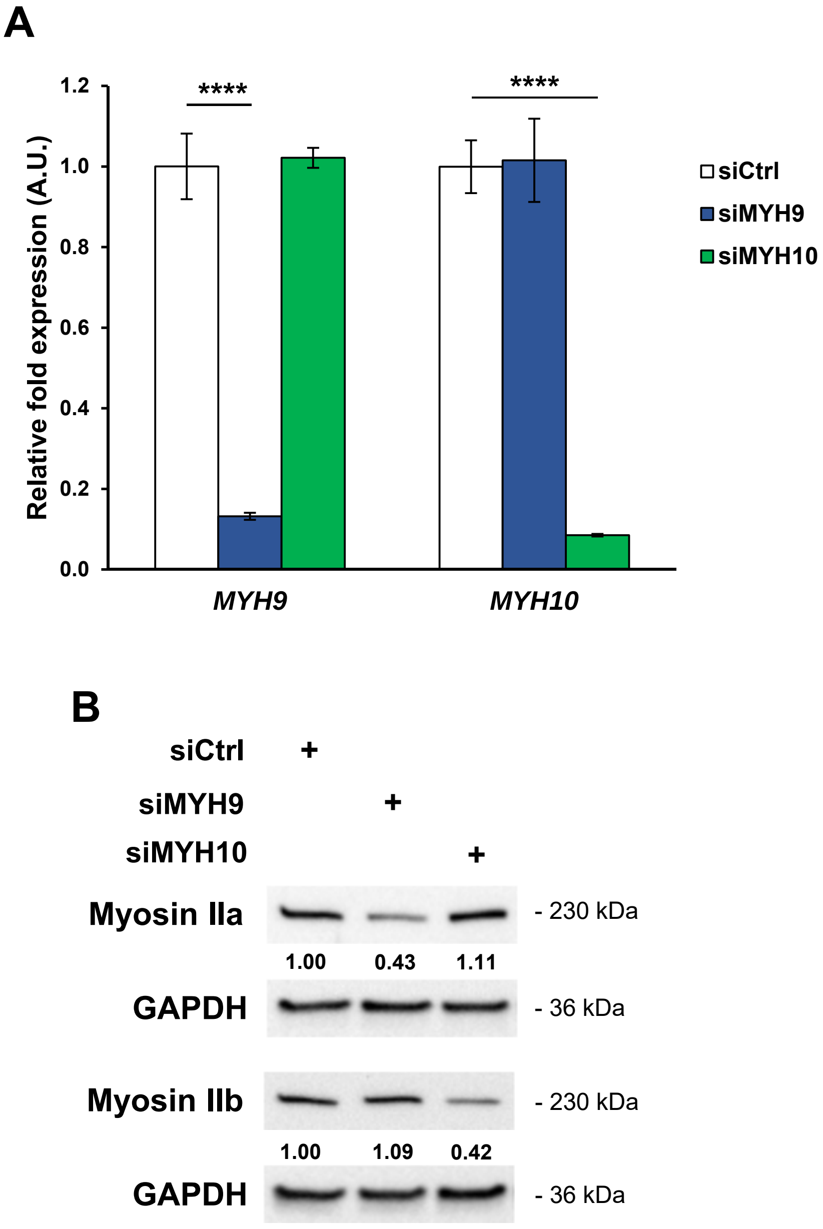

Figure S7. Myosin II knockdown by using siRNA interference. A, Fibroblasts were transfected with an siRNA against myosin II (siMYH9 and siMYH10) or negative control (siCtrl), respectively, followed by qRT-PCR analysis of MYH9 and MYH10 gene expression at day 1 (n=3). The qRT-PCR analysis confirmed the successful knockdown of myosin II gene expression, where gene expression was normalized to 18S RNA levels. Significance determined by one-way ANOVA and Dunnett's multiple comparison test. Bar graph represents mean ± one standard deviation (****p < 0.0001). **B,** Western blot analysis of myosin II expression in fibroblasts transfected with an siRNA against myosin II (siMYH9 and siMYH10) or negative control (siCtrl), respectively, at 48 hours after siRNA knockdown.

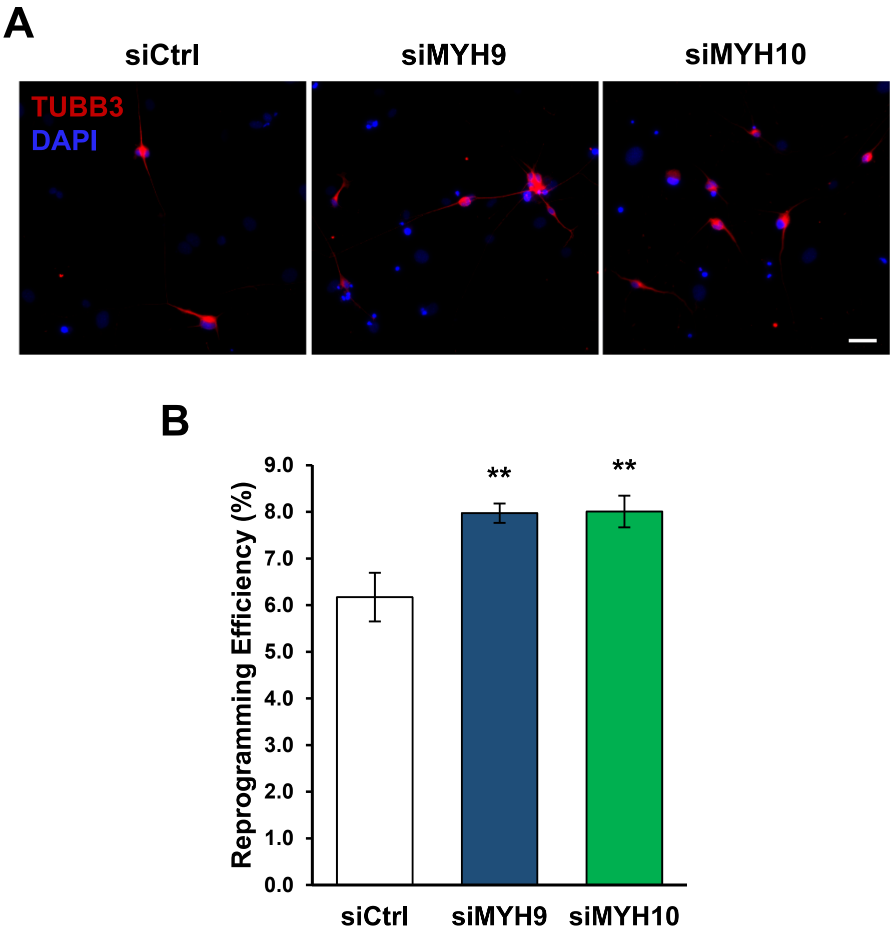

Figure S8. Myosin II knockdown promotes iN reprogramming. A, Immunofluorescent images of TUBB3$^+$ iN cells derived from BAM-transduced fibroblasts transfected with an siRNA against myosin II (siMYH9 and siMYH10) or negative control (siCtrl), respectively, at day 10. Scale bar, 50 µm. **B**, Reprogramming efficiency of BAM-transduced fibroblasts transfected with an siRNA against *MYH9* and *MYH10* at day 10 (n=3). Significance determined by two-way ANOVA using Dunnett's multiple comparison test. Bar graph shows mean ± standard deviation (**$p < 0.01$).

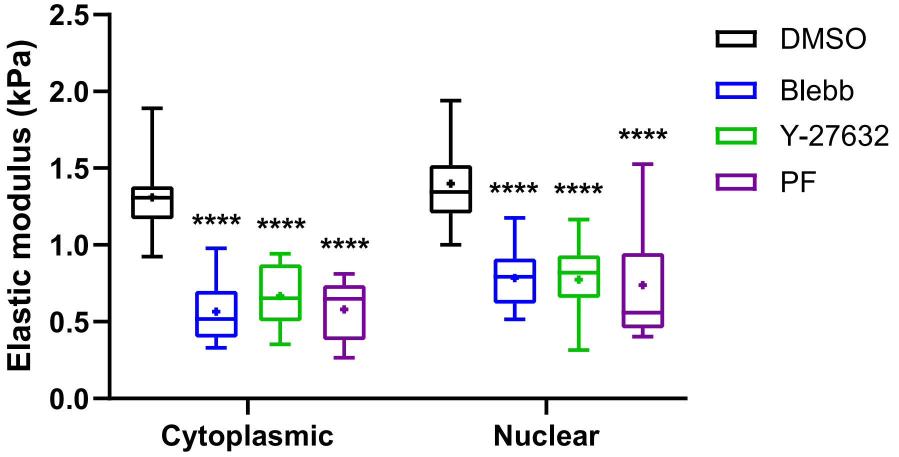

Figure S9. Effect of cytoskeletal and cell adhesion disruption on cell stiffness. Box plots illustrate the variation in elastic modulus of non-transduced fibroblasts cultured in presence of vehicle control (DMSO), 10 μM blebbistatin, 20 μM Y-27632, and 5 μM PF573228 for 2 hours, as acquired using AFM (DMSO, n= 35 cells; Blebb, n= 34 cells; Y-27632, n= 33 cells; PF573228, n= 30 cells). Significance determined by two-way ANOVA using Dunnett's correction for multiple comparisons. Box plots show the ends at the quartiles, the median as a horizontal line in the box, the mean as a (+) symbol, and the whiskers extend from the minimum to maximum data point (****$p < 0.0001$).

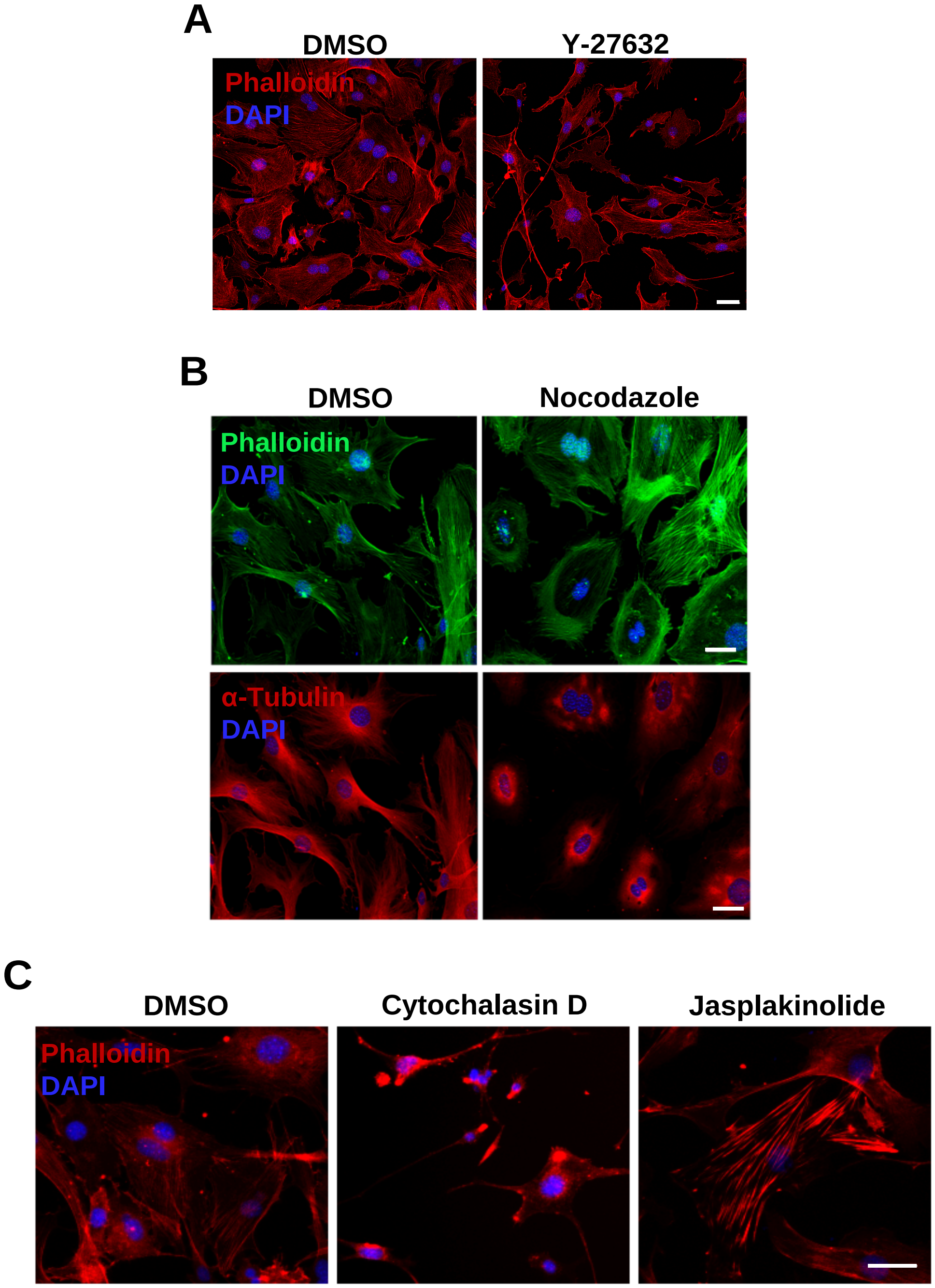

Figure S10. Effect of cell contractility, microtubule and actin polymerization inhibition on the cytoskeleton. Immunofluorescent images show the actin network (phalloidin, red) and nucleus (DAPI, blue) of non-transduced fibroblasts treated with or without 20 µM Y-27632 (**A**), 0.3 µM Nocodazole (**B**), 1 µM Cytochalasin D (**C**), or 0.05 µM Jasplakinolide (**C**) for 2 or 24 hours (**A**, **B**). Scale bar, 50 µm (**A-B**) or 25 µm (**C**).

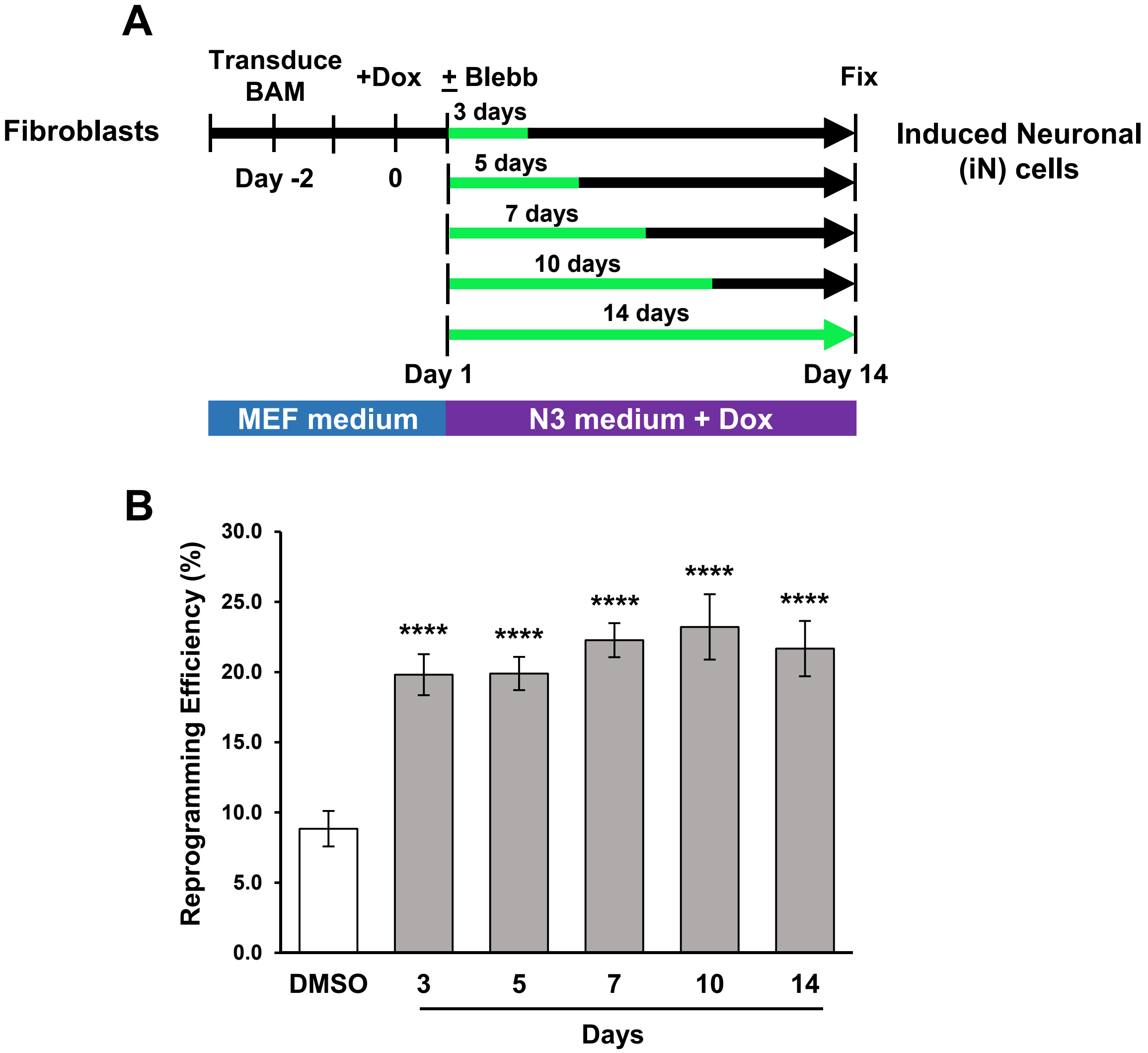

Figure S11. The effects of cytoskeletal disruption are critical during the early phase of iN reprogramming. A, Schematic illustrating the length of time blebbistatin was administered during the reprogramming process. **B,** Reprogramming efficiency at day 14 of BAM-transduced fibroblasts treated with 10 μM blebbistatin for the indicated number of days (n=4). Significance determined by one-way ANOVA using Dunnett's multiple comparison test. Bar graph shows mean ± standard deviation (****$p < 0.0001$).

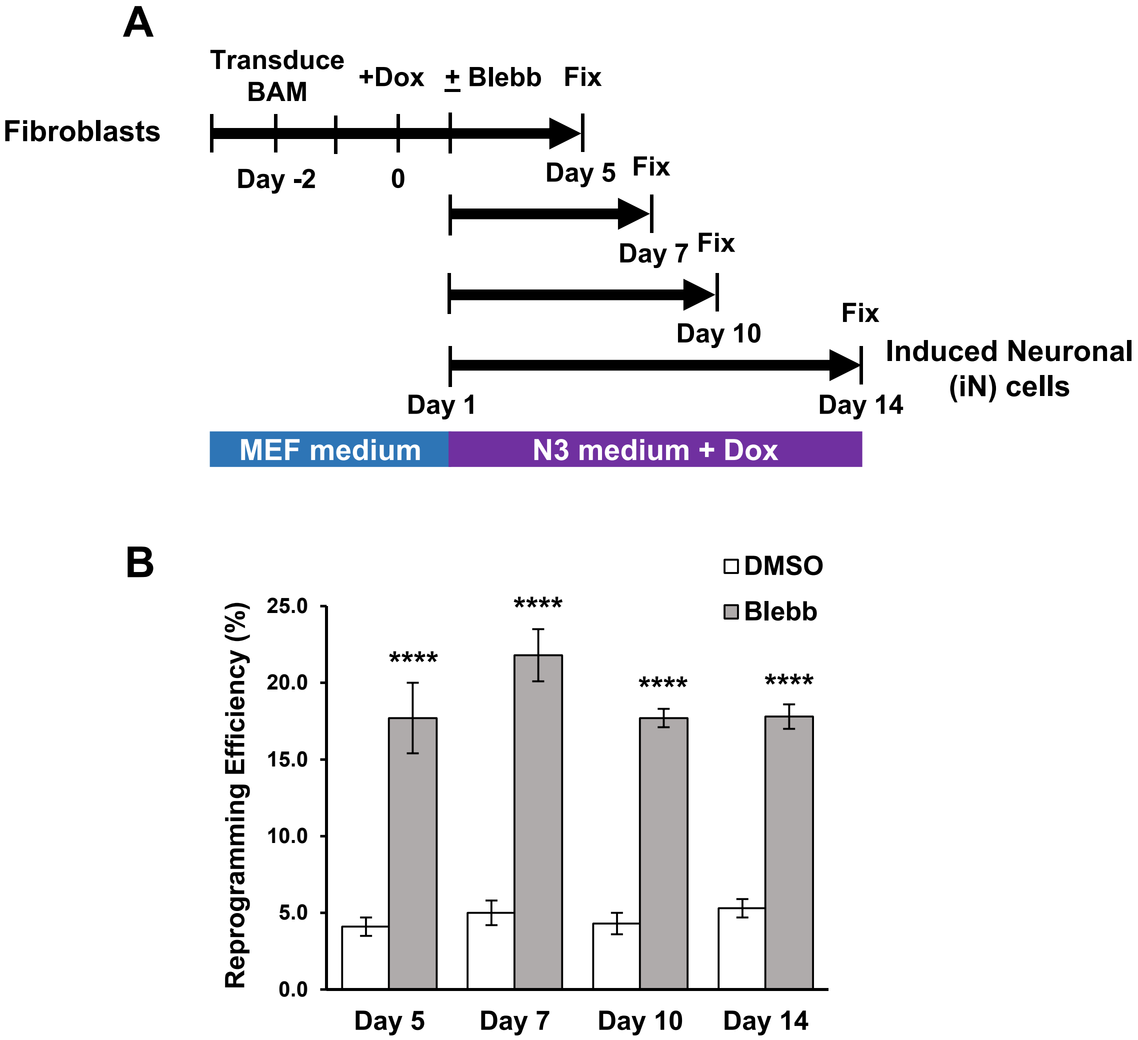

Figure S12. The effects of cytoskeletal contractility inhibition on iN reprogramming efficiency. A, Schematic illustrating the time points during the reprogramming process in which the cultures were fixed. **B,** Reprogramming efficiency at various time points during the reprogramming process. Blebbistatin (10 μM) was administered for 7 days (n=3). Significance determined by two-way ANOVA using Sidak's multiple comparison test. Bar graph shows mean ± standard deviation (****p < 0.0001).

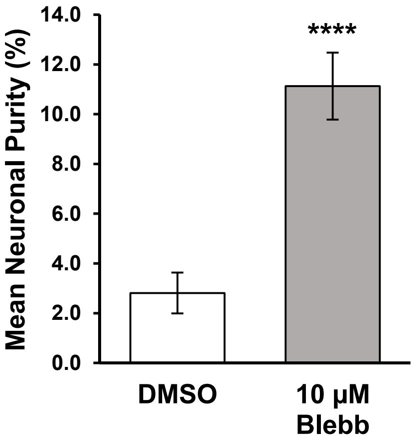

Figure S13. The effects of cytoskeletal disruption on neuronal purity. Neuronal purity of cultures derived from BAM-reprogrammed fibroblasts treated with or without 10 µM blebbistatin at day 14 (n=7). Neuronal purity was determined as the percentage of $TUBB3^{+}$ iN cells normalized to the number of cells present at day 14. Significance determined by two-tailed, student's *t* test, compared to DMSO. Bar graph shows mean ± standard deviation (****$p < 0.0001$).

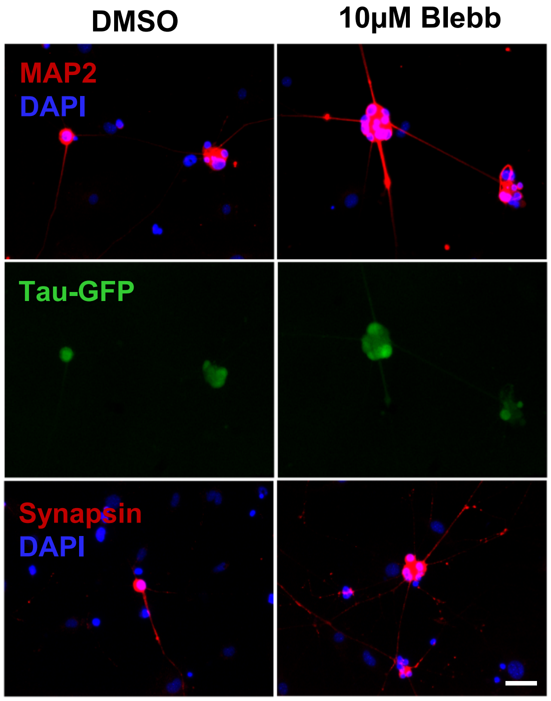

Figure S14. Tau-eGFP fibroblast-derived iN cells express mature neuronal markers. Immunofluorescent images of Tau-eGFP fibroblast-derived iN cells co-expressing neuronal markers Tau-GFP, MAP2 and synapsin at day 21. Scale bar, 50 μm.

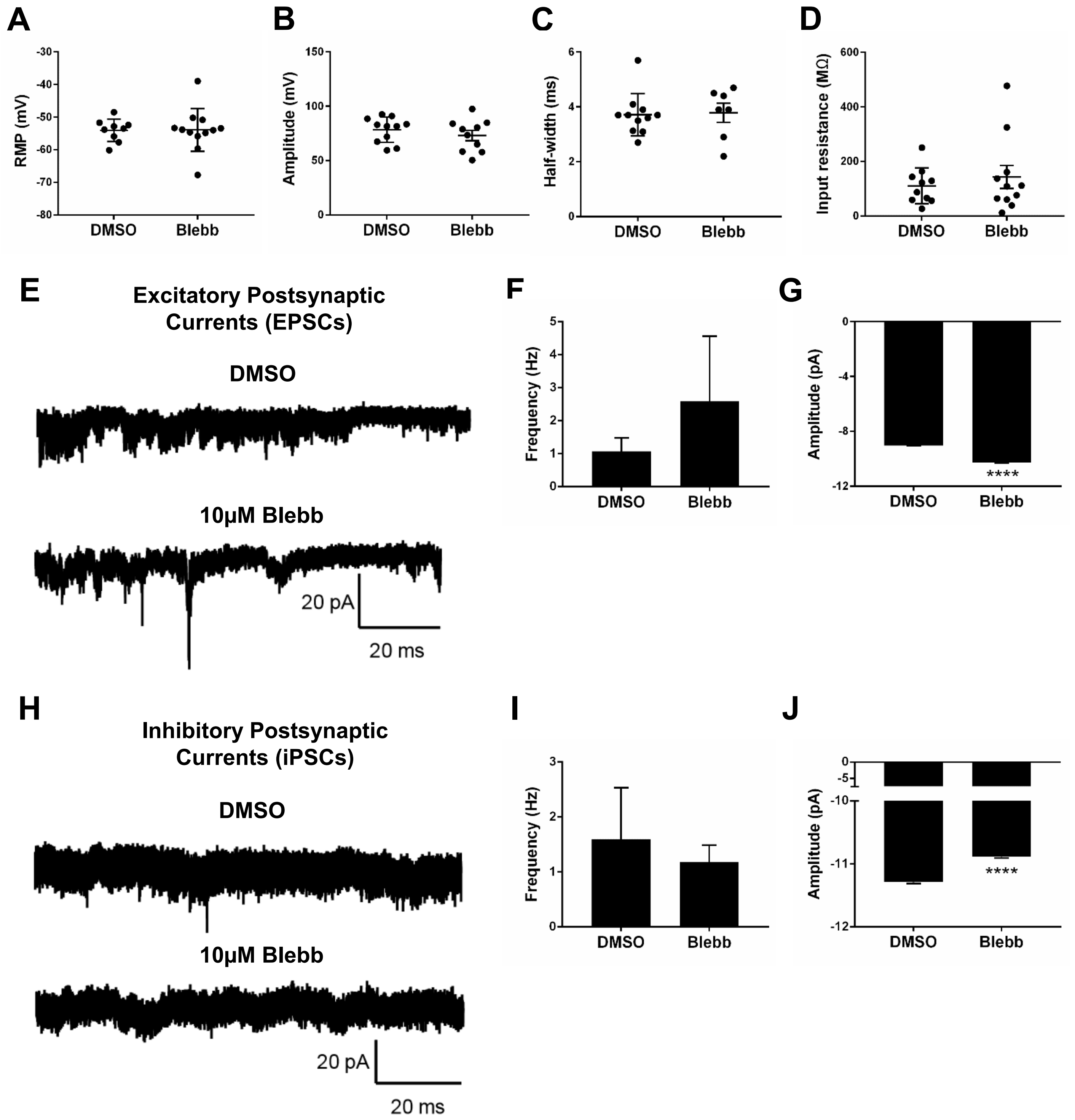

Figure S15. Functional assessment of derived iN cells by electrophysiological analysis. A-D, Quantification of electrophysiological properties of iN cells derived with and without blebbistatin. Each circle represents an individual cell that was tested. The resting membrane potential, RMP (**A**), action potential amplitude (**B**) and half-width (**C**) and input resistance (**D**) were measured. **E,** Representative recordings of spontaneous excitatory postsynaptic currents (EPSCs) from iN cells derived in the absence and presence of blebbistatin. **F-G,** Quantification of EPSC frequency (**F**) and amplitude (**G**) of iN cells (n=6). **H,** Representative traces of spontaneous inhibitory postsynaptic currents (IPSCs) in iN cells derived in the absence and presence of blebbistatin. **I-J.** Quantification of IPSC frequency (**I**) and amplitude (**J**) of iN cells (n=4). Significance determined by two-tailed, student's *t* test, compared to DMSO condition. Bar graphs represent mean ± one standard error of mean (*****p* < 0.0001).

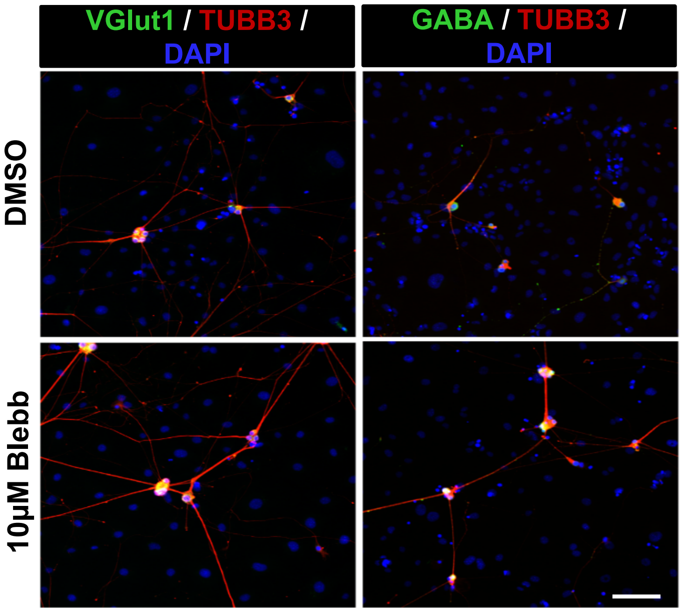

Figure S16. iN cells were identified as glutamatergic and GABAergic neuronal subtypes. Immunostaining images of TUBB3⁺ iN cells derived in the absence and presence of 10 μM blebbistatin expressing VGlut1 and GABA at day 23. Scale bar, 100 μm.

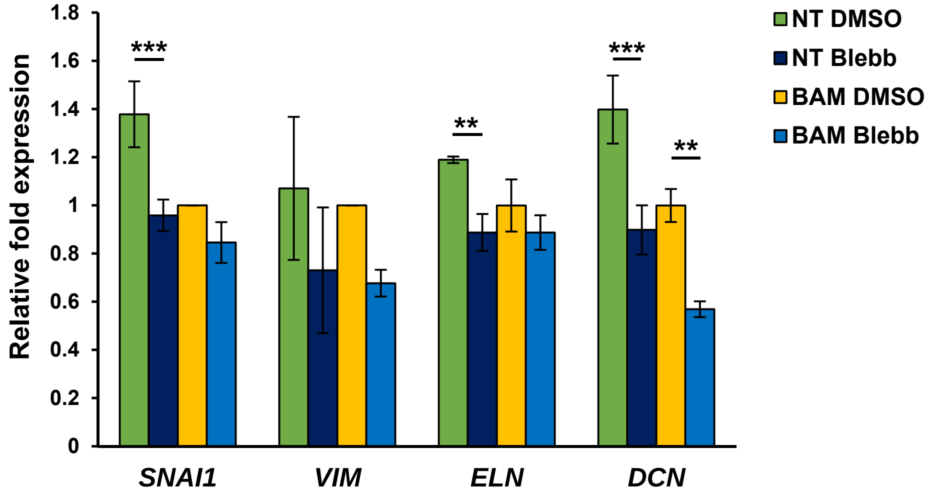

Figure S17. Blebbistatin downregulates mesenchymal genes. Non-transduced (NT) and BAM-transduced fibroblasts treated with blebbistatin for 2 days followed by qRT-PCR analysis of mesenchymal and fibroblast gene expression at day 3 (n=3). Significance determined by one-way ANOVA and Sidak's multiple comparison test. Gene expression was normalized with 18S RNA levels. Bar graph represents mean ± one standard deviation (** $p < 0.01$, *** $p < 0.001$).

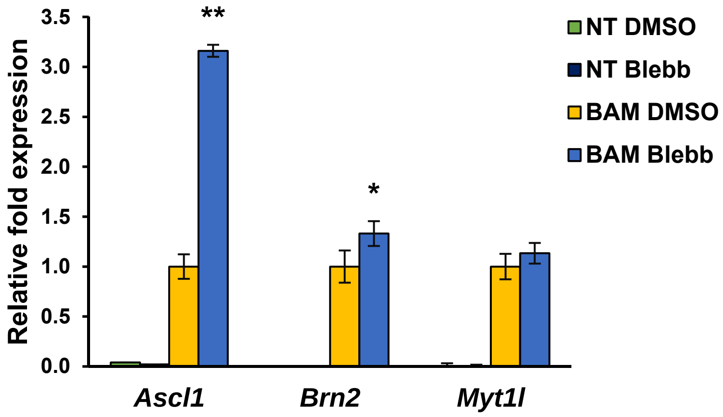

Figure S18. Blebbistatin upregulates neuronal gene expression. Non-transduced (NT) and BAM-transduced fibroblasts were treated with blebbistatin for 2 days followed by qRT-PCR analysis of neuronal gene expression at day 3 (n=3). Significance determined by one-way ANOVA and Tukey's multiple comparison test. Significance determined by two-tailed, student's *t* test, compared to DMSO condition for the same gene. Gene expression was normalized with 18S RNA levels. Bar graphs represent mean ± one standard deviation (*$p < 0.05$, **$p < 0.01$).

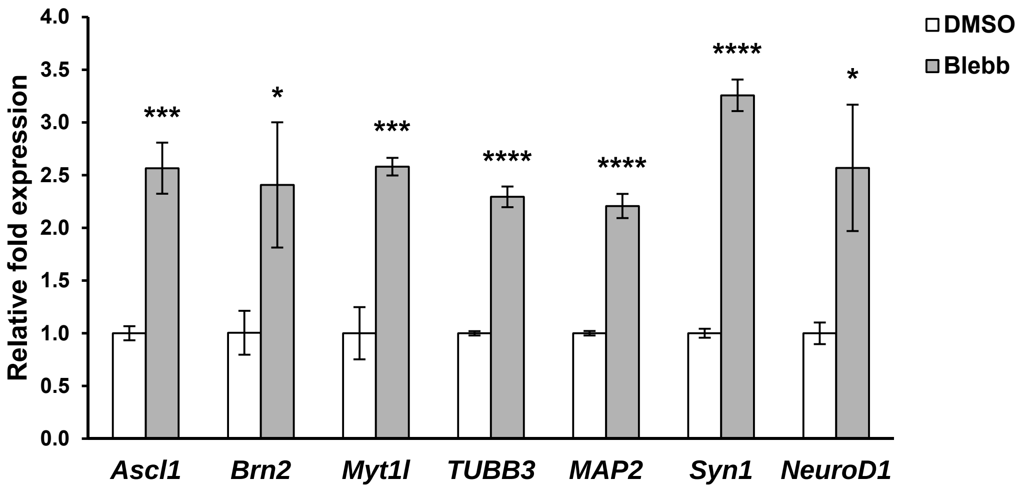

Figure S19. Blebbistatin upregulates neuronal genes in Ascl1-mediated iN reprogramming. qRT-PCR analysis of neuronal gene expression at day 7 in Ascl1-transduced fibroblasts cultured in the absence and presence of blebbistatin (n=3). Significance determined by two-tailed, student's *t* test, compared to DMSO condition for the same gene. Gene expression was normalized with 18S RNA levels. Bar graphs represent mean ± one standard deviation (*$p < 0.05$, ***$p < 0.001$, ****$p < 0.0001$).

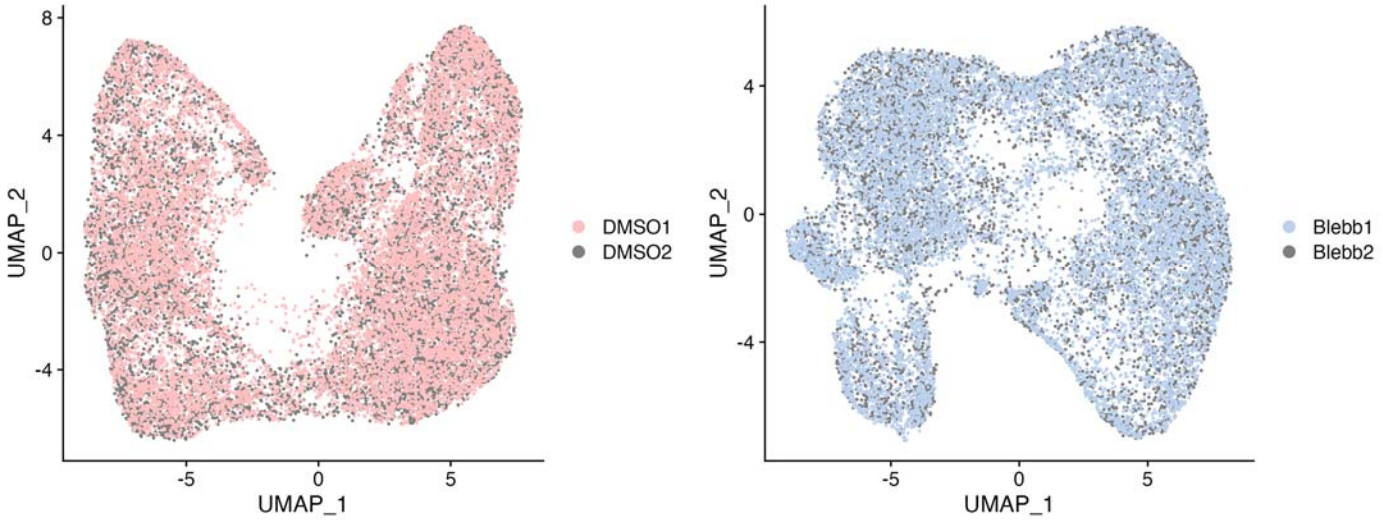

Figure S20. Single cell RNA sequencing samples show sample consistency. UMAP clustering of single cell RNA sequencing data from independent biological replicates of BAM-transduced fibroblasts reprogrammed in the absence or presence of blebbistatin for 48 hours and collected on day 3 (n=2).

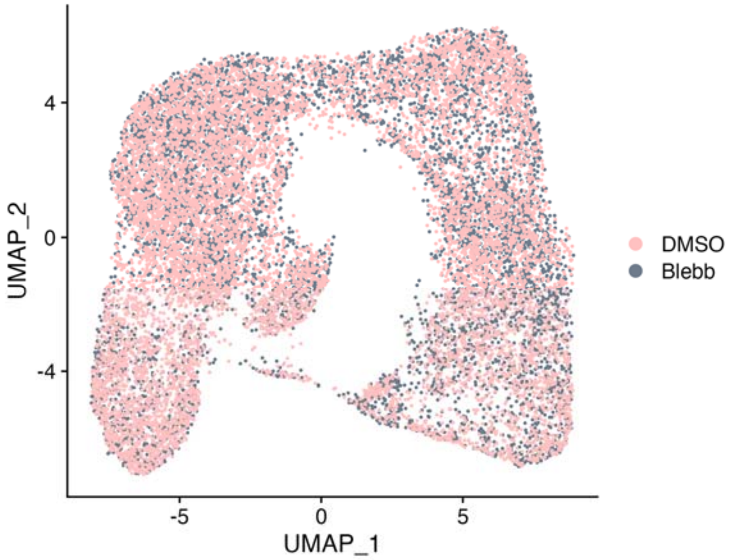

Figure S21. UMAP clustering of single cell RNA sequencing samples. UMAP clustering of single cell RNA sequencing data from samples of BAM-transduced fibroblasts reprogrammed in the absence or presence of blebbistatin for 48 hours and collected on day 3 (n=2).

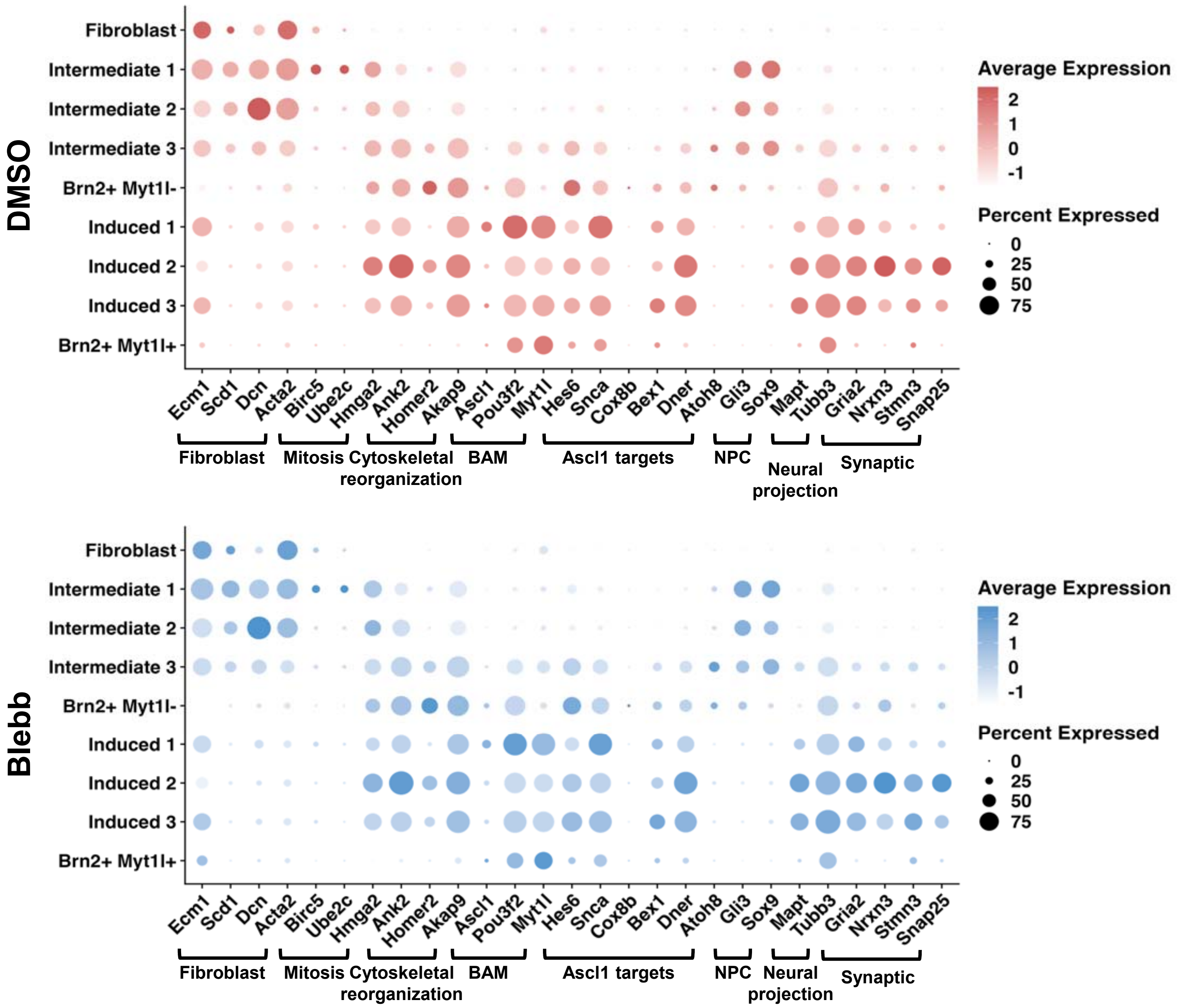

Figure S22. Differential gene expression in cell clusters based on single cell RNA sequencing. (A) Dot plot showing the gene expression of selected markers of fibroblasts, mitosis, cytoskeletal reorganization, BAM, Ascl1 targets, neural progenitor cells (NPC), neural projection and synaptic genes in each cell cluster. The color from light to dark indicates low to high expression level, respectively, and negative values of average expression correspond to clusters with expression levels below the mean expression across the whole dataset. The dot size indicates the percentage of cells expressing that corresponding gene.

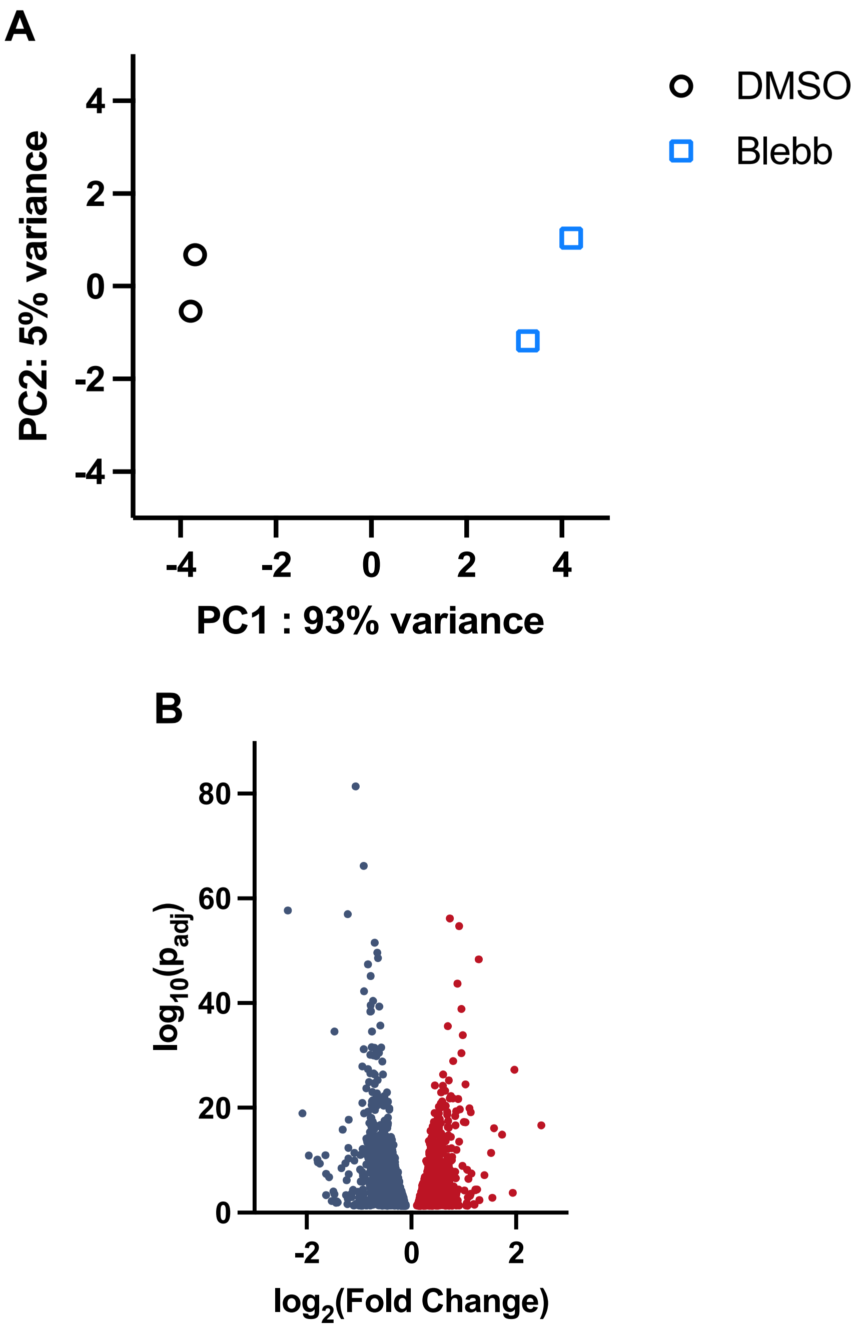

Figure S23. Differential gene expression in cell clusters based on single cell RNA sequencing. (A) Principal component analysis (PCA) of single cell RNA sequencing data from independent biological replicates of BAM-transduced fibroblasts reprogrammed in the absence or presence of blebbistatin for 48 hours and collected on day 3 (n=2). **(B)** Volcano plot showing the average log-fold-change and average adjusted p-values for pairwise differential expression between pseudobulk cells from each DMSO- and blebbistatin-treated sample.

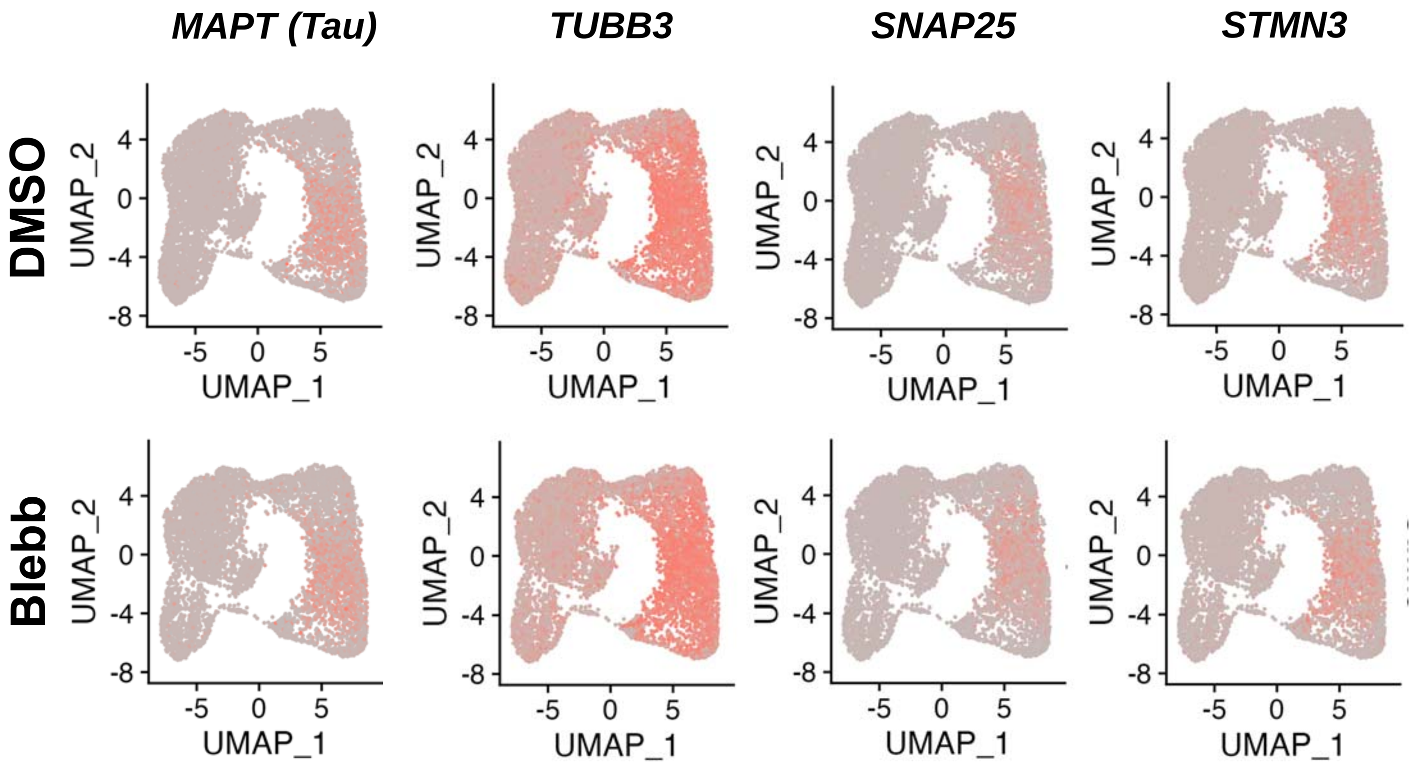

Figure S24. Differential neuronal gene expression in cell clusters based on single cell RNA sequencing. Scatter plots showing the expression level (log10(RPM)) of selected neuronal genes on UMAP plots. Each dot represents a single cell.

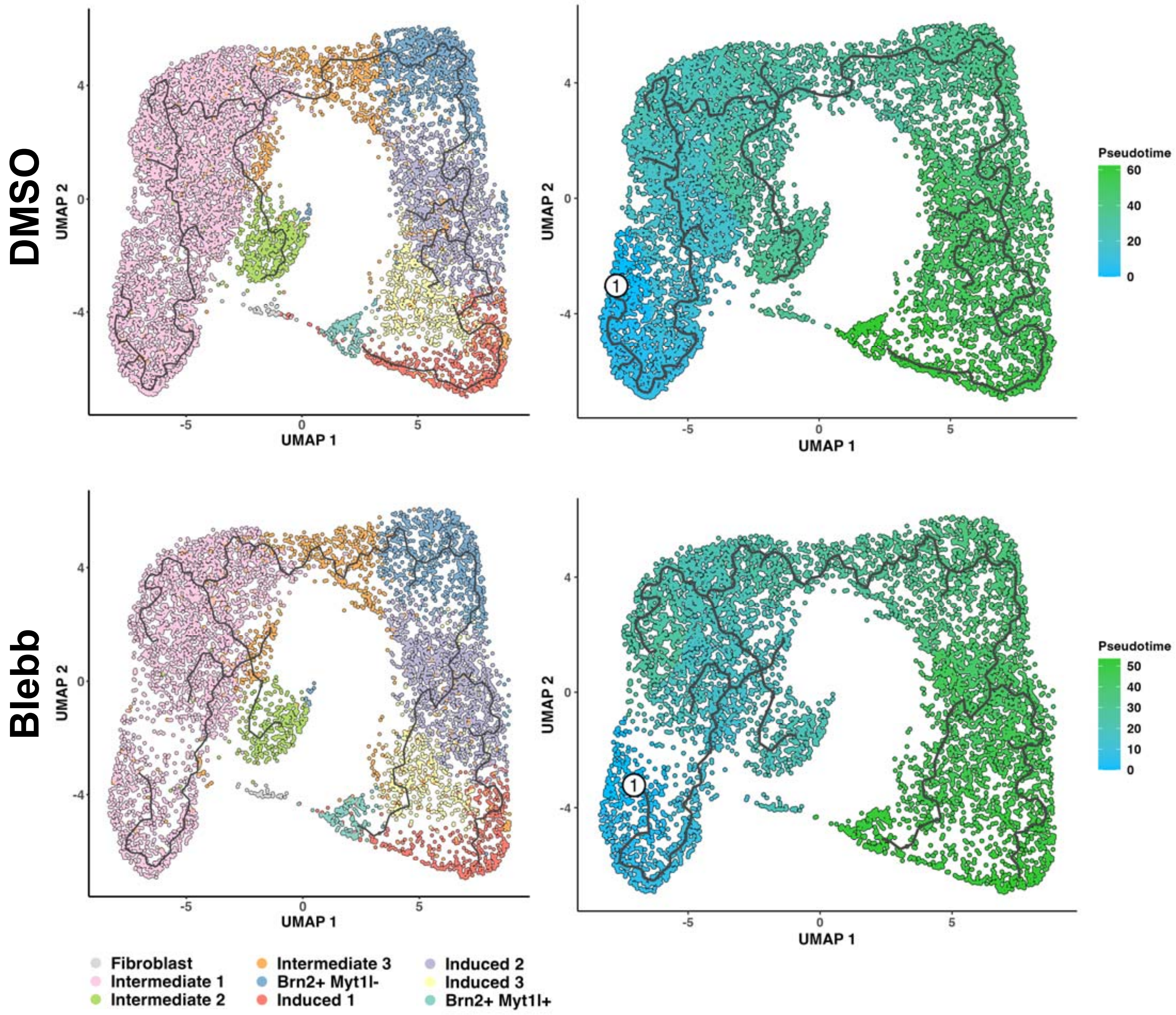

Figure S25. Single cell trajectory constructed using Monocle3 based on all expressed genes. Monocle3 classified cells into 9 “States” for BAM-transduced fibroblasts treated with DMSO or blebbistatin (Blebb) on day 3 of the reprogramming process. Different colors represent different states/clusters. Panels on the right illustrate results of pseudotime analysis. BAM-transduced fibroblasts treated with DMSO or blebbistatin show a similar reprogramming trajectory.

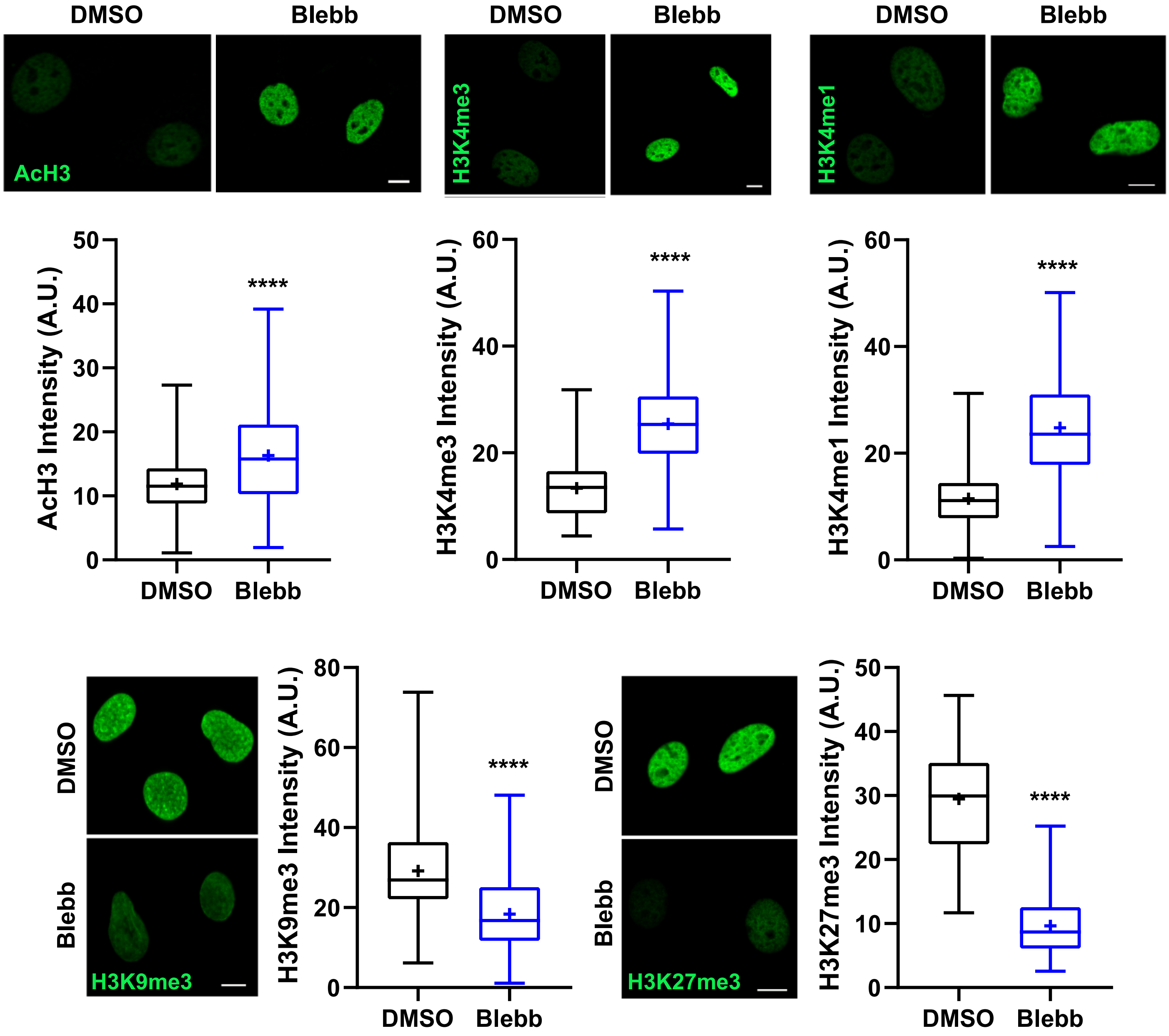

Figure S26. Effect of cytoskeletal contractility inhibition on the global levels of various histone proteins. Representative images show the level and distribution of various histone marks in non-transduced fibroblasts treated with DMSO or 10 μM blebbistatin for 2 hours. Scale bar, 10 μm. Quantification of histone mark intensity based on immunofluorescent images, a.u.: arbitrary unit (AcH3, n= 147 for DMSO, n= 152 for Blebb; H3K4me3, n= 147 for DMSO, n= 161 for Blebb; H3K4me1, n= 180 for DMSO, n= 129 for Blebb; H3K27me3, n= 91 for DMSO, n= 131 for Blebb; H3K9me3, n= 104 for DMSO, n= 119 for Blebb). Significance determined by two-tailed, student's *t* test, compared to the DMSO condition. Box plots show the ends at the quartiles, the median as a horizontal line in the box, the mean as a (+) symbol, and the whiskers extend from the minimum to maximum data point (****$p < 0.0001$).

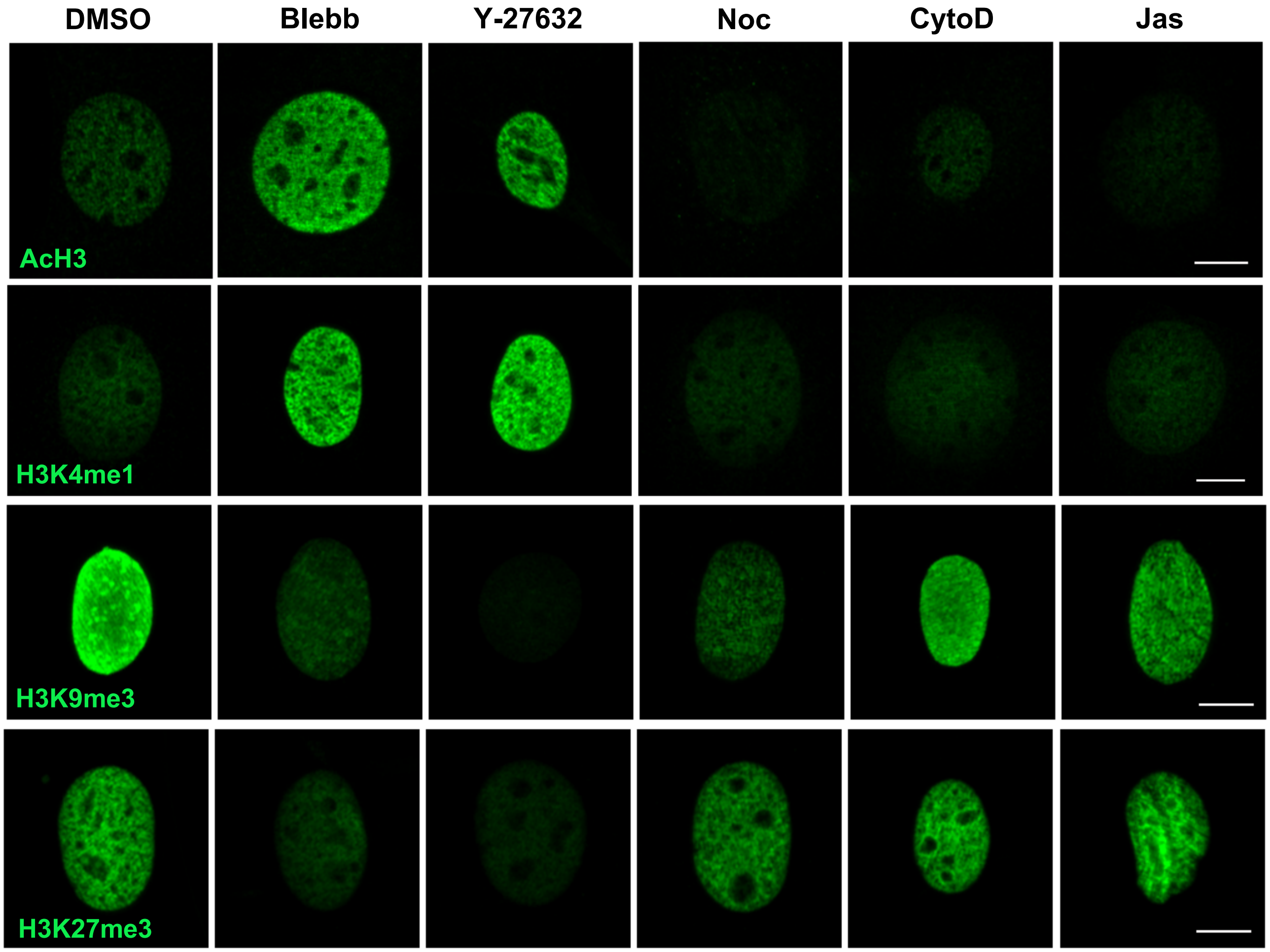

Figure S27. Effect of cytoskeletal disruption on the global levels of various histone proteins. Immunofluorescent images show the level and distribution of various histone marks in non-transduced fibroblasts treated with DMSO, 10 μM blebbistatin (Blebb), 20 μM Y-27632, 0.3 μM Nocodazole (Noc), 1 μM Cytochalasin D (CytoD), and 0.05 μM Jasplakinolide (Jas) for 2 hours. Scale bar, 10 μm.

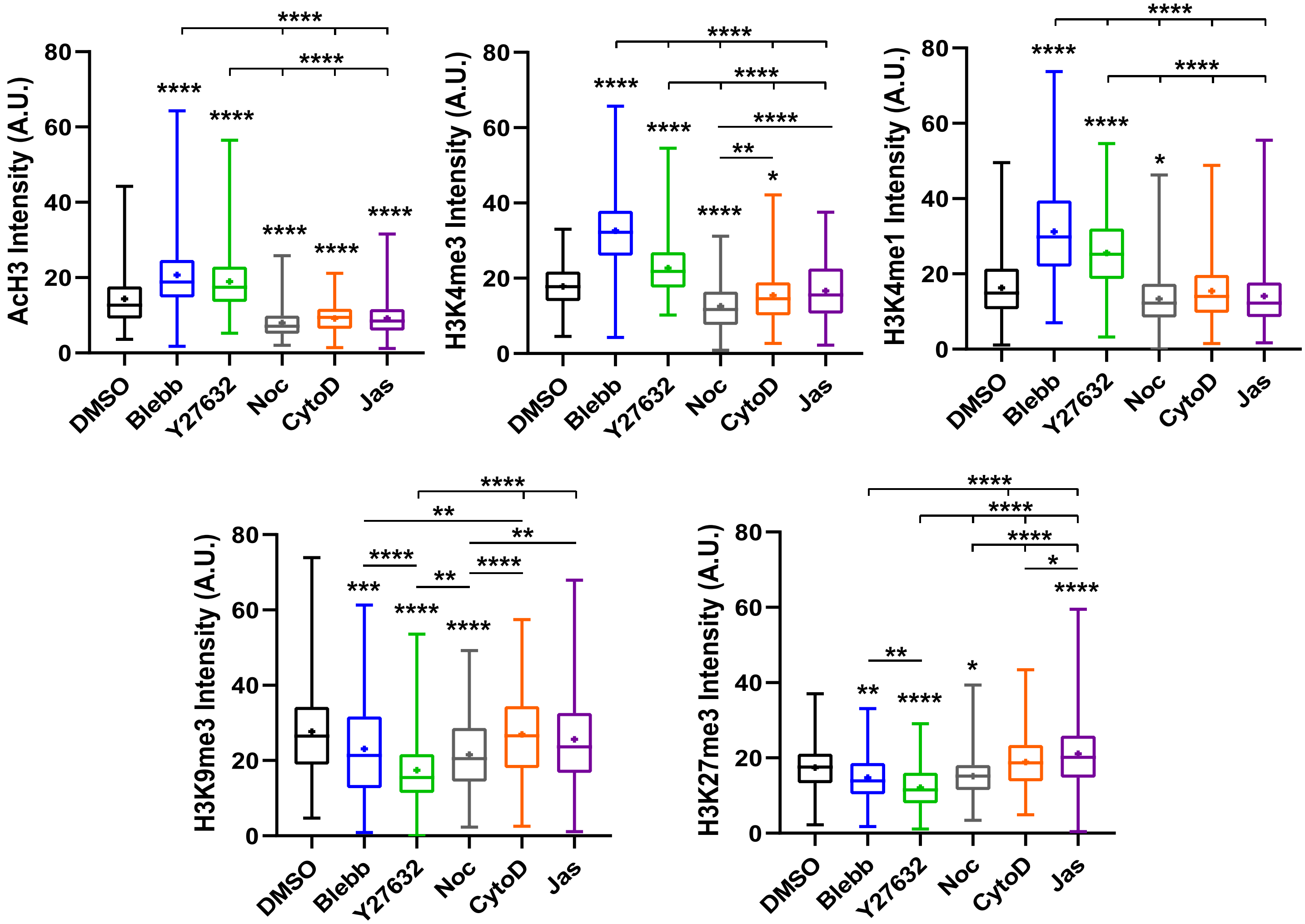

Figure S28. Effect of cytoskeletal disruption on the global levels of various histone proteins. Non-transduced fibroblasts were treated with DMSO, 10 μM blebbistatin (Blebb), 20 μM Y-27632, 0.3 μM Nocodazole (Noc), 1 μM Cytochalasin D (CytoD), and 0.05 μM Jasplakinolide (Jas) for 2 hours, followed by immunostaining for various histone marks. Quantification of histone mark intensity based on immunofluorescent images (Supplementary Fig. S17), a.u.: arbitrary unit (AcH3, n= 131 for DMSO, n= 255 for Blebb, n= 166 for Y-27632, n= 153 for Noc, n= 155 for CytoD, n= 213 for Jas; H3K4me3, n= 207 for DMSO, n= 332 for Blebb, n= 237 for Y-27632, n= 232 for Noc, n= 182 for CytoD, n= 199 for Jas; H3K4me1, n= 193 for DMSO, n= 318 for Blebb, n= 244 for Y-27632, n= 275 for Noc, n= 235 for CytoD, n= 211 for Jas; H3K27me3, n= 199 for DMSO, n= 143 for Blebb, n= 202 for Y-27632, n= 193 for Noc, n= 217 for CytoD, n= 236 for Jas; H3K9me3, n= 231 for DMSO, n= 240 for Blebb, n= 218 for Y-27632, n= 199 for Noc, n= 231 for CytoD, n= 232 for Jas). Significance determined by one-way ANOVA using Tukey's correction for multiple comparisons (**p* < 0.05, ***p* < 0.01, ****p* < 0.001, *****p* < 0.0001). Box plots show the ends at the quartiles, the median as a horizontal line in the box, the mean as a (+) symbol, and the whiskers extend from the minimum to maximum data point.

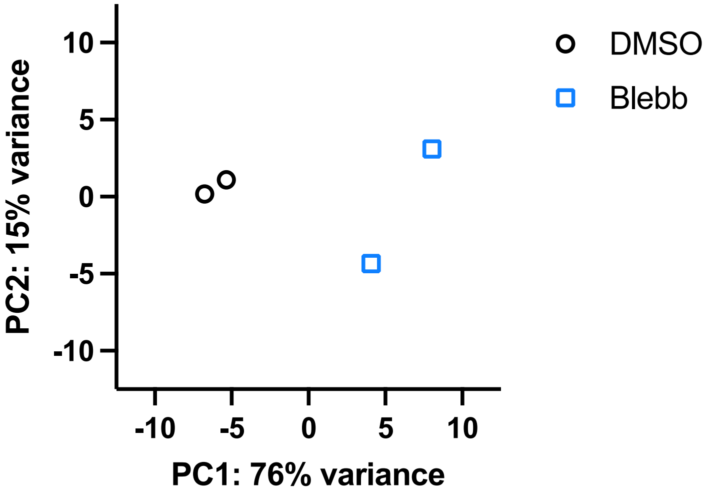

Figure S29. Effect of cytoskeletal tension disruption on chromatin accessibility. Principal component analysis (PCA) of ATAC-seq data from independent biological replicates of fibroblasts cultured with vehicle control (DMSO) or 10 μM blebbistatin for 2 hours (n=2).

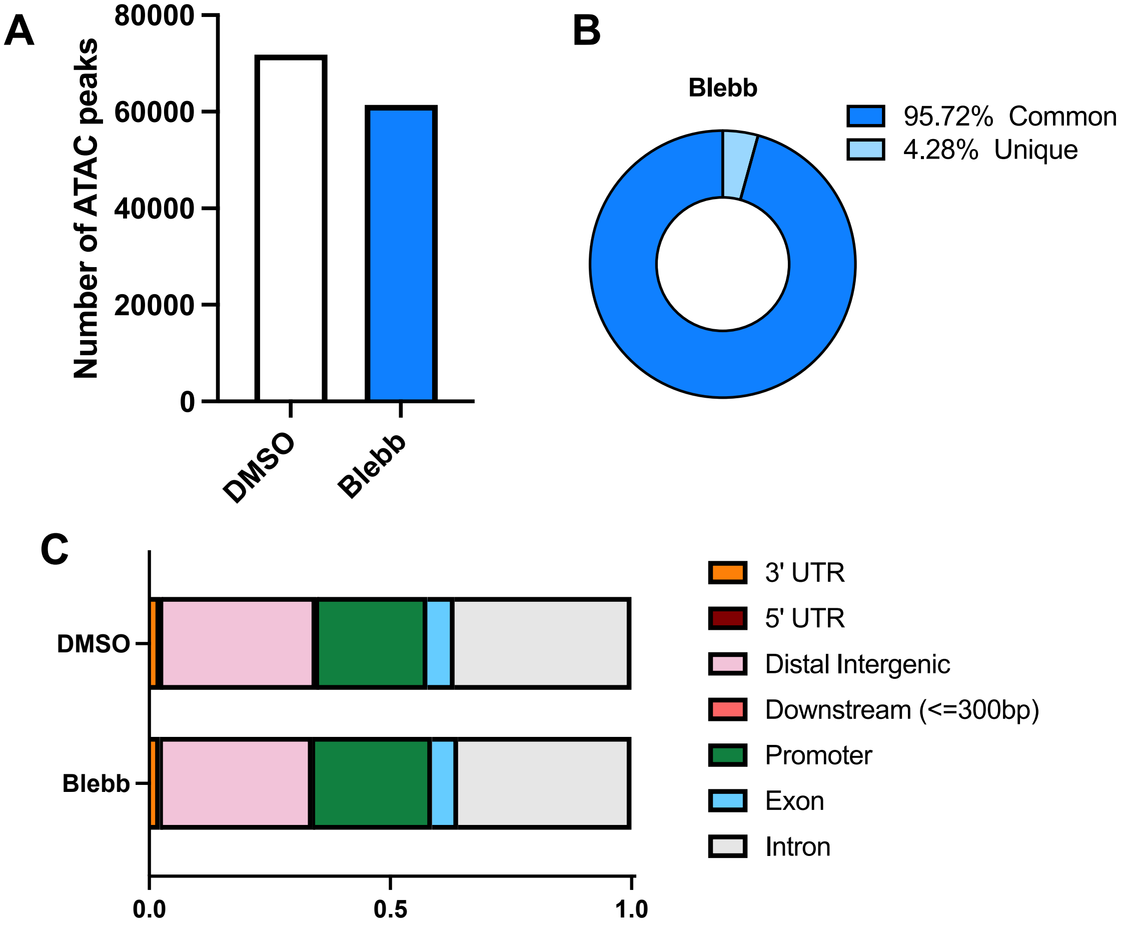

Figure S30. Effect of cytoskeletal tension disruption on chromatin accessibility. **A,** Number of ATAC-seq peaks from independent biological replicates of fibroblasts cultured with vehicle control (DMSO) or 10 μM blebbistatin for 2 hours (n=2). **B,** Percentage of unique and common peaks in blebbistatin-treated fibroblasts relative to DMSO-treated fibroblasts based on ATAC-seq data. **C,** Genomic annotations of ATAC peaks for the indicated conditions.

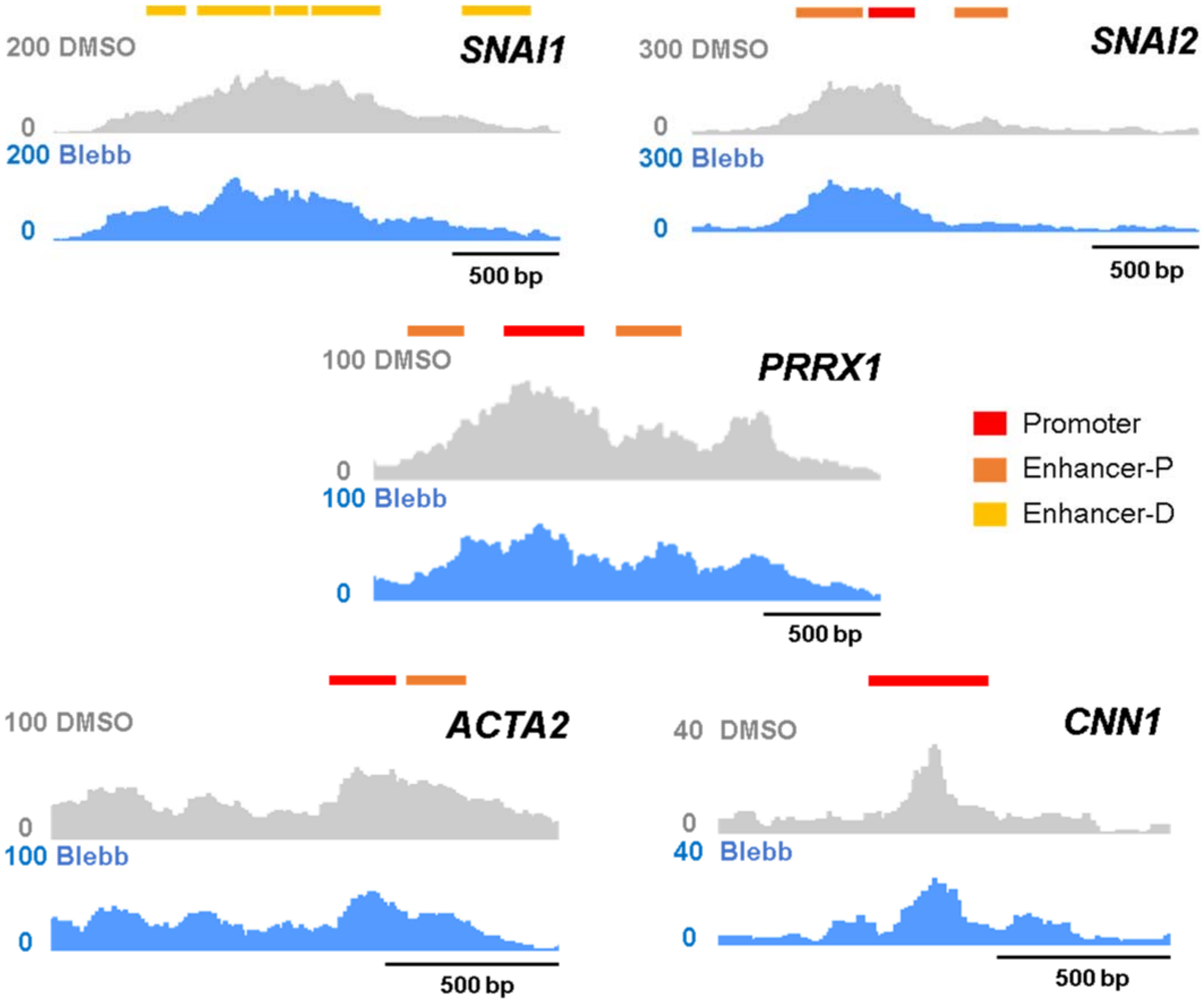

Figure S31. Disruption of cytoskeletal tension altered chromatin accessibility around fibroblast-related genomic loci. ATAC-seq tracks for *SNAI1*, *SNAI2, PRRX1, ACTA2 and CNN1 g*enomic loci from fibroblasts treated with DMSO or 10 μM blebbistatin for 2 hours. Blebb-induced chromatin remodeling occurred near promoter, proximal enhancer (Enhancer-P) and distal enhancer (Enhancer-D) sites.

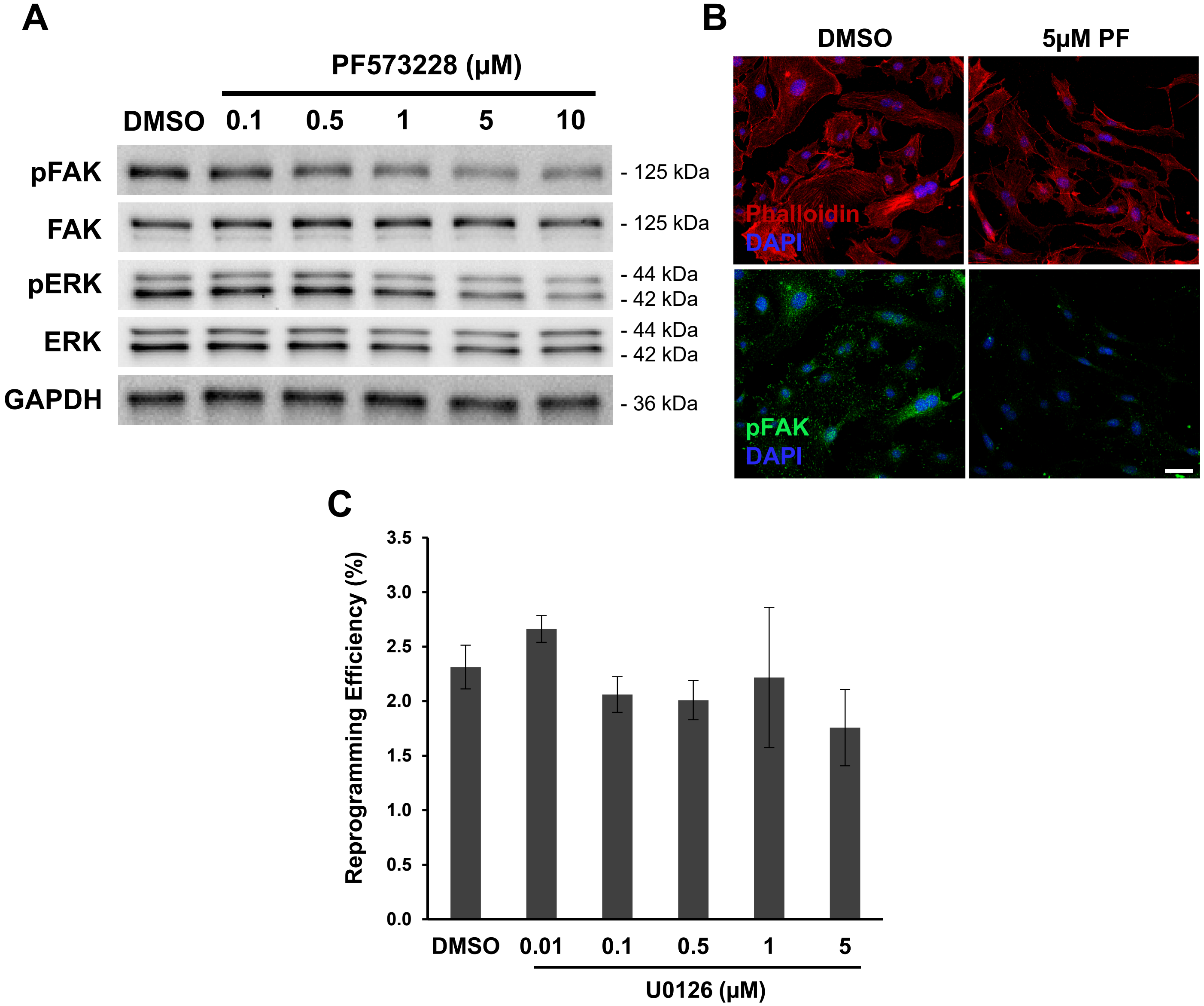

Figure S32. PF573228 specifically inhibits FAK activity. A, Western blot analysis of fibroblasts treated with varying doses of the FAK inhibitor, PF573228 (PF). PF573228 inhibits the phosphorylation of FAK (pFAK) and downstream ERK in a dose-dependent manner. **B,** Immunofluorescence micrographs illustrate the cell morphology and expression of pFAK in fibroblasts treated with 5 μM PF for 24 hours. Scale bar, 50 μm. **C,** Reprogramming efficiency of BAM-transduced fibroblasts treated with various concentrations of the ERK inhibitor, U0126, at day 14 (n=3). No statistical significance was determined using a one-way ANOVA and Dunnett's multiple comparison test.

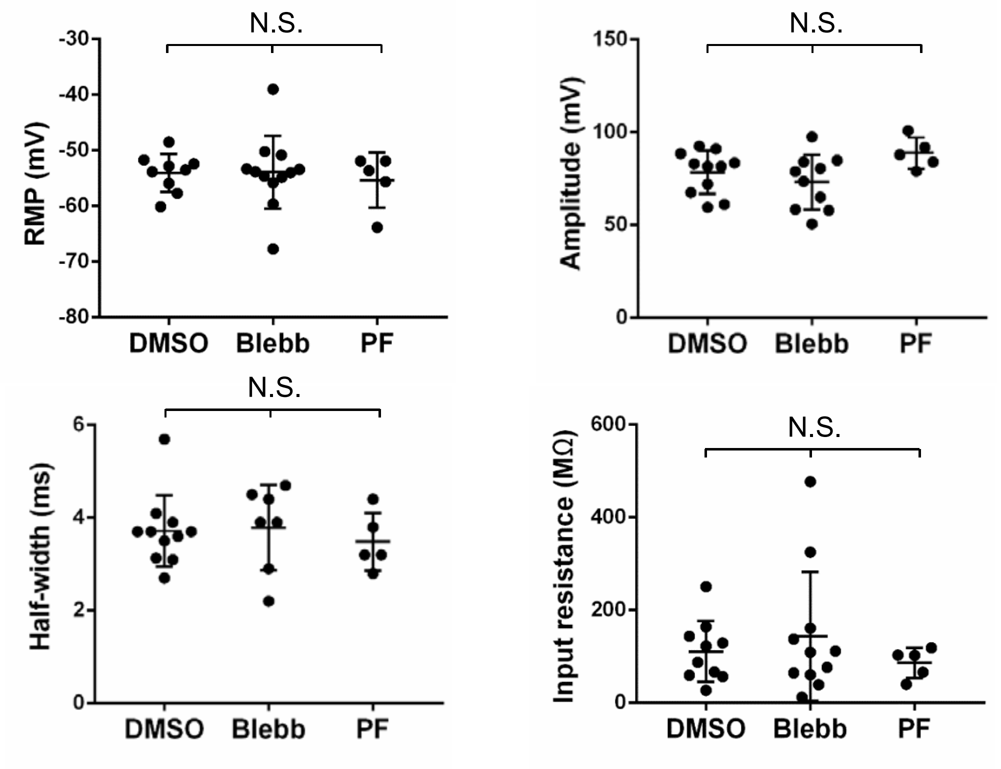

Figure S33. PF573228-derived iN cells display functionality. Quantification of electrophysiological properties of iN cells obtained in the absence and presence of 10 μM blebbistatin (Blebb) or 1 μM PF573228 (PF). The inhibitor was administered during the first 7 days of reprogramming. Each circle represents an individual cell that was tested. The resting membrane potential, RMP, input resistance and action potential amplitude and half-width were measured. Significance determined by one-way ANOVA using Tukey's correction for multiple comparisons (NS: not significant).

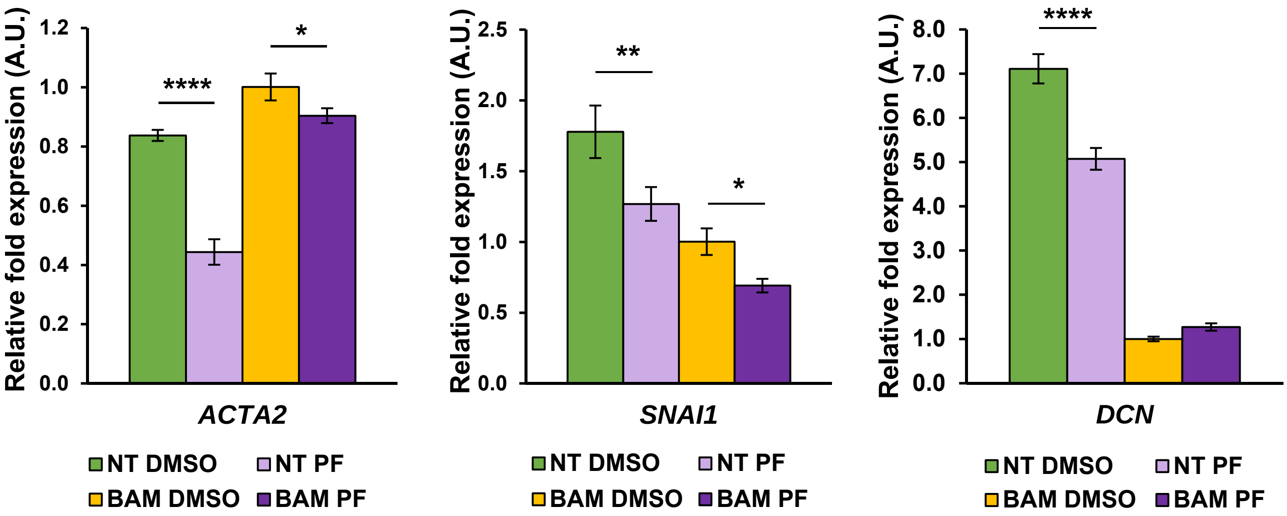

Figure S34. FAK inhibition downregulates mesenchymal genes. Non-transduced (NT) and BAM-transduced fibroblasts treated with PF573228 for 1 day followed by qRT-PCR analysis of mesenchymal and fibroblast gene expression at day 2 (n=3). Significance determined by one-way ANOVA and Sidak's multiple comparison test. Gene expression was normalized with 18S RNA levels. Bar graph represents mean ± one standard deviation (*$p < 0.05$, **$p < 0.01$, ****$p < 0.0001$).

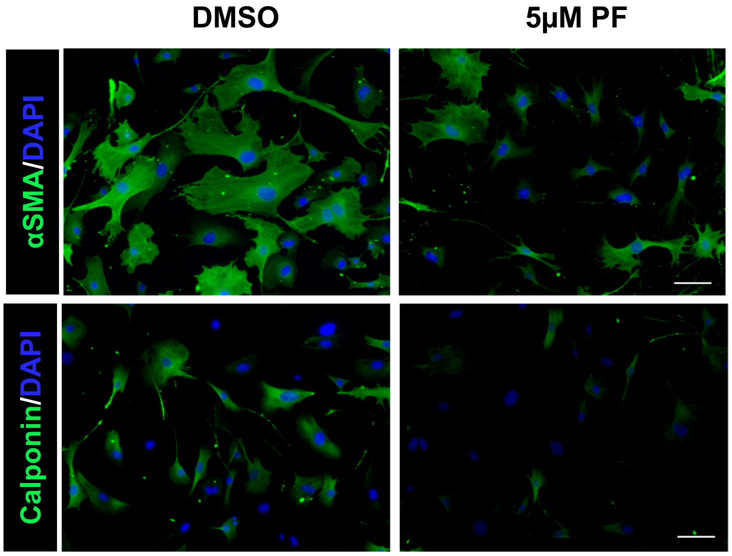

Figure S35. FAK inhibition decreases mesenchymal marker expression. Immunofluorescence micrographs illustrate the expression of αSMA (top panels) and calponin (bottom panels) in fibroblasts treated with DMSO or 5 µM PF573228 for 24 hours. Scale bar, 100 µm.

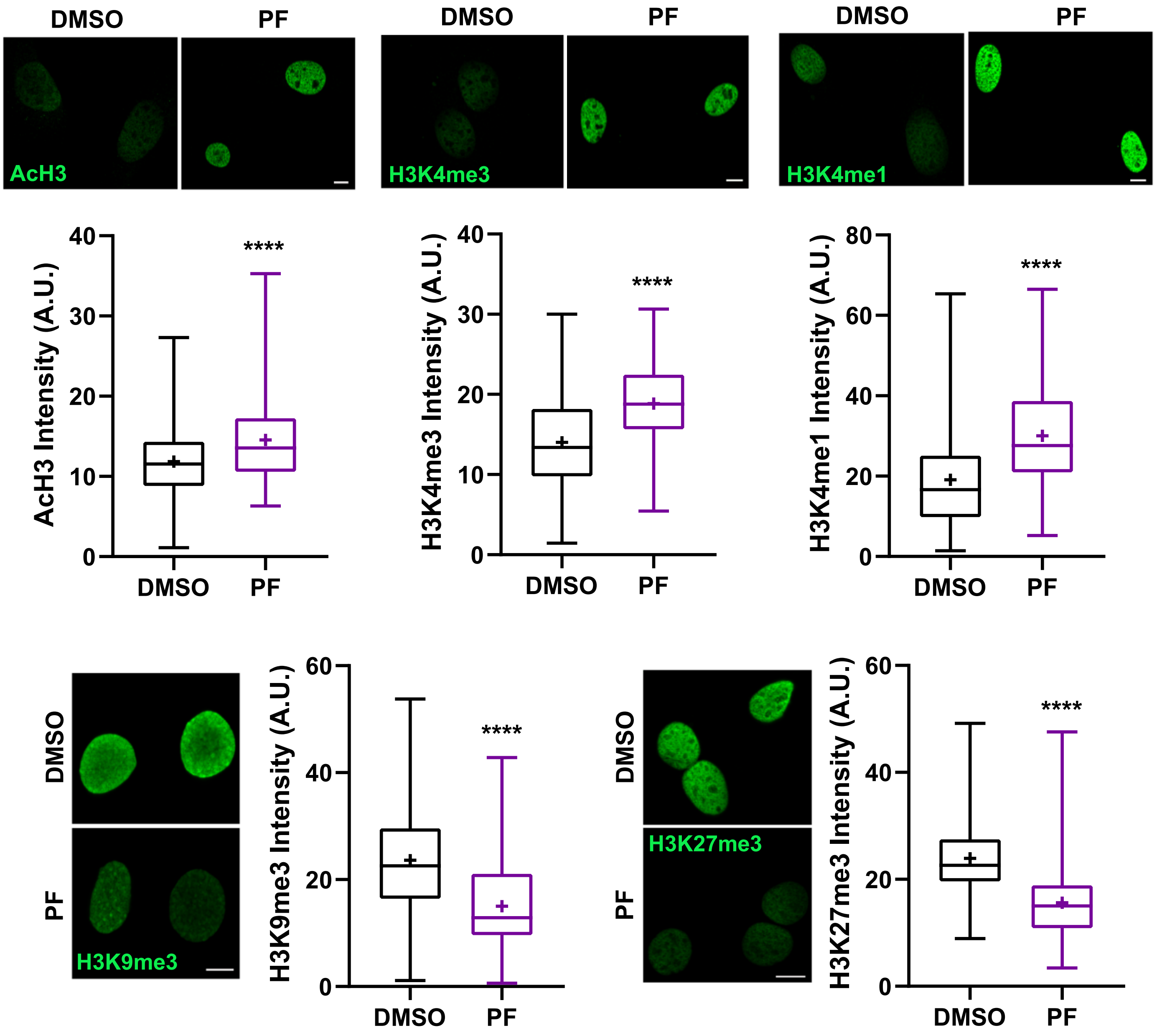

Figure S36. Effect of focal adhesion disruption on the global levels of various histone proteins. Representative images show the level and distribution of various histone marks in non-transduced fibroblasts treated with DMSO or 5 μM PF573228 for 2 hours. Scale bar, 10 μm. Quantification of histone mark intensity based on immunofluorescent images, a.u.: arbitrary unit (AcH3, n= 147 for DMSO, n= 110 for PF; H3K4me3, n= 147 for DMSO, n= 161 for PF; H3K4me1, n= 180 for DMSO, n= 129 for PF; H3K9me3, n= 106 for DMSO, n= 106 for PF; H3K27me3, n= 83 for DMSO, n= 128 for PF). Significance determined by two-tailed, student's *t* test, compared to the DMSO condition. Box plots show the ends at the quartiles, the median as a horizontal line in the box, the mean as a (+) symbol, and the whiskers extend from the minimum to maximum data point (****$p < 0.0001$).

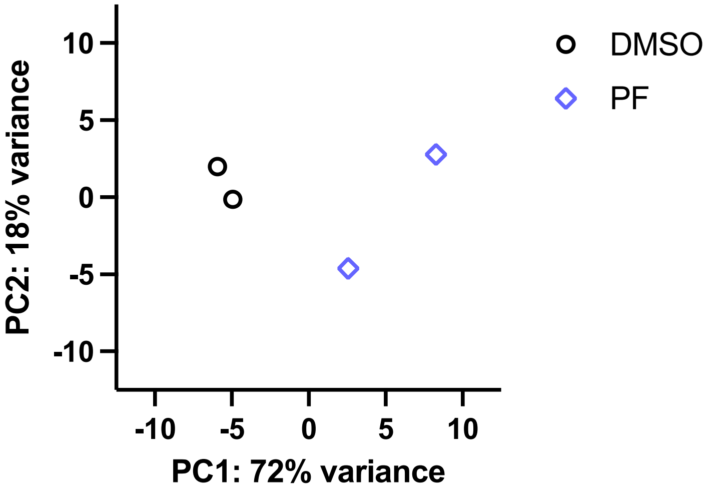

Figure S37. Effect of cell adhesion disruption on chromatin accessibility. Principal component analysis (PCA) of ATAC-seq data from independent biological replicates of fibroblasts cultured with vehicle control (DMSO) or 5 µM PF573228 for 2 hours (n=2).

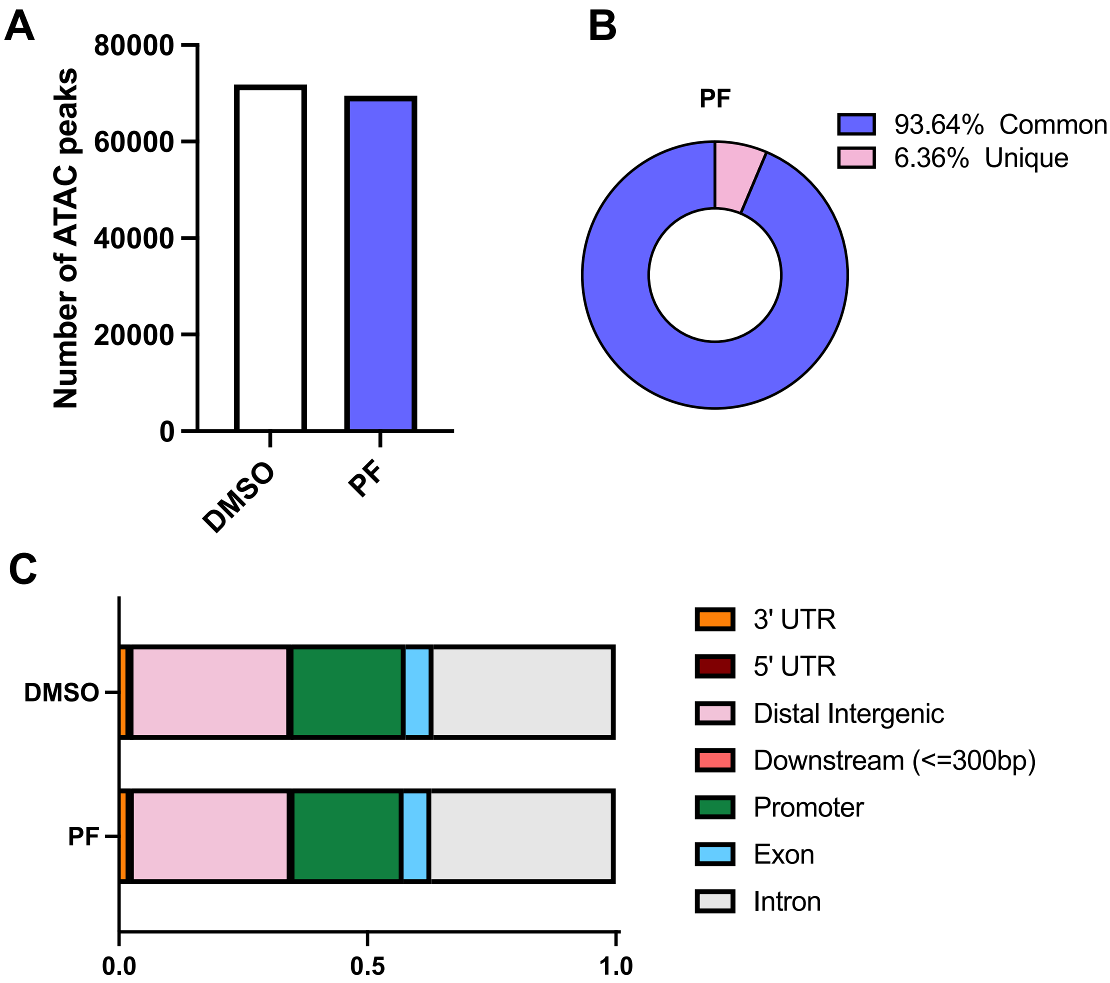

Figure S38. Effect of cell adhesion disruption on chromatin accessibility. A, Number of ATAC-seq peaks from independent biological replicates of fibroblasts cultured with vehicle control (DMSO) or 5 µM PF573228 for 2 hours (n=2). **B,** Percentage of unique and common peaks in PF573228-treated fibroblasts relative to DMSO-treated fibroblasts based on ATAC-seq data. **C,** Genomic annotations of ATAC peaks for the indicated conditions.

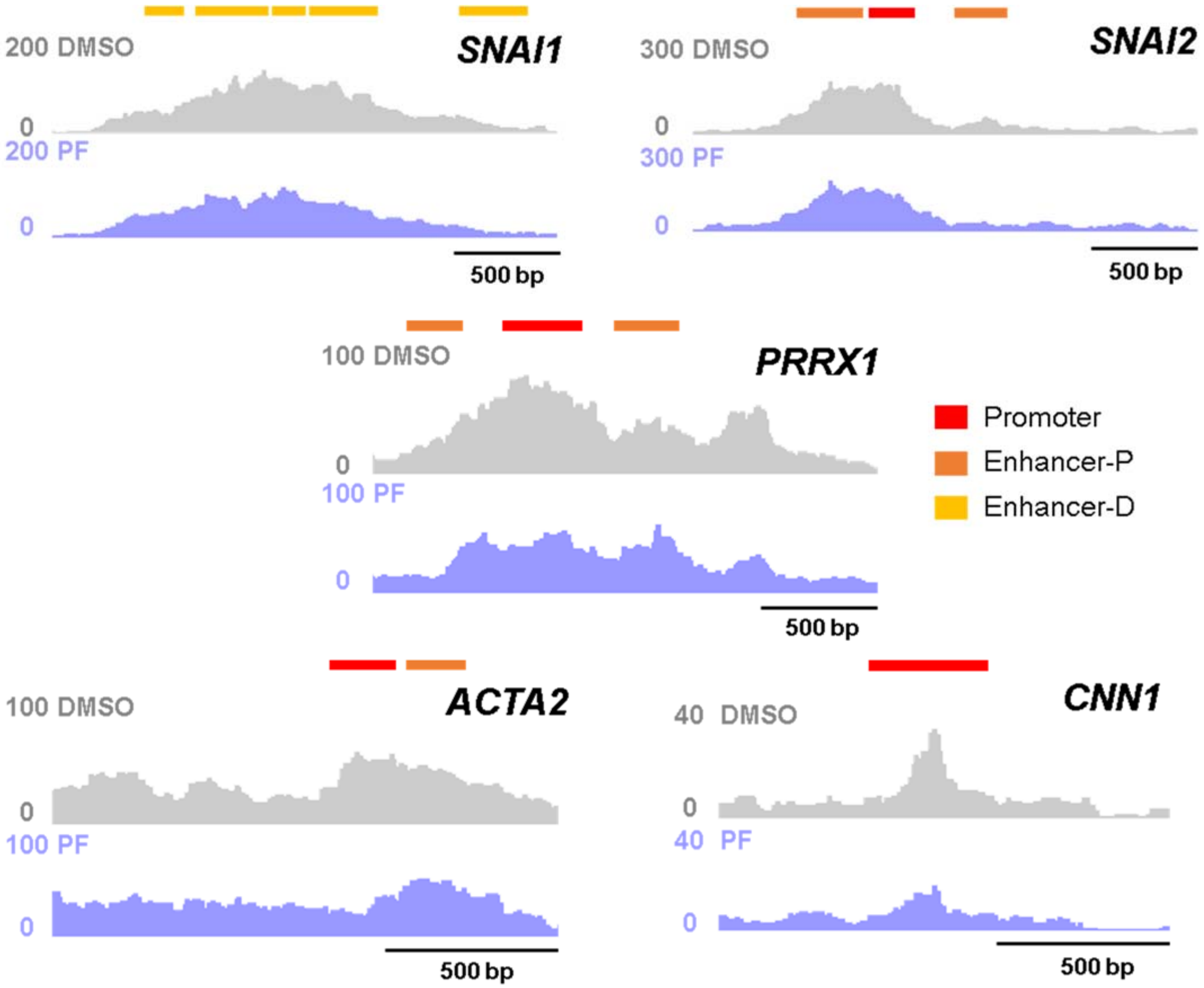

Figure S39. Disruption of cell adhesion altered chromatin accessibility around fibroblast-related genomic loci. ATAC-seq tracks for *SNAI1*, *SNAI2, PRRX1, ACTA2 and CNN1* genomic loci from fibroblasts treated with DMSO or 5 μM PF573228 for 2 hours. PF-induced chromatin remodeling occurred near promoter, proximal enhancer (Enhancer-P) and distal enhancer (Enhancer-D) sites.

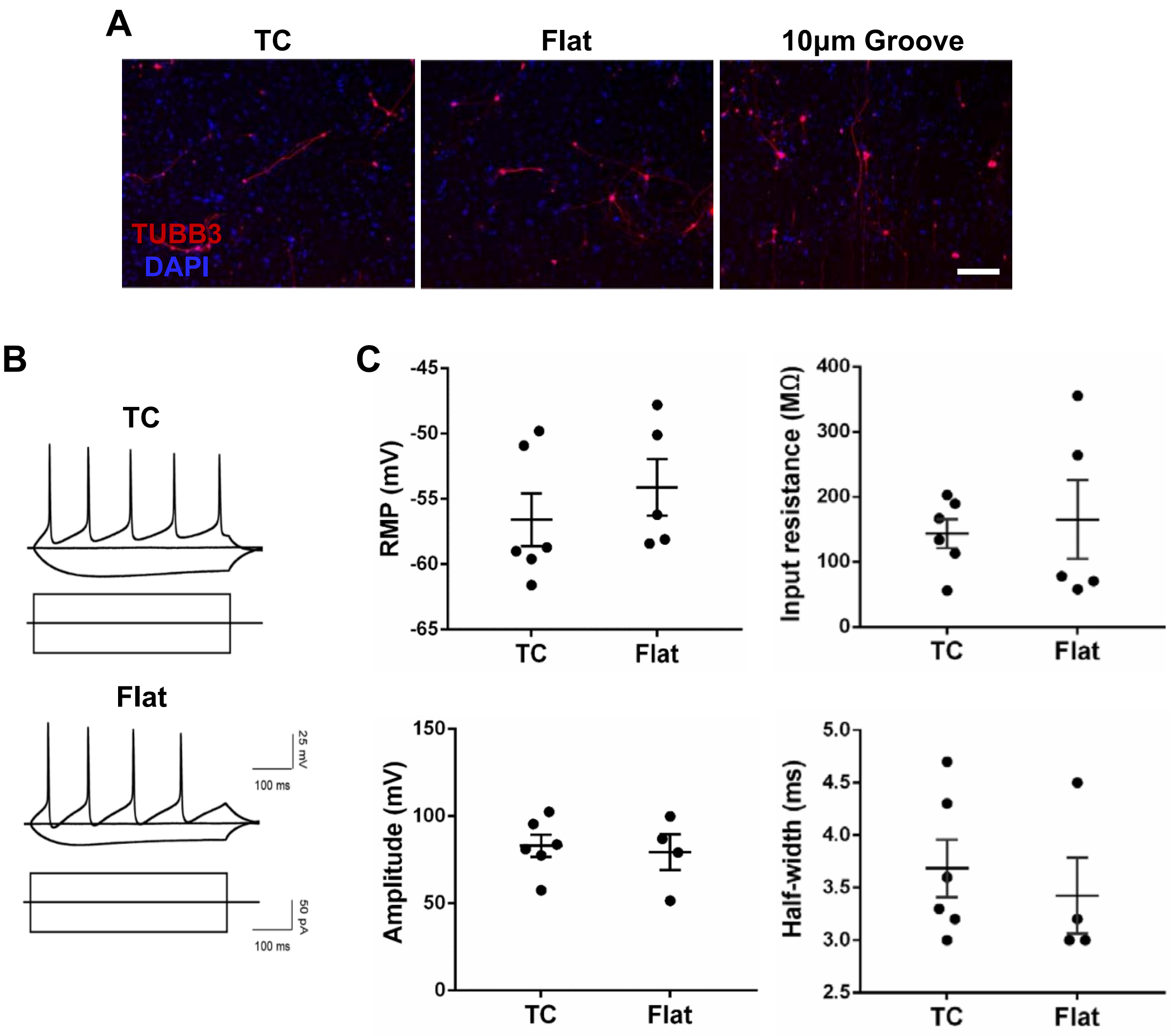

Figure S40. iN cells derived on different surfaces display functionality. A, Immunofluorescent images show that iN cells derived on TC treated wells (TC), flat PDMS membranes (Flat) and PDMS membranes with 10 µm grooves express β-tubulin III (TUBB3). Scale bar, 100 µm. **B,** Representative traces of spontaneous changes in membrane potential in response to current injection from iN cells obtained in TC wells or flat PDMS membranes. **C,** Quantification of electrophysiological properties of iN cells on TC-treated dishes (TC) or flat PDMS membranes (Flat). Each circle represents an individual cell that was tested. The resting membrane potential, RMP, input resistance and action potential amplitude and half-width were measured.

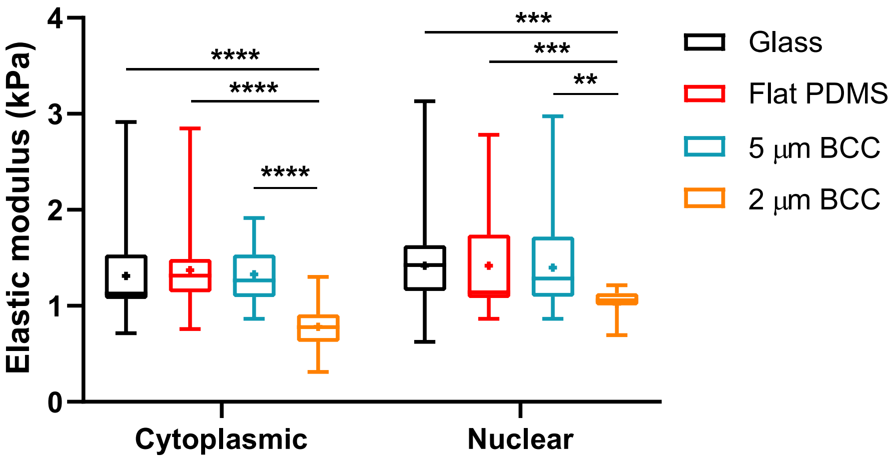

Figure S41. Effect of biomaterials on cell stiffness. Box plots illustrate the variation in elastic modulus of non-transduced fibroblasts cultured on glass, flat PDMS membranes, 5 µm binary colloidal crystals (BCC) and 2 µm BCCs as acquired using AFM (n=31-32 per condition). Significance determined by two-way ANOVA using Tukey's correction for multiple comparisons. Box plots show the ends at the quartiles, the median as a horizontal line in the box, the mean as a (+) symbol, and the whiskers extend from the minimum to maximum data point (**$p < 0.01$, ***$p < 0.001$, ****$p < 0.0001$).

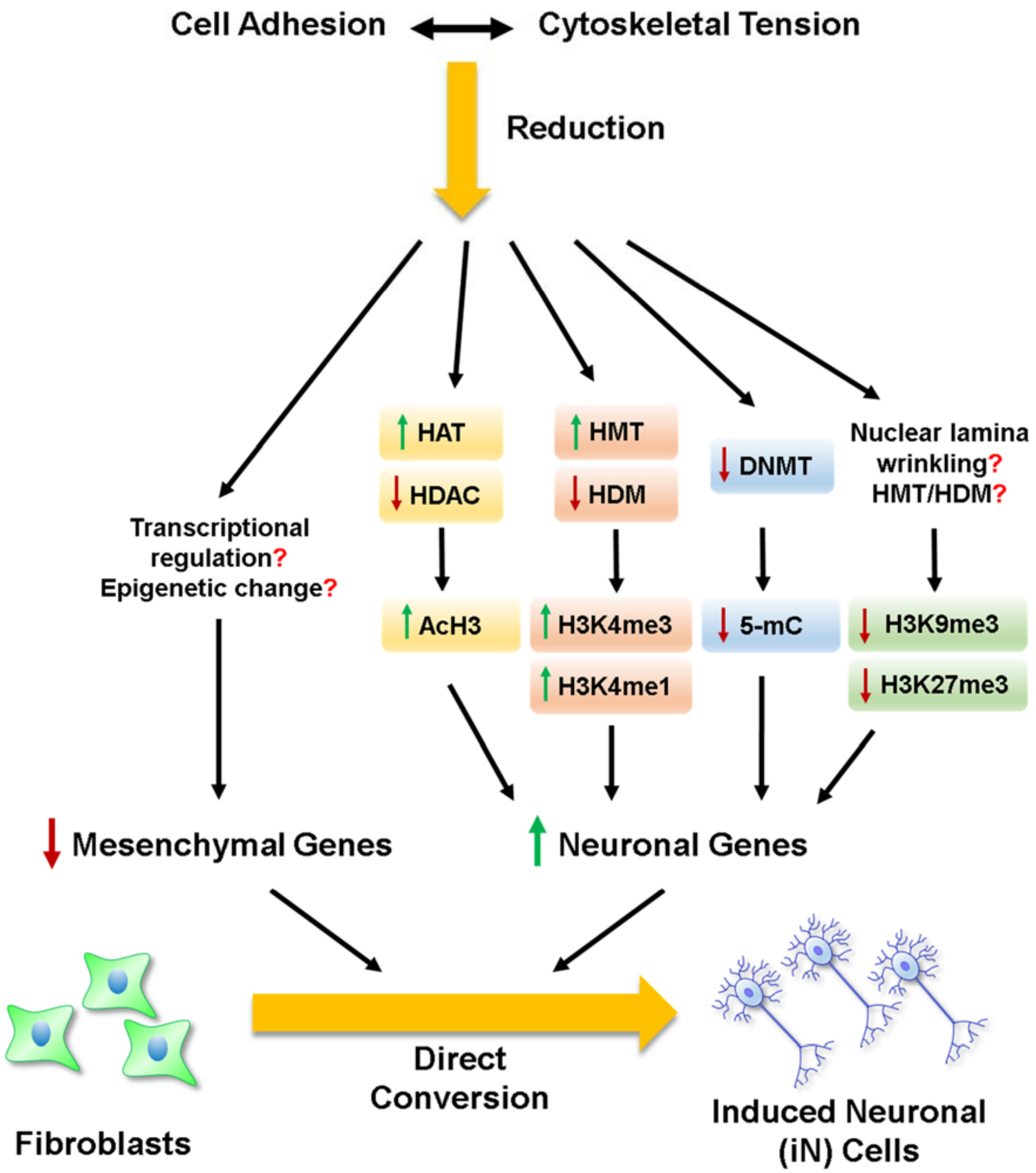

Figure S42. Summary on the role of the cytoskeleton and cell adhesion on the direct reprogramming of adult mouse fibroblasts into neurons. Disruption of cell adhesion and cytoskeletal tension modulates chromatin organization, the epigenetic state and neuronal gene expression to promote iN reprogramming.

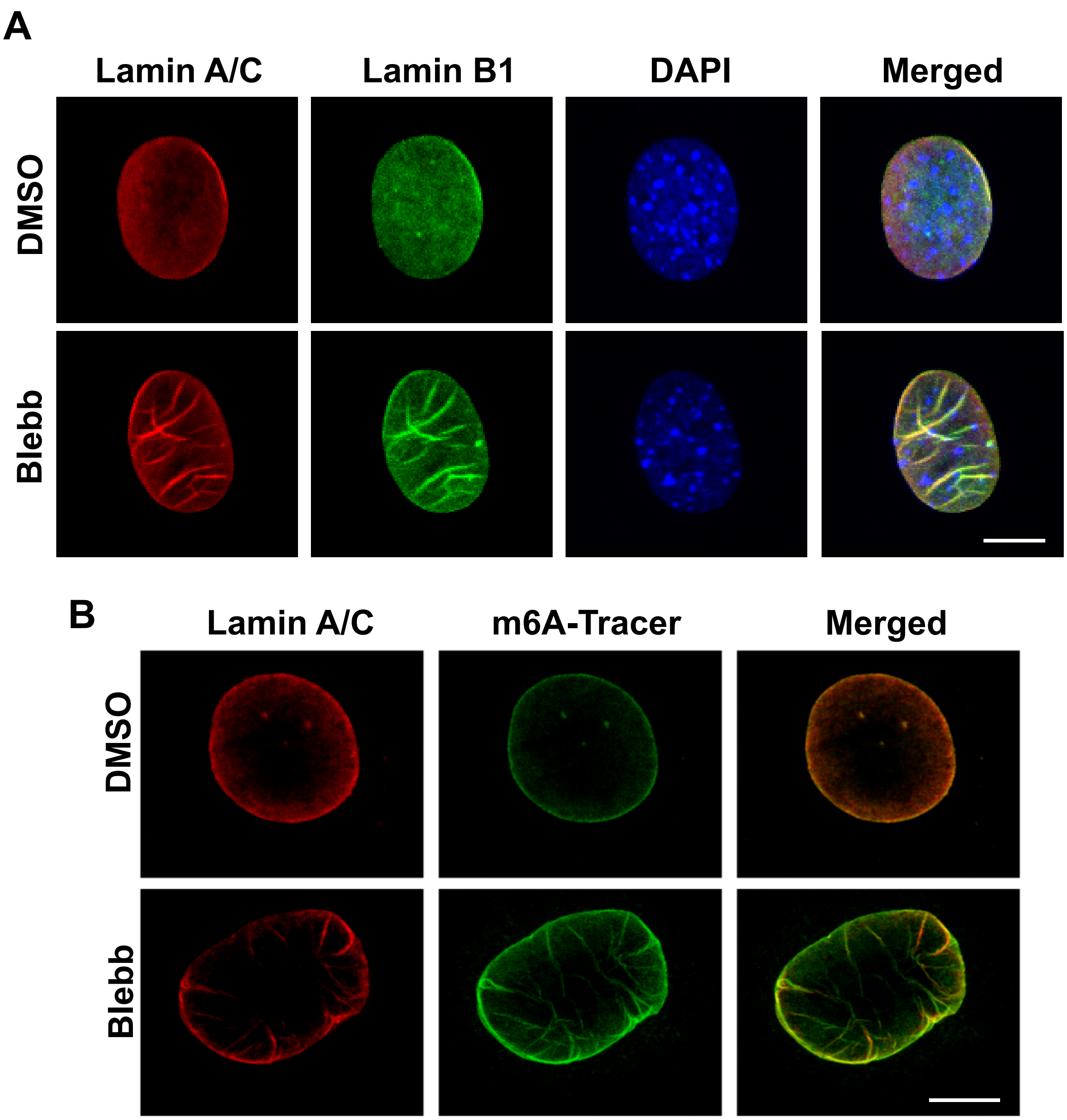

Figure S43. Blebbistatin disrupts the nuclear lamina. A, Immunofluorescent images of lamin A/C, lamin B1 and nuclei (DAPI) staining in non-transduced fibroblasts treated with 10 µm blebbistatin for two hours. Scale bar, 10 µm. **B,** Immunofluorescent images show lamin A/C and m6A-tracer in fibroblasts transfected with m6A-Tracer and Dam-Lamin B1 constructs and treated with vehicle or 10 µM blebbistatin for two hours. Scale bar, 10 µm.

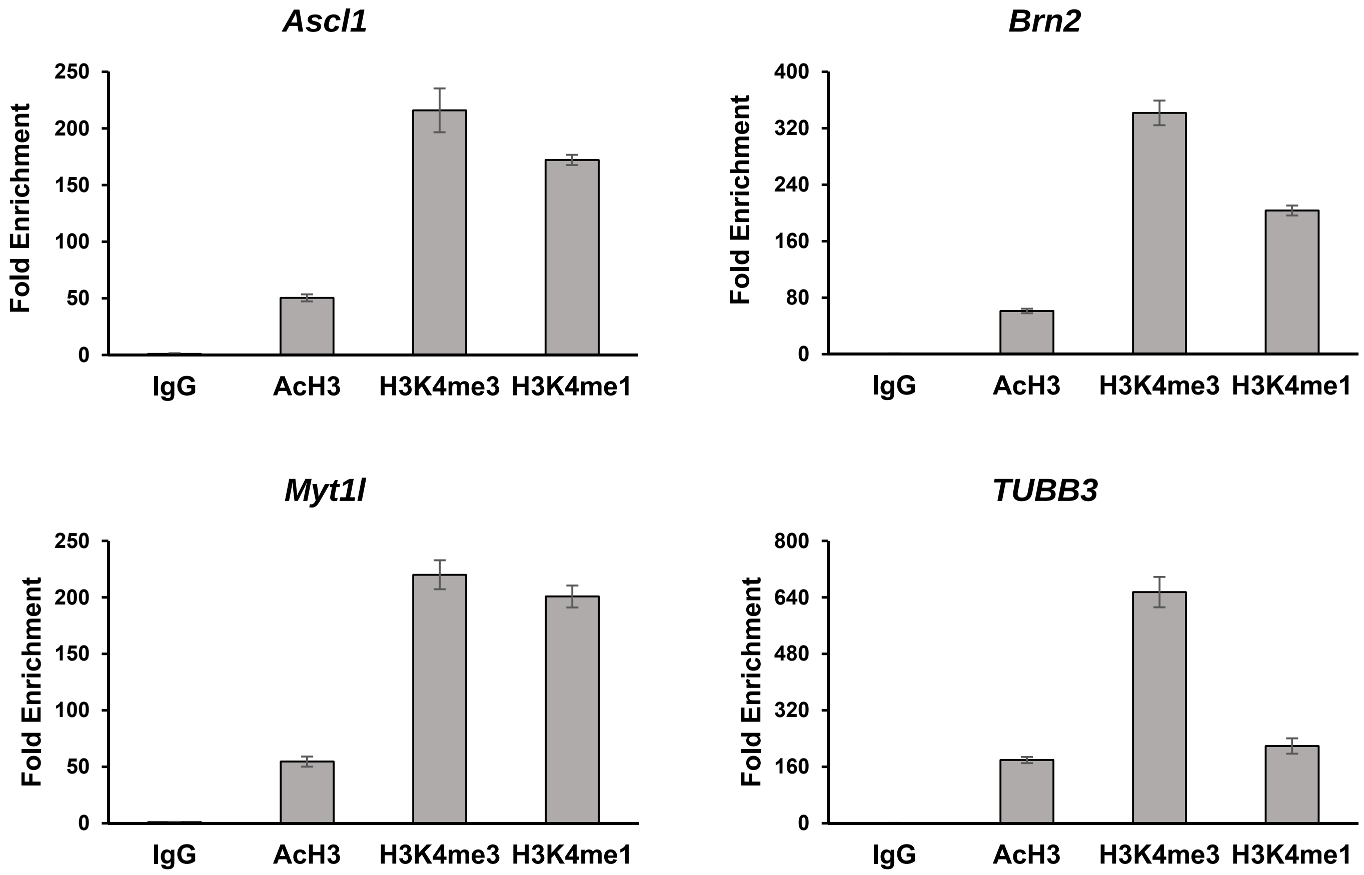

Figure S44. ChIP antibody fold enrichment. AcH3, H3k4me3 and H3k4me1 antibody fold enrichment over normal rabbit IgG control was quantified. Substantial enrichment was observed at the promoter regions of *Ascl1*, *Brn2*, *Myt1l*, and *TUBB3* genes following immunoprecipitation with antibodies against AcH3, H3k4me3 or H3k4me1.

Table S1. Antibody information for immunofluorescent staining and Western blotting analysis

Antibody	Vendor	Catalog #	Dilution	Application
TUBB3	Covance	MMS435P	1:1000	IF
TUBB3	Covance	MRB435P	1:1000	IF
NeuN	Covance	SIG39860	1:100	IF
MAP2	Sigma	M9942	1:200	IF
Synapsin	Abcam	ab64581	1:200	IF
VGlut1	Millipore	MAB5502	1:200	IF
GABA	Sigma	A2052	1:500	IF
pFAK	Abcam	ab81298	1:100	IF
AcH3	Millipore	06-599	1:200<br>1:1000	IF
H3K4me3	Millipore	04-473	1:300<br>1:1000	IF
H3K4me1	Abcam	ab8895	1:300	IF
H3K27me3	Millipore	51951	1:300	IF
H3K9me3	Abcam	ab176916	1:500	IF
5-mC	Cell Signaling	28692S	1:1000	IF
Paxillin	Abcam	ab32084	1:100<br>1:1000	IF<br>WB
Calponin	Abcam	ab46794	1:100<br>1:3000	IF<br>WB
αSMA	Abcam	ab32575	1:200<br>1:8000	IF<br>WB
pFAK	Cell Signaling	3283S	1:1000	WB
FAK	Cell Signaling	3285S	1:1000	WB
GAPDH	Santa Cruz	sc-32233	1:1500	WB
Actin	Santa Cruz	sc-1616	1:1000	WB
pERK	Cell Signaling	4370	1:1000	WB
ERK	Cell Signaling	4695	1:1000	WB
Lamin A/C	ThermoFisher	MA5-35284	1:200	IF
Lamin B1	Santa Cruz	sc-374015	1:50	IF
α-tubulin	Abcam	ab18251	1:3000	WB
Myosin IIa	Cell Signaling	49349S	1:1500	WB
Myosin IIb	Cell Signaling	8824S	1:1500	WB

Table S2. Primers used for qRT-PCR analysis

Gene Name	Forward Primer	Reverse Primer
Ascl1	GAAGCAGGATGGCAGCAGAT	TTTTCTGCCTCCCCATTTGA
Brn2	AGGGCGCAAACGGAAAA	GGCTTAGGGCATTTGAGGAAA
Myt1l	CTACAAGATGGACGTGGACTCTGA	GGAACTCGAACCCCTTTGG
TUBB3	GCGCCTTTGGACACCTATTC	CACCACTCTGACCAAAGATAAAGTT GT
MAP2	GCTGTGTGCTCCAAGTTTCA	AGCTGAGGAACCTTAATTCTTGC
Syn1	CAGCTCAACAAATCCCAGTCT	TCTCAGCTTTCACCTCGTCC
NeuroD1	CACGCAGAAGGCAAGGTGTC	TTTGGTCATGTTTCCACTTCCTGT
CNN1	CATCGGGAACAACTTCATGG	CTATGTTCTCCAGCTGGTGC
ACTA2	ATGACCCAGATTATGTTTGAGACC	AATACCAGTTGTACGTCCAGAG
ELN	GGATAAAACGAGGCGCTGAGA	GGAACCCCTCCAGGCTGC
DCN	TACCCGGATTAAAAGGTCGTG	CCCAAGAGACTTGTGCCAGA
SNAI1	ACTGGTGAGAAGCCATTCTC	TGGCACTGGTATCTCTTCAC
VIM	CTTAAAGGCACTAACGAGTCC	AATTCTCTTCCATCTCACGCA
MYH9	AGAACAAACTGAGGCGTGGG	CTTTACCGTCGACCTCCTCG
MYH10	GGTGGGAGTGGGTGGG	AAAAGTAATTGCCTCTTCAGCC
PTK2	ATGCCCTCGACCAGGGATTA	AAGCTGGATTCTCTGGGCTC
18S	GCCGCTAGAGGTGAAATTCTTG	CATTCTTGGCAAATGCTTTCG

Table S3. Primers used for ChIP qRT-PCR analysis

Gene Name	Forward Primer	Reverse Primer
Ascl1	AACCCCATATGGCTGCAGAG	GGGAGAGCGTTTGCACACTA
Brn2	GCGCCCGAGGGAAGAAGAG	GCTCTGCGCCCTTTGATTTAC
Myt1l	GAGGCCATCTGTTGTGGAAAA	AAATCCCCGGCATATGACCTT
TUBB3	AGAGGTCTCAAGAAGGGTTTCGC	AGAGGGTCTTTCTTTCTCTCAAGTG
